# Language models, like humans, show content effects on reasoning tasks

**DOI:** 10.1093/pnasnexus/pgae233

**Published:** 2024-07-16

**Authors:** Andrew K Lampinen, Ishita Dasgupta, Stephanie C Y Chan, Hannah R Sheahan, Antonia Creswell, Dharshan Kumaran, James L McClelland, Felix Hill

**Affiliations:** Google DeepMind, Mountain View, CA, 94043 USA; Google DeepMind, Mountain View, CA, 94043 USA; Google DeepMind, Mountain View, CA, 94043 USA; Google DeepMind, London N1C 4DN, UK; Google DeepMind, London N1C 4DN, UK; Google DeepMind, London N1C 4DN, UK; Google DeepMind, Mountain View, CA, 94043 USA; Stanford University, Stanford, CA 94306, USA; Google DeepMind, London N1C 4DN, UK

**Keywords:** language models, content effects, reasoning, logic, cognitive science

## Abstract

Abstract reasoning is a key ability for an intelligent system. Large language models (LMs) achieve above-chance performance on abstract reasoning tasks but exhibit many imperfections. However, human abstract reasoning is also imperfect. Human reasoning is affected by our real-world knowledge and beliefs, and shows notable “content effects”; humans reason more reliably when the semantic content of a problem supports the correct logical inferences. These content-entangled reasoning patterns are central to debates about the fundamental nature of human intelligence. Here, we investigate whether language models—whose prior expectations capture some aspects of human knowledge—similarly mix content into their answers to logic problems. We explored this question across three logical reasoning tasks: natural language inference, judging the logical validity of syllogisms, and the Wason selection task. We evaluate state of the art LMs, as well as humans, and find that the LMs reflect many of the same qualitative human patterns on these tasks—like humans, models answer more accurately when the semantic content of a task supports the logical inferences. These parallels are reflected in accuracy patterns, and in some lower-level features like the relationship between LM confidence over possible answers and human response times. However, in some cases the humans and models behave differently—particularly on the Wason task, where humans perform much worse than large models, and exhibit a distinct error pattern. Our findings have implications for understanding possible contributors to these human cognitive effects, as well as the factors that influence language model performance.

**Significance Statement**

Language models and humans both mix semantic content into their performance on logical reasoning problems, which generally results in greater success in familiar situations, but more errors in unusual ones. These results may inform the search for the origins of these human behaviors and may help improve applications of language models.

## Introduction

A hallmark of abstract reasoning is the ability to perform systematic operations over variables that can be bound to any entity (1, 2): the statement: “X is bigger than Y” logically implies that “Y is smaller than X”, no matter the values of X and Y. That is, abstract reasoning is ideally content-independent (2). The capacity for reliable and consistent abstract reasoning is frequently highlighted as a crucial missing component of current AI (3–5). For example, while large language models (LMs) exhibit some impressive *emergent* behaviors, including some performance on abstract reasoning tasks ((6–9); though cf. (10)), they have been criticized for failing to achieve systematic consistency in their abstract reasoning (e.g. (11–13)).

However, humans—arguably the best known instances of general intelligence—are far from perfectly rational abstract reasoners (14, 15). Patterns of biases in human reasoning have been studied across a wide range of tasks and domains (15). Here, we focus on “content effects”—the finding that humans are affected by the semantic content of a logical reasoning problem. In particular, humans reason more readily and more accurately about familiar, believable, or grounded situations, compared to unfamiliar, unbelievable, or abstract ones. For example, when presented with a syllogism like the following:

All students read.

Some people who read also write essays.

Therefore some students write essays.

humans will often classify it as a valid argument. However,

All students read.

Some people who read are professors.

Therefore some students are professors.

is much less likely to be considered valid (14, 16, 17)—despite the fact that the arguments above are logically equivalent (both are invalid). Similarly, humans struggle to reason about how to falsify conditional rules involving abstract attributes (18, 19), but reason more readily about logically equivalent rules grounded in realistic situations (20, 21). This human tendency also extends to other forms of reasoning e.g. probabilistic reasoning, where humans are notably worse when problems do not reflect intuitive expectations (22).

The literature on human cognitive biases is extensive, but many of these biases can be idiosyncratic and setting-dependent. For example, even some seminal findings in the influential work of Kahneman et al. (15), like “base rate neglect,” are sensitive to setting and experimental design (23). However, the content effects on which we focus are a notably consistent finding that has been documented in humans across many reasoning tasks and domains: deductive, inductive, logical, or probabilistic (14, 19, 22, 24, 25). This consistent content sensitivity contradicts the definition of abstract reasoning—that it is independent of content. This tension speaks to longstanding debates over the fundamental nature of human intelligence: are we best described as algebraic symbol-processing systems (2, 26), or emergent connectionist ones (27, 28) whose inferences are grounded in learned semantics? Explanations or models of content effects in cognitive science often focus on a single (task and content-specific) phenomenon and invoke bespoke mechanisms that only apply to these specific settings (e.g. (21)). A more general understanding of what leads to this blending of logical and semantic content is lacking.

In this work, we examine whether LMs also blend semantic content with logic. LMs possess prior knowledge that is shaped by their training. Indeed, the goal of the “pretrain and adapt” or “foundation models” (29) paradigm is to endow a model with broad prior knowledge for later tasks. Thus, LM representations often *reflect* human semantic cognition; e.g. language models reproduce patterns like association and typicality effects (30, 31), and LM predictions can reproduce human knowledge and beliefs (32, 33). Here, we explore whether this prior knowledge impacts LM performance in logical reasoning tasks. While various recent works have explored biases and imperfections in language models’ performance (e.g. (12, 13, 34, 35)), we focus specifically on whether content interacts with logic in these systems in the ways it does for humans. Specifically, we hypothesize that while LMs and humans will not always show identical answer patterns, LMs will show directionally similar effects to those observed in humans. In particular, we test whether LMs show facilitation when semantic content supports the logical answer, and interference when it does not. This question has significant implications both for characterizing LMs and potentially for understanding human cognition, contributing new ways of understanding the balance, interactions, and trade-offs between the abstract and grounded capabilities of a system and suggesting new ways of thinking about the potential nature of the mechanisms at play in the human case.

**Fig. 1.**
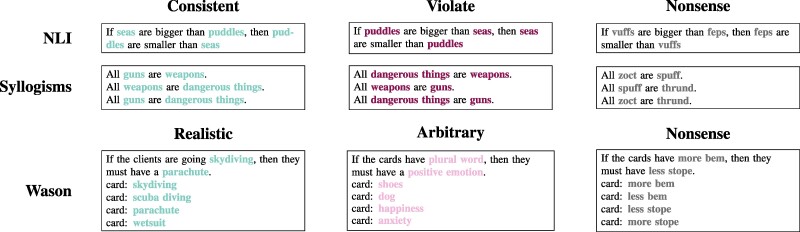
Manipulating content within fixed logical structures. In each of our three datasets (rows), we instantiate different versions of the logical problems (columns). Different versions of a problem offer the same logical structures and tasks, but instantiated with different entities or relationships between those entities. The relationships in a task may either be consistent with, or violate real-world semantic relationships, or may be nonsense, without semantic content. In general, humans and models reason more accurately about belief-consistent or realistic situations or rules than belief-violating or arbitrary ones. (For brevity this figure presents a subset of the problem text; complete example problems are included in Fig. S9).

We explore how the content of logical reasoning problems affects the performance of a range of large LMs (36–38). To avoid potential dataset contamination, we create entirely new datasets using designs analogous to those used in prior cognitive work, but with a variety of semantic instantiations that have not been used in prior research, and also collect directly comparable human data with our new stimuli. We find that LMs reproduce the direction of human content effects across three different logical reasoning tasks (Fig. 1). We first explore a simple natural language inference (NLI) task and show that models and humans answer fairly reliably, with relatively modest influences of content. We then examine the more challenging task of judging whether a syllogism is valid, finding that models and humans are both biased by the believability of the conclusion. We finally consider realistic and abstract/arbitrary versions of the Wason selection task (19)—a task introduced over 50 years ago that demonstrates a failure of systematic human reasoning—and show that LMs and humans perform better with a realistic framing. Our findings with human participants replicate and extend existing findings in the cognitive literature. We also report novel analyses of item-level effects, and the effect of content and items on continuous measures of model and human responses. Finally, we note a number of cases where the humans and models do not align, for example on the difficult Wason task, where large LMs generally outperform humans and their error patterns differ. We close with a discussion of the possible implications of these findings for understanding human cognition as well as the performance of language models.

### Evaluating content effects on logical tasks

We evaluate content effects on three logical reasoning tasks (depicted in Fig. 1). These three tasks involve different types of logical inferences, and different kinds of semantic content. However, these distinct tasks admit a consistent definition of content effects: the extent to which reasoning is facilitated when the semantic content supports the correct logical inference, and correspondingly the extent to which reasoning is harmed when semantic content conflicts with the correct logical inference (or, in the Wason tasks, when the content is simply arbitrary). We also evaluate versions of each task where the semantic content is replaced with nonsense nonwords, which lack semantic content and thus should neither support nor conflict with reasoning performance. (However, note that in some cases, particularly the Wason tasks, changing to nonsense content requires more substantially altering the kinds of inferences required in the task; see Materials and methods.)

####  

##### Natural language inference

The first task we consider has been studied extensively in the natural language processing literature (39). In the classic NLI problem, a model receives two sentences, a “premise” and a “hypothesis”, and has to classify them based on whether the hypothesis “entails”, ‘contradicts’, or “is neutral to” the premise. Traditional datasets for this task were crowd-sourced (40) leading to sentence pairs that don’t strictly follow logical definitions of entailment and contradiction. To make this task more strictly logical, we follow Dasgupta et al. (41) to generate comparisons (e.g. X is smaller than Y). We then give participants an incomplete inference such as “If puddles are bigger than seas, then…” and ask them to choose between two possible hypotheses to complete it: “seas are bigger than puddles” and “seas are smaller than puddles.” Note that one of these completions is consistent with real-world semantic beliefs i.e. “believable”, but is logically inconsistent with the premise, while the other is logically consistent with the premise but contradicts real world beliefs—thus, logical consistency and believability can be manipulated independently. We can then evaluate whether models and humans answer more accurately when the logically correct hypothesis is believable; that is, whether the content affects their logical reasoning. We hypothesized that both LMs and humans would perform relatively well on these simple tasks—and thus that both would show minimal content effects.

However, content effects are generally more pronounced in difficult tasks that require extensive logical reasoning (16, 24). We therefore consider two more challenging tasks where human content effects have been observed in prior work.

##### Syllogisms

Syllogisms (42) are a simple argument form in which two true statements necessarily imply a third. For example, the statements “All humans are mortal” and “Socrates is a human” together imply that “Socrates is mortal.” But human syllogistic reasoning is not purely abstract and logical; instead it is affected by our prior beliefs about the contents of the argument (14, 17, 43). For example, Evans et al. (14) showed that if participants were asked to judge whether a syllogism was logically valid or invalid, they were biased by whether the conclusion was consistent with their beliefs. Participants were very likely (90% of the time) to mistakenly say an invalid syllogism was valid if the conclusion was believable, and thus mostly relied on belief rather than abstract reasoning. Participants would also sometimes say that a valid syllogism was invalid if the conclusion was not believable, but this effect was somewhat weaker (but cf. (44)). These “belief-bias” effects have been replicated and extended in various subsequent studies (17, 44). We therefore hypothesized that language models would likewise be more likely to endorse an argument as valid if its conclusion is believable, or to dismiss it as invalid if its conclusion is unbelievable.

##### The Wason selection task

The Wason selection task (19) is a logic problem that can be challenging even for humans with substantial education in logic. Participants are shown four cards, and are told that each card has a letter on one side, and a number on the other. The participants are then told a rule such as: “if a card has a ‘D’ on one side, then it has a ‘3’ on the other side”. The four cards respectively show “D”, “F”, “3”, and “7”. The participants are then asked which cards they need to flip over to check if the rule is true or false. The correct answer is to flip over the cards showing “D” and “7”. However, Wason (19) showed that while most participants correctly chose “D”, they were much more likely to choose “3” than “7”. That is, the participants should check the *contrapositive* of the rule (“not 3 implies not D”, which is logically implied), but instead they confuse it with the *converse* (“3 implies D”, which is not logically implied). Furthermore, Wason even attempted instructing the subjects not to assume the converse, using examples and explanations, but even with this extra instruction very few subjects produced completely correct responses. This is therefore a classic task in which reasoning according to the rules of formal logic does not come naturally for humans, and thus there is potential for prior beliefs and knowledge to affect reasoning.

Indeed, the difficulty of the Wason task depends upon the content of the problem. Past work has found that if an identical logical problem is instantiated in a common situation, particularly a social rule, participants are much more accurate (20, 21, 45). For example, if participants are told the cards represent people, and the rule is “if they are drinking alcohol, then they must be 21 or older” and the cards show “beer”, “soda”, “25”, “16”, then many more participants correctly choose to check the cards showing “beer” and “16”. We therefore hypothesized that LMs would similarly show facilitated reasoning about realistic rules, compared to the more-abstract arbitrary ones. (Note that in our Wason task implementations, we forced participants and LMs to choose exactly two cards, in order to align answer formats between the humans and LMs.) However, we note that other studies have found that the facilitation can be quite sensitive to the particular rule evaluated; several studies found poor performance even with seemingly plausible rules (46, 47). The extent of content effects on the Wason task also depends upon background knowledge; mathematical education is associated with improved reasoning on abstract Wason tasks (48, 49). However, even experienced participants are far from perfect—undergraduate mathematics majors and academic mathematicians achieved less than 50% accuracy at the arbitrary Wason task (49). This illustrates the challenge of abstract logical reasoning, even for experienced humans. As we will see in the next section, many human participants did struggle with our versions of these tasks.

## Results

**Fig. 2.**
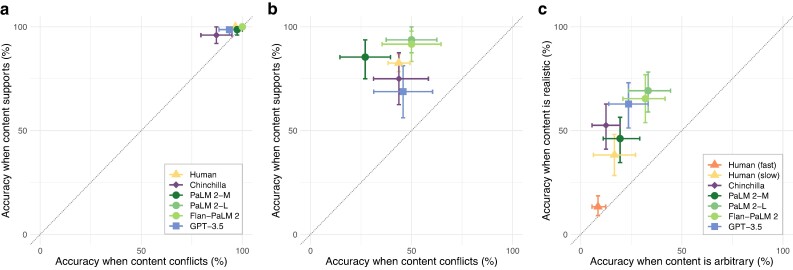
Across the three tasks we consider, various language models and humans show similar patterns of overall accuracy and directions of content effects on reasoning. The vertical axis shows accuracy when the content of the problems supports the logical inference. The horizontal axis shows accuracy when the content conflicts (or, in the Wason task, when it is arbitrary). Thus, points above the diagonal indicate an advantage when the content supports the logical inference. a) On basic NLIs, both humans and LMs demonstrate high accuracy across all conditions, and thus relatively little effect of content. b) When identifying whether syllogisms are logically valid or invalid, both humans and LMs exhibit moderate accuracy, and substantial content effects. c) On the Wason selection task, the majority of humans show fairly poor performance overall. However, the subset of subjects who take the longest to answer show somewhat higher accuracy, primarily on the realistic tasks—i.e. substantial content effects. On this difficult task, LMs generally exceed humans in both accuracy and magnitude of content effects. (Throughout, errorbars are bootstrap 95% CIs).

We summarize our primary results in Fig. 2. In each of our three tasks, humans and models show similar levels of accuracy across conditions. Furthermore, in keeping with our hypothesis, humans and models show similar content effects on each task, which we measure as the advantage when reasoning about logical situations that are consistent with real-world relationships or rules. In the simplest NLI task, humans and all models show high accuracy and relatively minor effects of content. When judging the validity of syllogisms, both humans and models show more moderate accuracy, and significant advantages when content supports the logical inference. Finally, on the Wason selection task, humans and models show even lower accuracy, and again substantial content effects. We describe each task, and the corresponding results and analyses, in more detail below.

###  

####  

##### Natural language inference

**Fig. 3.**
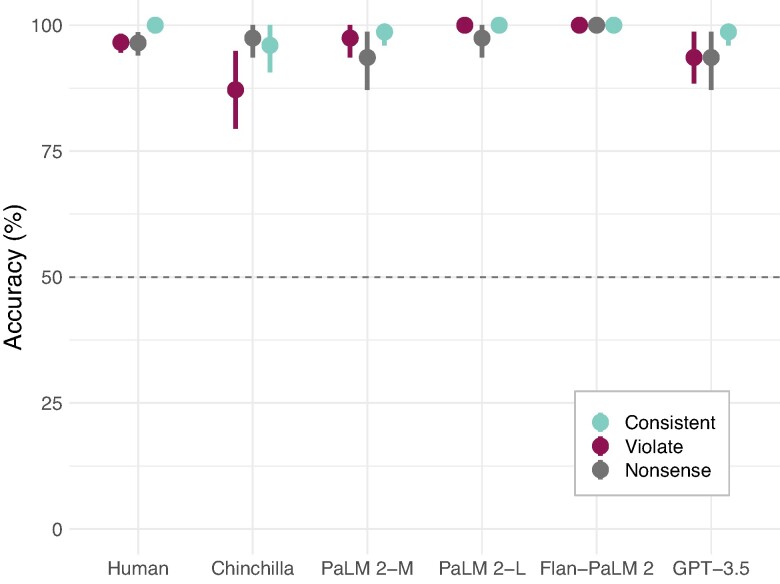
Detailed results on the NLI tasks. Both humans (left) and all models show relatively high performance, and relatively little difference in accuracy between belief-consistent and belief-violating inferences, or even nonsense ones.

The relatively simple logical reasoning involved in this task means that both humans and LMs exhibit high performance, and correspondingly show relatively little effect of content (Fig. 3). We do not detect a statistically significant effect of content on accuracy in humans or any of the LMs in mixed-effects logistic regressions controlling for the random effect of items (or $\chi^{2}$ tests where regressions did not converge due to ceiling effects; all z<1.21 or $\chi^{2}<0.1$, all P>0.2; see Supplementary Material C.1 for full results). However, we do find a statistically significant relationship between human and model accuracy at the item level (t(832)=3.49, P<0.001; Fig. S30—even when controlling for condition. Furthermore, as we discuss below, further investigation into the model confidence shows evidence of content effects on this task as well.

##### Syllogisms

**Fig. 4.**
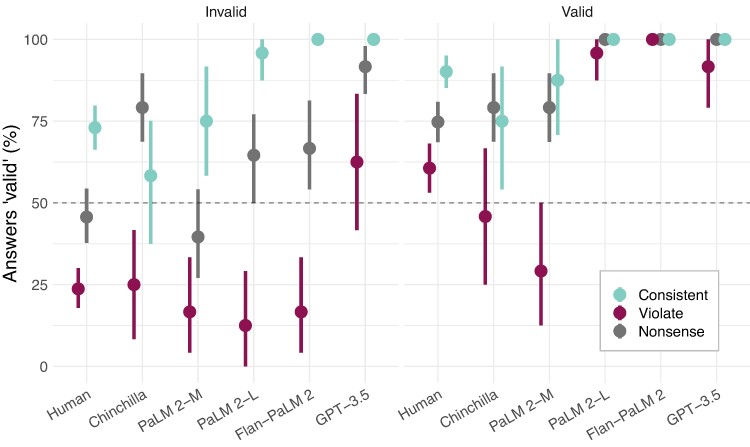
Detailed results on syllogism tasks. The vertical axis shows the proportion of the time that each system answers that an argument is valid. Both humans and models exhibit substantial content effects—a strong bias towards saying an argument is valid if the conclusion is consistent with expectations (cyan), and some bias towards saying the argument is invalid if the conclusion violates expectations (maroon). (This figure plots the proportion of the time the humans or models answer “valid” rather than raw accuracy, to more clearly illustrate the bias. To see accuracy, reverse the vertical axis for the invalid arguments).

Syllogism validity judgements are significantly more challenging than the NLI task above; correspondingly, both humans and LMs exhibit lower accuracy. Nevertheless, humans and most LMs are sensitive to the logical structure of the task. However, both humans and LMs are strongly affected by the syllogism content (Fig. 4), as in prior human studies (16). If the semantic content supports the logical inference—that is, if the conclusion is believable and the argument is valid, or if the conclusion is unbelievable and the argument is invalid—humans and all LMs tend to answer more accurately (all z≥2.25 or $\chi^{2}>6.39$, all P≤0.01; see Supplementary Material C.2 for full results).

Two simple effects contribute to this overall content effect: that belief-consistent conclusions are judged as logically valid and that belief-inconsistent conclusions are judged as logically invalid. As in prior works, the dominant effect is that humans and models tend to say an argument is valid if the conclusion is belief-consistent, regardless of the actual logical validity. If the conclusion is belief-violating, humans and models do tend to say it is invalid more frequently, but are generally more sensitive to actual logical validity in this case. Specifically, we observe a significant interaction between the content effect and conclusion believability in humans, PaLM 2-L, Flan-PaLM 2, and GPT-3.5 (all z>5.9 or $\chi^{2}>14.3$, all P<0.001); but do not observe a significant interaction in Chinchilla or PaLM 2-M (both $\chi^{2}<0.001$, P>0.99). Both humans and models appear to show a slight bias towards saying syllogisms with nonsense words are valid, but again with some sensitivity to logic.

Furthermore, even when controlling for condition, we find a significant correlation between item-level accuracy in humans and LMs (t(345)=4.98, P<0.001), suggesting shared patterns in the use of lower-level details of the logic or content.

##### The Wason selection task

**Fig. 5.**
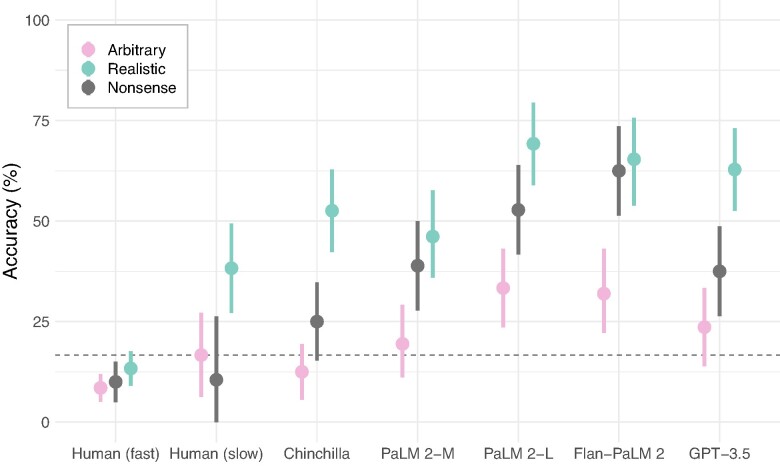
Detailed results on the Wason task. Human performance is low, even on Realistic rules. However, the subset of subjects who answer more slowly show above chance accuracy for the realistic rules (cyan), but not for the arbitrary ones (pink). Furthermore, each of the language models reproduces this pattern of advantage for the realistic rules. In addition, two of the larger models perform above chance at the arbitrary rules. (The dashed line corresponds to chance—a random choice of two cards. LMs and humans were forced to choose exactly two cards).

As in the prior human literature, we found that the Wason task was relatively challenging for humans, as well as for language models (Fig. 5). Nevertheless, we observed significant content advantages for the Realistic tasks in humans, and in Chinchilla, PaLM 2-L, and GPT-3.5 (all z>2.2, all P<0.03; Supplementary Material C.3). We only observed marginally significant advantages of realistic rules in PaLM 2-M and Flan-PaLM 2 (both z≥1.78, both P≤0.08), due to stronger item-level effects in these models (though the item-level variance does not seem particularly unusual; see Supplementary Material B.8.3). Intriguingly, some language models also show better performance at the versions with Nonsense nouns compared to the Arbitrary ones, though generally Realistic rules are still easier. We also consider several rule variations in Fig. S25.

**Fig. 6.**
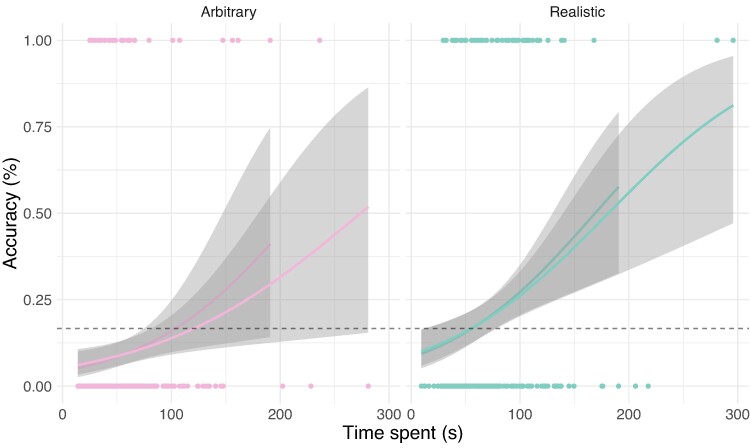
There is a strong relationship between response time and answer accuracy in the Wason tasks; subjects who take longer to answer are more accurate on average. Participants who take sufficiently long to answer perform above chance in the Realistic tasks. There are hints of a similar effect in the Arbitrary condition, but we do not have the power to detect it. (Curves are logistic regression fits, with 95% CIs. We also plot regressions dropping outliers with time greater than 180 s, to show that the effect is not driven solely by outliers).

Our human participants struggled with this task, as in prior research, and did not achieve significantly above-chance performance overall (although their behavior is not random; see below). However, spending longer on logical tasks can improve performance (50), and thus many studies split analyses by response time (51). Indeed, human accuracy was positively and significantly associated with response time (z=4.44, P<0.001; Supplementary Material C.3.1 see Fig. 6. To visualize these reaction time effects in our Figs. 2 and 5, we split subjects into “slow” and “fast” groups. The distribution of times taken by subjects is quite skewed, with a long tail. We separate out the top 15% of subjects that take the longest, who spent more than 80 s on the problem, as the slow group. These subjects showed above chance performance in the Realistic condition, but still performed near chance in the other conditions. Intriguingly, we also find that chain-of-thought techniques (loosely giving the models time to “think”) can improve the performance of strong models on the Arbitrary and Nonsense conditions of the Wason task (Supplementary Material B.3). In subsequent sections (and Supplementary Material B.7.1) we further investigate the predictive power of human response times on the other tasks.

We collected the data for the Wason task in two different experiments; after observing the lower performance in the first sample, we collected a second sample where we offered a performance bonus for this task. We did not observe significant differences in overall performance or content effects between these subsets, so we collapse across them in the main analyses; however, we present results for each experiment and some additional analyses in Supplementary Material B.6.

##### Robustness of results to altering prompting and scoring

LM behavior is frequently sensitive to evaluation details. Thus, we performed several experiments to confirm that our results were robust to the methods used. We outline these experiments here; seein Supplementary Material B.2 for full results. First, removing the prequestion instructions does not substantially alter the overall results (Supplementary Material B.2.1). Next, the DC-PMI scoring correction is not the primary driver of content effects (Supplementary Material B.2.2). Finally, we consider few-shot evaluation. Giving few-shot examples yields some mild improvements in accuracy (with greater improvement in the simpler tasks) but does not eliminate content effects (Supplementary Material B.2.4). Together, these experiments suggest that our findings are not strongly driven by idiosyncratic details of our evaluation and thus support the robustness of our findings.

##### Chain-of-thought can sometimes push large models to rely more on logic

Chain-of-thought methods (52) have been shown to improve performance on some reasoning tasks, by allowing the models to produce a reasoning trace before giving their final answer. We tested the benefits of these strategies (Supplementary Material B.3), and find that chain-of-thought prompting can, in some cases, push large models to rely more on logical strategies, thereby reducing content effects through improving performance on less familiar or conflicting situations—particularly if those examples demonstrate precisely the type of reasoning that’s required.

##### Variability across different language models

We generally find similar content effects across the various models we evaluate, but there are a few notable differences. First, across tasks larger models tend to be more accurate overall (e.g. comparing the large vs. the medium variants of PaLM 2); however, this does not necessarily mean they show weaker content effects. While it might be expected that instruction-tuning would affect performance, the instruction-tuned models (Flan-PaLM 2 and GPT-3.5-turbo-instruct) do not show consistent differences in overall accuracy or content effects across tasks compared to the base language models—in particular, Flan-PaLM 2 performs quite similarly to PaLM 2-L overall. (However, there are some more notable differences in the distributions of log-probabilities from the instruction-tuned models; Supplementary Material B.10.)

On the syllogisms, there are some noticeable differences. GPT-3.5, and the larger PaLM 2 models, have high sensitivity for identifying valid arguments but relatively less specificity. By contrast, PaLM 2-M and Chinchilla models rely more on content rather than logic; i.e. they judge consistent conclusions as more valid than violating ones, irrespective of their logical structure. The sensitivity to logic in the nonsense condition also varies across models—the PaLM models are fairly sensitive, while GPT 3.5 and Chinchilla both have a strong bias to answer “valid” to all nonsense propositions irrespective of logic.

On the Wason task, the main difference of interest is that the PaLM 2 family of models show generally greater accuracy on the nonsense problems than the other models do, comparable to their performance on the Realistic condition in some cases.

In Supplementary Material B.3, we also test several newer Gemini (53) models on the Syllogisms and Wason tasks, and observe similar effects to the above, showing that these phenomena still hold with more recent models. We also test the smaller, open-source Gemma 7B model (54), which shows low performance overall, with strong content biases on the syllogisms task. Thus, at least with present models, a fairly large scale may be needed to observe significant differences in performance modulated by content on complex logic problems like the Wason task, but simpler content effects may be observed even at smaller scales.

### Model confidence relates to human response times

LMs do not produce a single answer, but a probability distribution over the possible answers. This distribution can provide further insight into their processing. For example, the probability assigned to the top answers, relative to the others, can be used as a confidence measure. By this measure, LMs are often somewhat calibrated; i.e. the probability they assign to the top answer approximates the probability that top answer is correct (e.g. (55)). Human response times (RTs) relate to similar variables, including confidence, surprisal, and task difficulty. Thus, prior works have related LM confidence to human response times for linguistic stimuli (e.g. (56)). Here, we correspondingly analyze how LM confidence relates to the task content and logic, answer correctness, and human RTs.

**Fig. 7.**
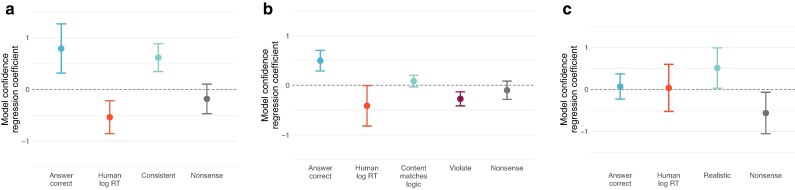
Language model confidence—as measured by the difference in(prior-corrected) log-probability between the chosen answer and the next most probable—is associated with correct answers, task variables, and human average response times. a, b) On the NLI and syllogism tasks, models are generally more confident in correct answers and belief-consistent conditions, less confident in belief-violating conditions, and less confident on problems that humans take longer to answer. c) On the Wason task, effects are weaker. Human RT and correct answers are not associated with confidence; however, the models do show more confidence on Realistic problems, and less on Nonsense ones. (Effects are calculated from a mixed-effects regression predicting the difference in log-probability between the top and second-highest answer, z-scored within each model, and controlling for all other significant predictors. Errorbars are parametric 95% CIs. Note that human RT is calculated across all human subjects for the Wason task, not just slow subjects).

We summarize these results in Fig. 7. We measure model confidence as the difference in prior-corrected log-probability between the top answer and the second highest—if the model is almost undecided between several answers, this confidence measure will be low, while if the model is placing almost all its probability mass on a single answer, the confidence measure will be high. In mixed-effects regressions predicting model confidence from task variables and average human RTs on the same problem, we find several interesting effects. First, LMs tend to be more confident on correct answers (i.e. they are somewhat calibrated). Task variables also affect confidence; models are generally less confident when the conclusion violates beliefs, and more confident for the realistic rules on the Wason task. Furthermore, even when controlling for task variables and accuracy, there is a statistically significant negative association with human response times on the NLI and syllogisms tasks (respectively t(655)=−3.39, P<0.001; and t(353)=−2.03, P<0.05; Supplementary Material C.4)—that is, models tend to show more confidence on problems where humans likewise respond more rapidly. We visualize this relationship in Fig. S34.

### Analyzing components of the Wason responses

**Fig. 8.**
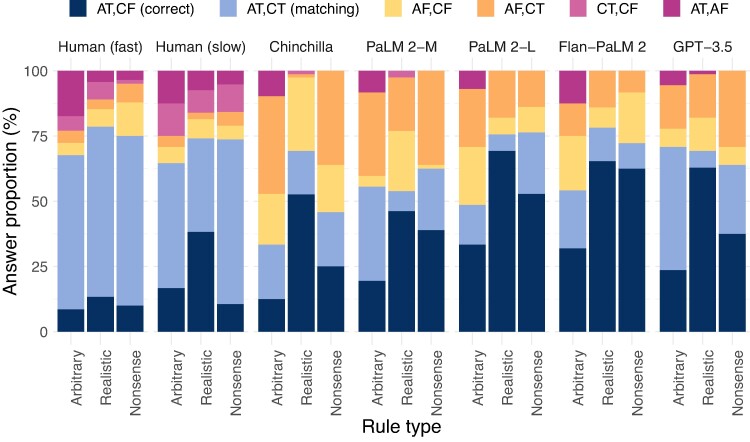
Answer patterns for the Wason tasks, broken down into the pairings of individual cards that each participant chose (AT = Antecedent True, CF = Consequent False, etc.). Behavior is not random, even when performance is near chance. As above, humans do not usually choose the correct answer (AT, CF; dark blue); instead, humans more frequently exhibit the matching bias (AT, CT; light blue). Humans also show other errors, e.g. surprisingly often choosing two cards corresponding to a single rule component (pink). Language models answer correctly more often than humans, but intriguingly choose options with the antecedent false and a consequent card (yellow/orange) more frequently.

Because each answer to the Wason problems involves selecting a pair of cards, we further analyzed the individual cards chosen. In each problem, two cards respectively match and violate the antecedent, and similarly for the consequent. The correct answer is to choose the card for which the Antecedent is True (AT), and the card for which the Consequent is False (CF). In Fig. 8, we examine human and model choices; we quantify these analyses with a multinomial logistic regression in Supplementary Material C.3.2.

Even when performance is close to chance, behavior is generally not random. As in prior work, humans do not consistently choose the correct answer (AT, CF). Instead, humans tend to exhibit a matching bias; that is, they tend to choose the two cards that match each component of the rule (AT, CT). However, in the Realistic condition, slow humans answer correctly somewhat more often. Humans also exhibit errors besides the matching bias, including an increased rate of choosing the two cards corresponding to a single component of the rule—either both antecedent cards, or both consequent cards. LMs tend to give more correct responses than humans, and to show facilitation in the realistic rules compared to arbitrary ones. Relative to humans, LMs show fewer matching errors and fewer errors of choosing two cards from the same rule component, but more errors of choosing the antecedent false options. These differences in error patterns may indicate differences between the response processes engaged by the models and humans. (Note, however, that while model accuracies do not change too substantially with alternate scoring methods, the particular errors the models make are somewhat sensitive to scoring method—without the DC-PMI correction the model errors more closely approximate the human ones in some cases; Supplementary Material B.2.3).

## Discussion

Humans are imperfect reasoners. We reason most effectively about situations that are consistent with our understanding of the world and often struggle to reason in situations that either violate this understanding or are abstract and disconnected from the real world. Our experiments show that language models mirror these patterns of behavior. Language models also perform imperfectly on logical reasoning tasks and more often fail in situations where humans fail—when stimuli become too abstract or conflict with prior expectations.

Beyond these simple parallels in accuracy across different conditions and tasks, we also observed more subtle parallels in language model confidence. Language model confidence tends to be higher for correct answers, and for cases where prior expectations about the content are consistent with the logical structure. Even when controlling for these effects, language model confidence is related to human response times. Thus, language models reflect similarities to human content effects on reasoning at multiple levels. Furthermore, these core results are generally robust across different language models with different training and tuning paradigms, different prompts, etc., suggesting that they are a fairly general phenomenon of predictive models that learn from human-generated text. Importantly, however, humans and language models also differ in interesting ways; particularly in their performance and error patterns on the more complex Wason card selection task.

###  

####  

##### Prior research on language model reasoning.

Since Brown et al. (6) showed that large language models could perform moderately well on some reasoning tasks, there has been a growing interest in language model reasoning (57). Typical methods focus on prompting for sequential reasoning (7, 8, 52), altering task framing (58, 59).

In response, some researchers have questioned whether these language model abilities qualify as “reasoning”. The fact that language models sometimes rely on “simple heuristics” (11), or reason more accurately about frequently occurring numbers (12), have been cited to “rais[e] questions on the extent to which these models are *actually reasoning*” (ibid, emphasis ours). The implicit assumption in these critiques is that reasoning should be a purely algebraic, syntactic computation over symbols from which “all meaning had been purged” ((2); cf. (26)). In this work, we emphasize how *both* humans and language models rely on content when answering reasoning problems—using simple heuristics in some contexts, and answering more accurately about frequently occurring situations (23, 60). Thus, abstract reasoning may be a graded, content-sensitive capacity in both humans and models.

##### Dual systems?

The idea that humans possess dual reasoning systems—an implicit, intuitive system “system 1”, and a distinct explicit reasoning “system 2”—was motivated in large part by belief bias and Wason task effects (61–63). The dual system idea has more recently become popular (64), including in machine learning. Many works claim that current ML (including large language models) behave like system 1, and that we need to augment this with a classically symbolic process to get system 2 behaviour (e.g. (65)). These calls to action generally advocate for an explicit duality, with a neural network based system providing the system 1 and an explicitly symbolic or otherwise more structured architecture serving as system 2.

Our results show that a single system—a large transformer language model—can mirror this dual behavior in humans, demonstrating both biased and consistent reasoning without an explicit secondary symbolic “system 2”. In direct analogy to “fast” vs. “slow” responses (64) in humans, we compare model behavior on the complex Wason task with and without a chain-of-thought prompt, and find that the chain-of-thought prompt can move a strong model from strong content-biases to achieving fairly high accuracy across Arbitrary and Nonsense conditions. These findings integrate with prior works showing that language models can be prompted to exhibit “slow” or “system-2-like” sequential reasoning, and thereby improve their performance in domains like mathematics (7, 8, 52).

These results show that dual-system-like behaviors need not rely on an explicitly symbolic and separate system 2—they can instead arise from implicit systems that use context to arbitrate between conflicting responses (such as intuitive answers vs. those supported by step-by-step outputs). This finding does not deny the possibility that humans may possess cognitive control mechanisms not currently found in LMs (66, 67); their possible integration into LMs is an exciting direction for future research.

##### Towards a normative account of content effects?

Various accounts of human cognitive biases frame them as “normative” by some objective. Some works explain biases through the application of processes—such as information gathering or pragmatics—that are broadly rational under a different model of the world (e.g. (43, 63)). Others interpret them as rational adaptations to reasoning under constraints such as limited memory or time (e.g. (68, 69))—where content effects actually support fast and effective reasoning in commonly encountered tasks (23, 60). However, most prior high-level explanations of content effects have focused on only a single task, such as explaining *only* the Wason task content effects with appeals to evolved social-reasoning mechanisms (21). Our results show that these effects can emerge from simply training a large LM to imitate sufficiently large quantities of language produced by human culture, without explicitly incorporating any human-specific internal mechanisms.

This observation suggests two potential origins for these content effects. First, content effects could be directly imitated from the humans that generated the LM training data. Under this hypothesis, poor logical inferences about nonsense or belief-violating premises come from *copying* the incorrect inference patterns humans use. Since humans also learn substantially from imitation and the cultures in which we are immersed, it is plausible that both humans and LMs could acquire some of these reasoning patterns by imitation.

The other possibility is that, like humans, an LM’s experience reflects real-world semantics, and thus LMs and humans both converge on these content biases that reflect this semantic content for task-oriented reasons—because it helps humans to draw more accurate inferences in the situations they more frequently encounter (which are mostly familiar and believable), and helps language models to more accurately predict the (mostly believable) text that they encounter.

In either case, humans and models acquire surprisingly similar patterns of behavior (in many, though not all cases), even though there are important differences between these systems. What could give rise to these similarities? A promising direction for future enquiry would be to causally manipulate features of LM training objectives and data, to explore which features contribute to the emergence of content biases—and which features might yield behavior even more similar to that of humans. In such work, it would be useful to experimentally manipulate the training dataset scale, and evaluate whether these biases still emerge from models trained on a more human-like quantity of language (cf. (70)). If so, these investigations could offer insights into the potential origins of human patterns of reasoning. More generally, such investigations would offer insights into the data we should use to train language models.

##### Why might model response patterns differ from human ones?

The LM response patterns do not perfectly match all aspects of the human data. For example, on the Wason task large models generally outperform the humans, and the error patterns on the Wason tasks are somewhat different than those observed in humans (although human error patterns also vary across populations; (48, 49)). Similarly, not all models show the significant interaction between believability and validity on the syllogism tasks that humans do (14), although it is present in most models (and the human interaction similarly may not appear in all cases; (44)). Various factors could contribute to differences between model and human behaviors.

First, while we attempted to align our human and model evaluation as closely as possible (cf. (59)), it is difficult to do so perfectly. In some cases, such as the Wason task, differences in response form are unavoidable—humans selected cards individually by clicking them, before clicking continue, while models had to jointly output both answers in text, without a chance to revise their answer before continuing. Moreover, it is difficult to know how to prompt a language model in order to evaluate a particular task. Language model training blends many tasks into a homogeneous soup, which makes controlling the model difficult. For example, presenting task instructions might not always lead to better performance (cf. (71)). Thus, while we tried to match instructions between humans and models, it is possible that idiosyncratic details of our task framing may have caused the model to infer the task incorrectly. To minimize this risk, we tried varying these details, and generally observed similar overall effects. However, our chain-of-thought results (Supplementary Material B.3) show that large models *can* in some cases shift their performance substantially when prompted appropriately with a clear demonstration of a reasoning process. It is possible that the appropriate prompt conditions would thus yield more human-like behavior.

More fundamentally, however, LMs do not directly experience the situations to which language refers (72); grounded experience presumably underpins some human beliefs and reasoning. Indeed, humans sometimes use physical processes like gesture to support reasoning (73). Finally, language models experience language passively, while humans experience language as an active, conventional system for social communication (e.g. (74); active participation may be key to understanding meaning as humans do (28, 75). Some differences between language models and humans may therefore stem from differences between the rich, grounded, interactive experience of humans, and the impoverished experience of the models.

##### How can we achieve more abstract, context-independent reasoning?

If language models exhibit some of the same reasoning biases as humans, could some of the factors that reduce content dependency in human reasoning be applied to make these models less content-dependent? One method to do so is through prompting, which can indeed help in some cases (Supplementary Material B3). However, we suspect achieving fully consistent reasoning across all tasks would likely require altering the models training. For humans, formal education is associated with an improved ability to reason logically and consistently (48, 49, 76, 77). However, causal evidence is scarce, because years of education are difficult to experimentally manipulate; thus the association may be partly due to selection effects, e.g. continuing in formal education might be more likely in individuals with stronger prior abilities. Nevertheless, the association with formal education raises an intriguing question: could language models learn to reason more reliably with targeted formal education?

Several recent results suggest that this may indeed be a promising direction. Pretraining on synthetic logical reasoning tasks can improve model performance on reasoning and mathematics problems (78). In some cases language models can either be prompted or can learn to verify, correct, or debias their own outputs (79). Finally, language model reasoning can be bootstrapped through iterated fine-tuning on successful instances (80). These results suggest the possibility that a model trained with instructions to perform logical reasoning, and to check and correct the results of its work, might move closer to the logical reasoning capabilities of formally educated humans. Perhaps logical reasoning is a graded competency that is supported by a range of different environmental and educational factors (28), rather than a core ability that must be built in to an intelligent system.

##### Limitations

In addition to the limitations noted above—such as the challenges of perfectly aligning comparisons between humans and language models—there are several other limitations to our work. First, we only considered a handful of tasks in this work—it would be useful to characterize human and LM content effects across a broader range of tasks in order to fully understand their similarities and differences. Second, our human participants exhibited relatively low performance on the Wason task. However, as noted above, there are well-known individual differences in these effects that are associated with factors like depth of mathematical education. We were unfortunately unable to examine these effects in our data, but in future work it would be interesting to explicitly explore how educational factors affect performance on our datasets.

Another limitation is that the language models are trained on much greater quantities of language data than any human, which makes it hard to draw strong conclusions about whether these effects would emerge at a more human-like data scale (70). Furthermore, while our experiments suggest that content effects in reasoning can emerge from predictive learning on naturalistic data, they do not ascertain precisely which aspects of the large language model training datasets contribute to this learning. Other research has used controlled training data distributions to systematically investigate the origin of language model capabilities (81, 82); it would be an interesting future direction to apply analogous methods to investigate the origin of content effects, and whether they would still emerge for a model trained on a more human-like quantity of language.

## Materials and methods

###  

####  

##### Creating datasets

While our tasks have been extensively studied in cognitive science, the stimuli used previously are often online in articles and course materials, and thus may be present in LM training data, which could compromise results (e.g. (83)). To reduce these concerns, we generate new datasets, by following the design approaches used in prior work. We briefly outline this process here; see Supplementary Material A.1 for full details.

For each of our tasks, we generate multiple versions of the task stimuli. Throughout, the logical structure of the stimuli remains fixed, we simply manipulate the entities over which this logic operates (Fig. 1). We generate propositions that are: *Consistent* with human beliefs and knowledge; *Violate* beliefs by inverting the consistent statements; and *Nonsense* tasks about nonsense pseudowords about which the model should not have strong beliefs (e.g. kleegs are smaller than feps).

For the Wason tasks, we slightly alter our approach to fit the different character of the tasks. We generate questions with: *Realistic* rules involving plausible relationships; *Arbitrary* rules; and *Nonsense* rules relating nonsense words (“if the cards have more bem, then they have less stope”). For the Wason task, this alters the inferences required; for the other conditions matching each card to the antecedent or consequent is nontrivial (e.g. realizing that “shoes” is plural), but we cannot match these challenges with Nonsense words that have no prior associations. Thus, we use propositions about having more/less of a nonsense attribute, which makes the inferences more direct than other conditions (although LMs perform similarly on the basic inferences across conditions; Fig. S24.

In total, our NLI dataset contains 78 questions per condition, our syllogisms dataset has 48 questions per Consistent/Violate condition (24 each valid/invalid), and our Wason dataset contains 72 questions per Realistic/Arbitrary condition. For the latter datasets, the Nonsense condition contains more stimuli, as we created Nonsense stimuli matching stimuli from both Consistent and Violate conditions.

In Supplementary Material B.1, we validate the semantic content of our datasets, by showing that participants find the propositions and rules from our Consistent and Realistic stimuli much more plausible than those from other conditions, and that similarly an LM assigns higher probability to the more familiar conditions.

We attempted to create these datasets in a way that could be presented to the humans and language models in precisely the same manner (e.g. prefacing the problems with the same instructions for both the humans and the models).^a^

##### Models & evaluation

We evaluate several different families of language models (see Supplementary Material A.4 for a detailed comparison). First, we evaluate several base LMs that are trained only on language modeling: including Chinchilla (37) a large model (with 70 billion parameters) trained on causal language modeling, and PaLM 2-M and -L (36), which are trained on a mixture of language modeling and infilling objectives (84). We also evaluate two instruction-tuned models: Flan-PaLM 2 (an instruction-tuned version of Palm 2-L), and GPT-3.5-turbo-instruct (38), which we generally refer to as GPT-3.5 for brevity. We observe broadly similar content effects across all types of models, suggesting that these effects are not too strongly driven by a particular training objective, or standard fine-tuning.

For each task, we present the model with brief instructions that approximate the relevant human instructions. We then present the question, which ends with “Answer:” and assess the model by evaluating the likelihood of continuing this prompt with each of a set of possible answers. We apply the DC-PMI correction proposed by Holtzman et al. (85)—i.e. we measure the change in likelihood of each answer in the context of the question relative to a baseline context, and choosing the answer that has the largest increase in likelihood in context. This scoring approach is intended to reduce the possibility that the model would simply phrase the answer differently than the available choices; for example, answering “this is not a valid argument” rather than “this argument is invalid”. This approach can also be interpreted as correcting for the prior over utterances. For the NLI task, however, the direct answer format means that the DC-PMI correction would therefore control for the very bias we are trying to measure. Thus, for the NLI task we simply choose the answer that receives the maximum likelihood among the set of possible answers. We also report syllogism and Wason results with maximum likelihood scoring in Supplementary Material B.2.2; while overall accuracy changes (usually decreases, but with some exceptions), the direction of content effects is generally preserved under alternative scoring methods.

##### Human experiments

All human experimental procedures were approved by the DeepMind independent ethical review committee; all participants provided informed consent to participate. The human experiments were conducted in 2023 using an online crowd-sourcing platform, with participants from the United Kingdom who spoke English as a first language, and who had over a 95% approval rate. We did not further restrict participation. We offered pay of £2.50 for our task. Our intent was to pay at a rate exceeding £15/h, and we exceeded this target, as most participants completed the task in less than 10 min.

Participants were first presented with a consent form detailing the experiment and their ability to withdraw. If they consented, they then proceeded to the instructions. They were then presented with one question from each of our three tasks, one at a time in randomized order, with randomized conditions. Presenting only a single item per task prevents within-task contamination effects—particularly for tasks like the Wason, which essentially involve identical reasoning each time. Finally, participants were asked to rate the believability of three items (a Wason rule and concluding proposition from the syllogisms and NLI tasks) on a continuous scale from 0 to 100 (with 50% indicated as neither agree nor disagree). The participants 5 min to answer each question. See Supplementary Material A.3 for further details.

We first collected a dataset from 625 participants. After observing the low Wason task accuracy, we collected an additional dataset from 360 participants in which we offered an additional performance bonus of £0.50 for answering the Wason correctly, to motivate subjects. In this replication, we collected data only on the Realistic and Abstract Wason conditions. In our main analyses, we collapse across these two subsets, but we present the results for each experiment separately in Fig. S26.

##### Statistical analyses

Main analyses use mixed-effects logistic regressions with task condition variables as predictors, and controlling for random effects of items, and, where applicable, models. Full specifications and results are provided in Supplementary Material C.

## Supplementary Material

pgae233_Supplementary_Data

## Data Availability

The human & LM data from all main experiments are available at https://console.cloud.google.com/storage/browser/gdm_lm_content_effects.

## Notes

a N.B. we adapted some problem formats compared to an earlier preprint that did not evaluate humans; see Supplementary Material A.1.4.

## Preprints

This manuscript was posted as a preprint: https://arxiv.org/abs/2207.07051.
