# Supplementary material for: Language models, like humans, show content effects on reasoning tasks
Source: PNAS Nexus. 2024 Jul 16;3(7):pgae233. doi: 10.1093/pnasnexus/pgae233 (PMC11250216; doi:10.1093/pnasnexus/pgae233)
Supplement: pgae233_Supplementary_Data [file pgae233_supplementary_data.pdf]

In SI A we provide more details of the methods and datasets, in SI B we provide supplemental analyses, and in SI C we provide full results of statistical models for the main results.

A Supplemental methods

A.1 Datasets

If seas are bigger than puddles, then puddles are...
Choice: smaller than seas
Choice: bigger than seas
Answer: **smaller than seas**

(a) Natural language inference.

Argument:
All diamonds are gems.
Some gems are transparent things.
Conclusion: Some diamonds are transparent things.
Answer: **This argument is invalid.**

(b) Syllogism validity.

A parent needs to check that their children are following the rules. The following cards represent the children; they each have a current activity on one side and a homework completion on their other side. The rule is that if the children are playing games then they must have finished their homework. Which of the cards would you need to flip over to help them to ensure that the rule is being followed?
card: playing video games
card: eating dinner
card: homework is not done
card: homework is done
Answer: **You need to flip over the ones showing "playing video games" and "homework is not done"**

(c) Wason selection task.

Figure S1: Examples of the three logical reasoning tasks we evaluate, as they were presented to the models: (a) simple single-step natural language inferences, (b) assessing the validity of logical syllogisms, and (c) the Wason selection task. In each case, the model must choose the answer (blue and bold) from a set of possible answer choices.

As noted in the main text, we generated new datasets for each task to avoid problems with training data contamination. In this section we present further details of dataset generation.

A.1.1 NLI task generation

In the absence of existing cognitive literature on generating belief-aligned stimuli for this task, we used a larger language model (Gopher, 280B parameters, from 13) to generate 100 comparison statements automatically, by prompting it with 6 comparisons that are true in the real world. The exact prompt used was:

```
The following are 100 examples of comparisons:

1. mountains are bigger than hills
```

```
2. adults are bigger than children

3. grandparents are older than babies

4. volcanoes are more dangerous than cities

5. cats are softer than lizards
```

We prompted the LLM multiple times, until we had generated 100 comparisons that fulfilled the desired criteria. The prompt completions were generated using nucleus sampling (121) with a probability mass of 0.8 and a temperature of 1. We filtered out comparisons that were not of the form "[entity] is/are [comparison] than [other entity]". We then filtered these comparisons manually to remove false and subjective ones, so the comparisons all respect real-world facts. An example of the generated comparisons includes "puddles are smaller than seas".

We generated a natural inference task derived from these comparison sentences as follows. We began with the *consistent* version, by taking the the raw output from the LM, "puddles are smaller than seas" as the hypothesis and formulating a premise "seas are bigger than puddles" such that the generated hypothesis is logically valid. We then combine the premise and hypothesis into a prompt and continuations. For example:

```
If seas are bigger than puddles, then puddles are
A. smaller than seas
B. bigger than seas
```

where the logically correct (A) response matches real-world beliefs (that 'puddles are smaller than seas'). Similarly, we can also generate a *violate* version of the task where the logical response violates these beliefs. For example,

```
If seas are smaller than puddles, then puddles are
A. smaller than seas
B. bigger than seas
```

here the correct answer, (B), violates the LM's prior beliefs. Finally, to generate a *nonsense* version of the task, we simply replace the nouns ('seas' and 'puddles') with nonsense words. For example:

```
If vuffs are smaller than feps, then feps are
```

Argument:<br>No flowers are animals.<br>All reptiles are animals.<br>Conclusion: No flowers are reptiles.

(a) Valid, consistent

Argument:<br>No flowers are animals.<br>All reptiles are flowers.<br>Conclusion: No reptiles are animals.

(b) Valid, violate

Argument:<br>No flowers are reptiles.<br>All reptiles are animals.<br>Conclusion: No flowers are animals.

(c) Invalid, consistent

Argument:<br>No flowers are animals.<br>All flowers are reptiles.<br>Conclusion: No reptiles are animals.

(d) Invalid, violate

Figure S2: Example syllogism cluster, showing 2×2 design of valid (top row), invalid (bottom row), and consistent (left column) and violate (right column) arguments.

```
A. smaller than vuffs
B. bigger than vuffs
```

Here the logical conclusion is B. For each of these task variations, we evaluate the log probability the language model places on the two options and choose higher likelihood one as its prediction.

A.1.2 Syllogisms data generation

We generated a new set of problems for syllogistic reasoning. Following the approach of Evans et al. (20), in which the syllogisms were written based on the researchers' intuitions of believability, we hand-authored these problems based on beliefs that seemed plausible to the authors. See Fig. S1b for an example problem. We built the dataset from clusters of 4 arguments that use the same three entities, in a 2×2 combination of valid/invalid, and belief-consistent/violate. For example, in Fig. S2 we present a full cluster of arguments about reptiles, animals, and flowers.

By creating the arguments in this way, we ensure that the low-level properties (such as the particular entities referred to in an argument) are approximately balanced across the relevant conditions. In total there are twelve clusters. We avoided using the particular negative form ("some X are not Y") to avoid substantial negation, which complicates behavior both for language models and humans (cf. 122, 123). We then sampled an identical set of nonsense

```
Some librarians are happy people
All happy people are healthy people
Conclusion: Some librarians are healthy people

All guns are weapons
All weapons are dangerous things
Conclusion: All guns are dangerous things

Some electronics are computers
All computers are expensive things
Conclusion: Some electronics are expensive things

All trees are plants
Some trees are tall things
Conclusion: Some plants are tall things

No flowers are animals
All reptiles are animals
Conclusion: No flowers are reptiles

All diamonds are gems
Some diamonds are transparent things
Conclusion: Some gems are transparent things

All dragons are mythical creatures
No mythical creatures are things that exist
Conclusion: No dragons are things that exist

Some politicians are dishonest people
All dishonest people are people who lie
Conclusion: Some politicians are people who lie

All whales are mammals
Some whales are big things
Conclusion: Some mammals are big things

All vegetables are foods
Some vegetables are healthy things
Conclusion: Some foods are healthy things

All famous actors are wealthy people
Some famous actors are old people
Conclusion: Some old people are wealthy people

All vehicles are things that move
No buildings are things that move
Conclusion: No buildings are vehicles
```

Figure S3: One argument (valid, consistent) from each of the 12 argument clusters we used for the syllogisms tasks, showing the entities and argument forms covered.

arguments by simply replacing the entities in realistic arguments with nonsense words.

We present the arguments to the model, and give a forced choice between "The argument is valid." or "The argument is invalid." Where example shots are used, they are sampled from distinct clusters, and are separated by a blank line. We also tried some minor variations in preliminary experiments (such as changing the prompt or prefixing the conclusion with "Therefore:" or omitting the prefix before the conclusion), but observed qualitatively similar results so we omit them here.

A.1.3 Wason data generation

As above, we generated a new dataset of Wason problems to avoid potential for dataset contamination (see Fig. S1c for an example). The final response in a Wason task does not involve a declarative statement (unlike completing a comparison as in NLI), so answers do not directly 'violate' beliefs. Rather, in the cognitive science literature, the key factor affecting human performance is whether the entities are 'realistic' and follow 'realistic' rules (such as people following social norms) or consist of arbitrary relationships between abstract entities such as letters and numbers. We therefore study the effect of realistic and arbitrary scenarios in the

language models.

We created 12 realistic rules and 12 arbitrary rules. Each rule appears with four instances, respectively matching and violating the antecedent and consequent. Each realistic rule is augmented with one sentence of context for the rule, and the cards are explained to represent the entities in the context. The model is presented with the context, the rule, and is asked which of the following instances it needs to flip over, then the instances. The model is then given a forced choice between sentences of the form "You need to flip over the ones showing "X" and "Y"." for all subsets of two items from the instances. There are two choices offered for each pair, in both of the possible orders, to eliminate possible biases if the model prefers one ordering or another. (Recall that the model scores each answer independently; it does not see all answers at once.)

See Figs. S4 and S5 for the realistic and arbitrary rules and instances used — but note that problems were presented to the model with more context and structure, see Fig. S1c for an example. We demonstrate in SI B.4 that the difficulty of basic inferences about the propositions involved in each rule type is similar across conditions.

We also created 12 rules using nonsense words. Incorporating nonsense words is less straightforward in the Wason case than in the other tasks, as the model needs to be able to reason about whether instances match the antecedent and consequent of the rule. We therefore use nonsense rules of the form "If the cards have less gluff, then they have more caft" with instances being more/less gluff/caft. The more/less framing makes the instances roughly the same length regardless of rule type, and avoids using negation which might confound results (122).

Finally, we created two types of control rules based on the realistic rules, which we present here. First, we created shuffled realistic rules by combining the antecedents and consequents of different realistic rules, while ensuring that there is no obvious rationale for the rule. For

example, one shuffled-realistic rule is "If they are doctors, then they must have a parachute."

We then created violate-realistic rules by taking each realistic rule and reversing its consequent. For example, the realistic rule "If the clients are skydiving, then they must have a parachute" is transformed to the violate rule "If the clients are skydiving, then they must have a wetsuit", but "parachute" is still included among the cards. The violate condition is designed to make the rule especially implausible in context of the examples (viz. requiring the item that is *not* a parachute to skydive), while the rule in the shuffled condition is somewhat more arbitrary/belief neutral.

To rule out a possible specific effect of cards (which were used in the original tasks) we also sampled versions of each problem with sheets of paper or coins, but results are similar so we collapse across these conditions in the main analyses.

A.1.4 Differences from an earlier preprint of this paper:

Readers of an earlier preprint of this paper (`https://arxiv.org/abs/2207.07051v1`) may notice some differences in task format and performance of Chinchilla, especially on the NLI task. These differences are due to our attempts to adapt the tasks in order to present them to human participants. In order to align comparisons between humans and the language models (cf. 73), we then ported the human-oriented changes back into the format used for language model evaluation.

For example, in the original paper we did not show the model the two possible choices for the NLI task; we simply evaluated the model's likelihood of each continuation. However, because we presented the tasks multiple choice to the humans in multiple choice format, we showed them the two possible answers. Thus, in the current version of the paper we also included the two answer choices in the prompt when evaluating the language models, followed by "Answer:", and only then evaluate the model (see Fig. S1a). Likewise, in the original version

```
An airline worker in Chicago needs to check passenger documents. The rule is that if the passengers are traveling outside the
↪  US then they must have showed a passport.
Buenos Aires / San Francisco / passport / drivers license

A chef needs to check the ingredients for dinner. The rule is that if the ingredients are meat then they must not be expired.
beef / flour / expires tomorrow / expired yesterday

A lawyer for the Innocence Project needs to examine convictions. The rule is that if the people are in prison then they must
↪  be guilty.
imprisoned / free / committed murder / did not commit a crime

A medical inspector needs to check hospital worker qualifications. The rule is that if the workers work as a doctor then they
↪  must have received an MD.
surgeon / janitor / received an MD / received a GED

A museum curator is examining the collection. The rule is that if the artworks are in the museum then they must be genuine.
displayed in the museum / not in the museum / genuine / forgery

An adventure trip organizer needs to ensure their clients have the appropriate gear. The rule is that if the clients are going
↪  skydiving then they must have a parachute.
skydiving / mountain biking / parachute / wetsuit

A parent needs to check that their children are following the rules. The rule is that if the children are playing games then
↪  they must have finished their homework.
playing video games / eating dinner / homework is done / homework is not done

A priest needs to check if people are ready for marriage. The rule is that if the people are engaged then they must be adults.
engaged / single / 25 years old / 7 years old

A traffic enforcement officer needs to check that people are following the law. The rule is that if the people in vehicles are
↪  driving then they must have a driver license.
driver / passenger / has a license / does not have a license

A gardener needs to take care of their plants. The rule is that if the plants are flowers then they must be fertilized.
rose / oak / fertilized / not fertilized

A farmer is getting equipment ready for the day. The rule is that if the pieces of equipment have an engine then they must
↪  have fuel.
tractor / shovel / has gasoline / does not have gasoline

A person is cleaning out and organizing his closet. The rule is that if the clothes are going to the thrift store then they
↪  must be old.
thrift store / keep / worn out / brand new

An employer needs to check that their business is following health regulations. The rule is that if the employees are working
↪  then they must not be sick.
working / on vacation / healthy / has a cold
```

Figure S4: Realistic Wason rules and instances used.

```
The rule is that if the cards have a plural word then they must have a positive emotion.
crises / dog / happiness / anxiety

The rule is that if the cards have a soft texture then they must have a polygon.
soft / rough / hexagon / circle

The rule is that if the cards have a French word then they must have a positive number.
chapeau / sombrero / 4 / -1

The rule is that if the cards have a prime number then they must have a secondary color.
11 / 12 / purple / red

The rule is that if the cards have a European country then they must have something hot.
Germany / Brazil / furnace / ice cube

The rule is that if the cards have the name of a famous book then they must have the name of an elementary particle.
Moby Dick / Citizen Kane / neutrino / atom

The rule is that if the cards have a type of plant then they must have the name of a philosopher.
cactus / horse / Socrates / Napoleon

The rule is that if the cards have the name of a web browser then they must have a type of pants.
Internet Explorer / Microsoft Word / jeans / sweatshirt

The rule is that if the cards have a beverage containing caffeine then they must have a material that conducts electricity.
coffee / orange juice / copper / wood

The rule is that if the cards have something electronic then they must have a hairy animal.
flashlight / crescent wrench / bear / swan

The rule is that if the cards have a verb then they must have a Fibonacci number.
walking / slowly / 13 / 4

The rule is that if the cards have a text file extension then they must have a time in the morning.
.txt / .exe / 11:00 AM / 8:00 PM
```

Figure S5: Arbitrary Wason rules and instances used.

of the paper we did not provide instructions before the tasks; in this version we attempted to match the relevant portions of the human instructions.

These changes mean that the results in the current version of the paper cannot be directly compared to the results in the earlier version.

A.2 Evaluation

DC-PMI correction: We use the DC-PMI correction (120) for the syllogisms and Wason tasks; i.e., we choose an answer from the set of possible answers ($\mathcal{A}$) as follows:

$$\text{argmax}_{a \in \mathcal{A}} \; p(a \,|\, \text{question}) - p(a \,|\, \text{baseline prompt})$$

Where the baseline prompt is the task instruction prompt, followed by "Answer:" and $p(x \,|\, y)$ denotes the model's evaluated likelihood of continuation x after prompt y.

Instruction prompt: We prefixed each question with a two-part instruction prompt that attempted to match the human generic and task-specific instructions (see below). We began

each of these prompts with the performance relevant generic instructions that preceded our human experiment:

```
In this task, you will have to answer a series of questions. You will have to choose the best answer to complete a sentence,
↪  paragraph, or question. Please answer them to the best of your ability.\n\n
```

After two linebreaks, a task-specific instruction was appended:

NLI:

```
Please choose the best completion for the following sentence:\n
```

Syllogisms:

```
Please assume that the first two sentences in the argument are true. Determine whether the argument is valid, that is, whether
↪  the conclusion follows from the first two sentences:\n'
```

Wason:

```
Please answer the following question carefully:\n
```

Finally, the question was appended to this prompt.

A.3 Human experiments

The exact text seen by the participants before each question was as follows:

```
NLI_DEFAULT_PREFACE = (
    "Please choose the best completion for the following sentence:")
SYLLOGISMS_DEFAULT_PREFACE = (
    "Please assume that the first two sentences in the argument are true. "
    "Determine whether the argument is valid, that is, whether the
       conclusion "
    "follows from the first two sentences:")
WASON_DEFAULT_PREFACE = (
    "Please answer the following question carefully:"
)
WASON_BONUS_PREFACE = (
    "Please answer the following question carefully; <font color='#bb0044'>
       we "
    "will pay you an additional performance bonus of 0.5 GBP if you answer
       "
    "this question correctly</font>:"
)
PRIOR_AGREEMENT_PREFACE = (
    "Please rate how much you agree with the following statement, on a
       scale "
    "from 0% (disagree completely) to 50% (neither agree nor disagree) to
       100% "
    "(agree completely)."
)
WASON_BELIEVABLE_PREFACE = (
```

```
    "Please rate how believable the following rule is, on a scale from 0% "
    "(completely unbelievable) to 50% (neither believable nor unbelievable)
        to "
    "100% (completely believable)."
)
```

Due to infrastructure restrictions in the framework used to create the human tasks, we assigned participants to conditions and items randomly rather than with precise balancing. Furthermore, a few participants timed out on some questions, and there were a handful of instances of data not saving properly due to server issues. Thus, the exact number of participants for which we have data varies slightly from task to task and item to item.

A.4 Language Models

In Table S1, we provide a detailed comparison of the language models that we evaluated. Note that some details are not publicly available; thus, the information is unfortunately incomplete.

Name	Citation	Architecture	Training Data	Pretraining Objective	Instruction tuned?	# Parameters
Chinchilla	Hoffmann et al. (45)	Decoder-only + RMSNorm + Relative Positions.	MassiveText (13), for 1.4 trillion tokens	Causal language modeling	No	70 billion
PaLM 2-M	Anil et al. (47)	*	*	Mixed (119)	No	*
PaLM 2-L	Anil et al. (47)	*	*	Mixed (119)	No	*
Flan-PaLM 2	Anil et al. (47)	Same as PaLM 2-L			Yes	Same as Palm 2-L
GPT-3.5-turbo-instruct	(46)	*	*	*	Yes	*

Table S1: Comparing attributes of the different language models we evaluated in this work. (* denotes information that has not been publicly disclosed.)

evaluate several different families of language models. First, we evaluate several base LMs that are trained only on language modeling: including Chinchilla (45) a large model (with 70 billion parameters) trained on causal language modeling, and PaLM 2-M and -L (47), which are trained on a mixture of language modeling and infilling objectives. We also evaluate two instruction-tuned models: Flan-PaLM 2, which is an instruction-tuned version of Palm 2-L, and

GPT-3.5-turbo-instruct (46), which we generally refer to as GPT-3.5 for brevity. We note that Flan-PaLM 2 is fine-tuned on NLI tasks (cf. 124), but with the more standard NLP formulation; however, since even the base PaLM 2-L model performs extremely well at the NLI tasks we do not believe this substantially influences the results.

B Supplemental analyses

B.1 Believability of the propositions and rules

In order to assess the validity of our new datasets, we collected believability ratings from each human subject, after they had completed the three tasks tasks, on one stimulus from each task type (not the version they had seen). Specifically, we asked the participants how believable a Wason rule was, and how much they agreed or disagreed with a proposition. In Fig. S6 we show that participants found Consistent and Realistic stimuli much more believable than those in other conditions.

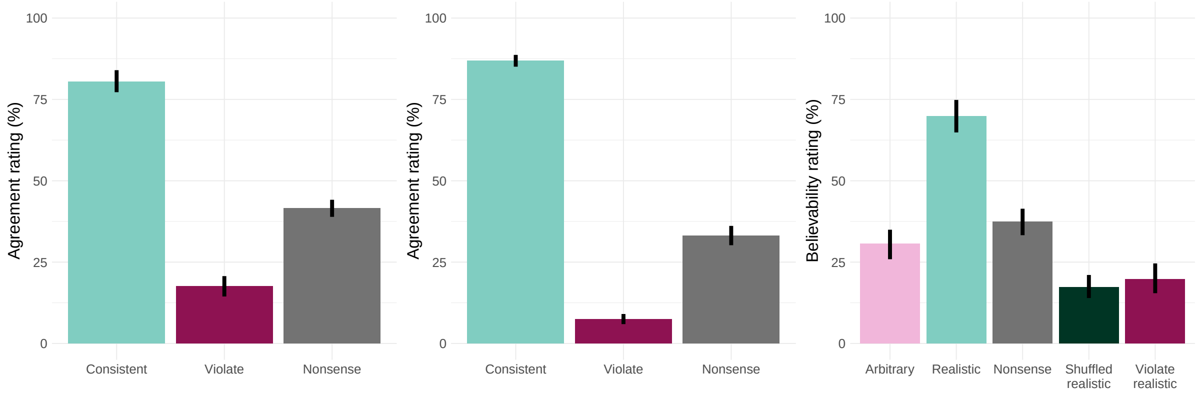

(a) NLI (b) Syllogisms (c) Wason

Figure S6: Our datasets align with human beliefs. When participants were asked how much they believed propositions or rules from our three tasks (a-c), they rated the Consistent or Realistic conditions as much more believable than the Violate ones, with Nonsense in between.

Chinchilla: We similarly evaluated the likelihood (log-probability) of the key propositions,

conclusions, and rules from each task according to the Chinchilla language model. This evaluation is an attempt at a manipulation check that the model priors over the stimuli accord with the human ones; however, note that it is difficult to interpret the raw likelihood comparisons as direct measures of believability, because sequences composed of more tokens will tend to have lower likelihood. For this reason, the Nonsense sentences tend to score much lower than the other conditions (because the nonsense words are composed of many tokens in an unlikely sequence, while the real words we use are common and thus generally are a single token). However, we do find that the model tends to assign higher log-probabilities to stimuli from the Consistent or Realistic conditions than the others, consistent with our argument that these statements fit the model's priors.

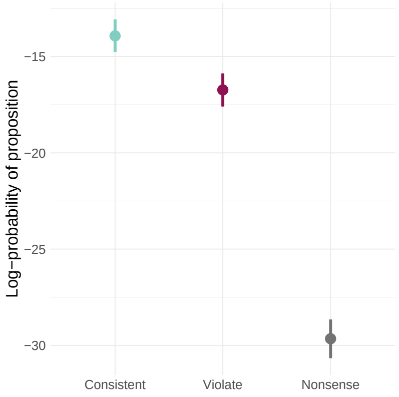

(a) NLI

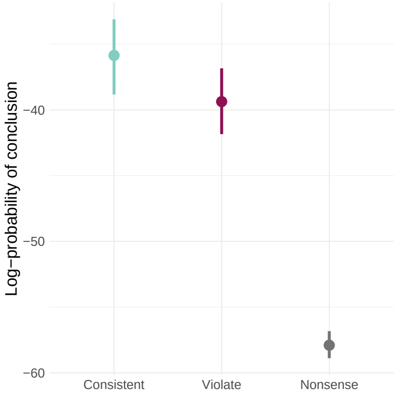

(b) Syllogisms

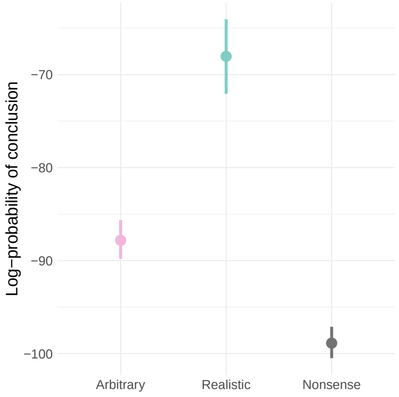

(c) Wason

Figure S7: Our datasets align with Chinchilla's expectations over text. When the likelihood of propositions or rules from each of the three tasks were evaluated, the model assigned higher likelihood to Consistent or Realistic rules than the other conditions (a-c).

B.2 Robustnesss of the main language model results to raw-likelihood scoring and few-shot prompting

In this section, we show that the content effects we observe are robust to various manipulations of the evaluation context.

B.2.1 Removing instruction prompts

In the main text experiments, we provided models with an instruction prompt that roughly matched the human instructions (cf. 73). However, it is unclear how substantial a role this prompt played in performance, and human-likeness of the content effects. In Fig. S8 we show performance of a subset of the models when removing this instruction prompt; in most cases, results are similar, with a few notable exceptions. In particular Chinchilla shows much stronger content effects on the NLI tasks without instructions.

B.2.2 Using raw likelihoods rather than Domain-Conditional PMI on the Syllogisms and Wason tasks

In the main results for the Syllogisms and Wason tasks, we scored the model using the Domain-Conditional PMI (120). However, it is also common to score language models using raw likelihood comparisons. Would we observe the same content effects in that case?

In Fig. S9 we show the results of raw-likelihood scoring. On the syllogisms tasks, this scoring method results in substantially more answer bias — several of the models say valid in response to every problem, regardless of the content or logical structure. Thus, performance is much worse overall. However, for the models that do show any variability with content, the content effects are broadly similar to those observed in the main text: the models are more likely to say an argument is valid if the conclusion is belief-consistent than if the conclusion violates beliefs. Furthermore, if the instruction prompt is removed, the bias is substantially reduced, and stronger content effects are revealed.

In the Wason tasks, the effects on accuracy are more complex. While some models perform worse without the prior correction (e.g. Chinchilla), others perform much better. In particular, PaLM-2 L achieves over 75% performance in every condition (including Arbitrary and Nonsense). However, all models that perform above chance show the same content effects observed

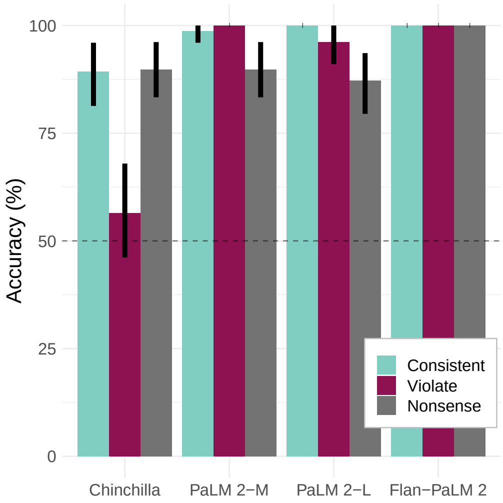

(a) NLI.

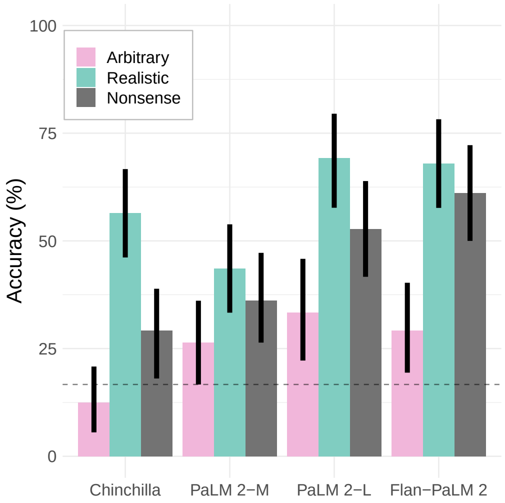

(b) Wason.

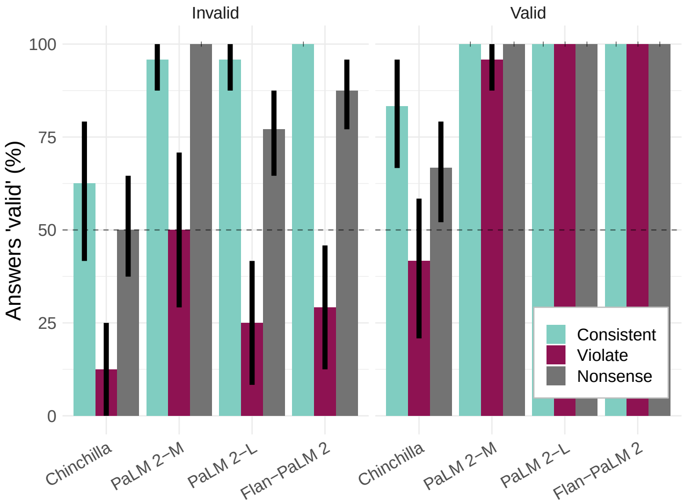

(c) Syllogisms.

Figure S8: Performance of a subset of the models when evaluated without an instruction prompt. Overall results and content effects are similar; however, in a few cases performance is noticeably impaired, particularly for Chinchilla on the Violate condition of NLI.

in the main text: better performance on Realistic than Arbitrary rules. (In SI B.2.3 we also explore the effect of scoring with raw likelihoods on the individual card choices on the Wason task.)

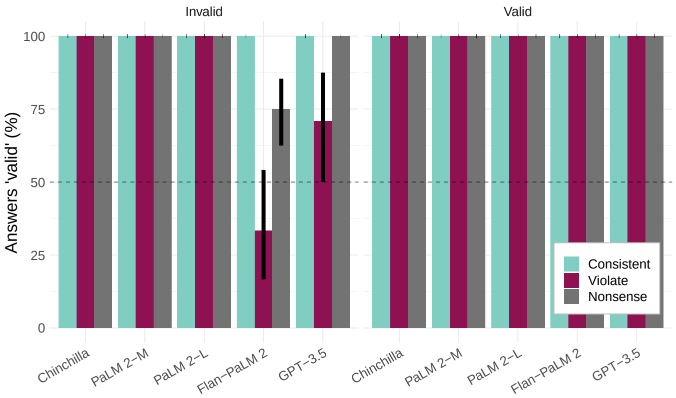

(a) Syllogisms

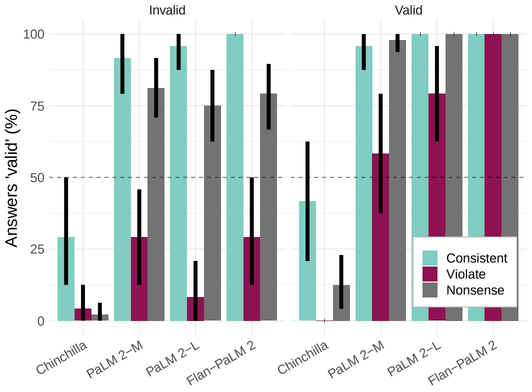

(b) Syllogisms, no instruction prompt

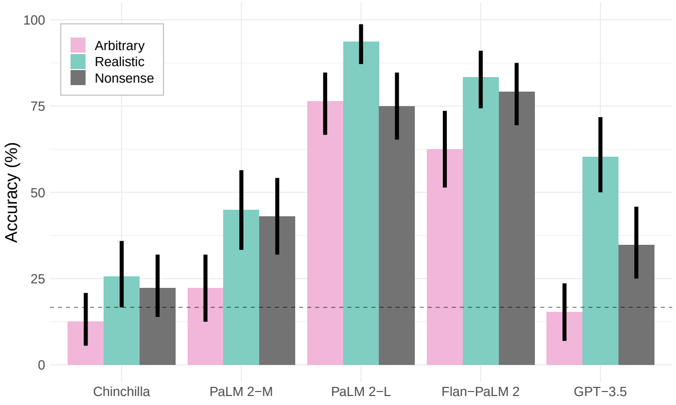

(c) Wason

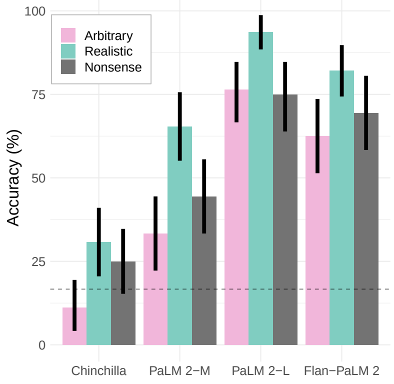

(d) Wason, no instruction prompt

Figure S9: Scoring using the raw answer likelihoods — rather than the Domain-Conditional PMI prior correction — for the Syllogisms and Wason tasks. (a) On the syllogisms tasks, removing the prior correction results in substantial answer biases for many models: much greater likelihood to say "valid" than "invalid." Overall performance is much worse due to this bias; indeed, several models answer "valid" for every argument in every condition. However, for those that do not — Flan-PaLM 2 and GPT-3.5 — the direction of the content effects is as in the main text: the models are more likely to answer "valid" if the conclusion is belief-consistent. Note that the left and right sub-panels are invalid and valid arguments, respectively. (b) However, the answer bias on the syllogisms with raw-likelihood scoring seems to be strongly driven by the instruction prompt; without the prompt, the raw likelihoods yield less biased responses, and strong overall content effects. (c-d) On the Wason tasks, with or without the instruction prompt, removing the prior correction improves performance from some models, but hurts performance from others. Regardless, all models show the same pattern of content effects: facilitation in the Realistic rules compared to Arbitrary. (Compare to Figs. 4 and 5, respectively, which use DC-PMI scoring.))

B.2.3 Effects of scoring method and answer order on the Wason answer choices

In Fig. S10 we show the effect of scoring method (DC-PMI vs. raw likelihoods) and the order in which the cards were presented (antecedent cards first or consequent cards first) on the models' answer choices on the Wason task. Scoring method does affect the error distribution fairly substantially, even where accuracy is similar; answer order has smaller effects.

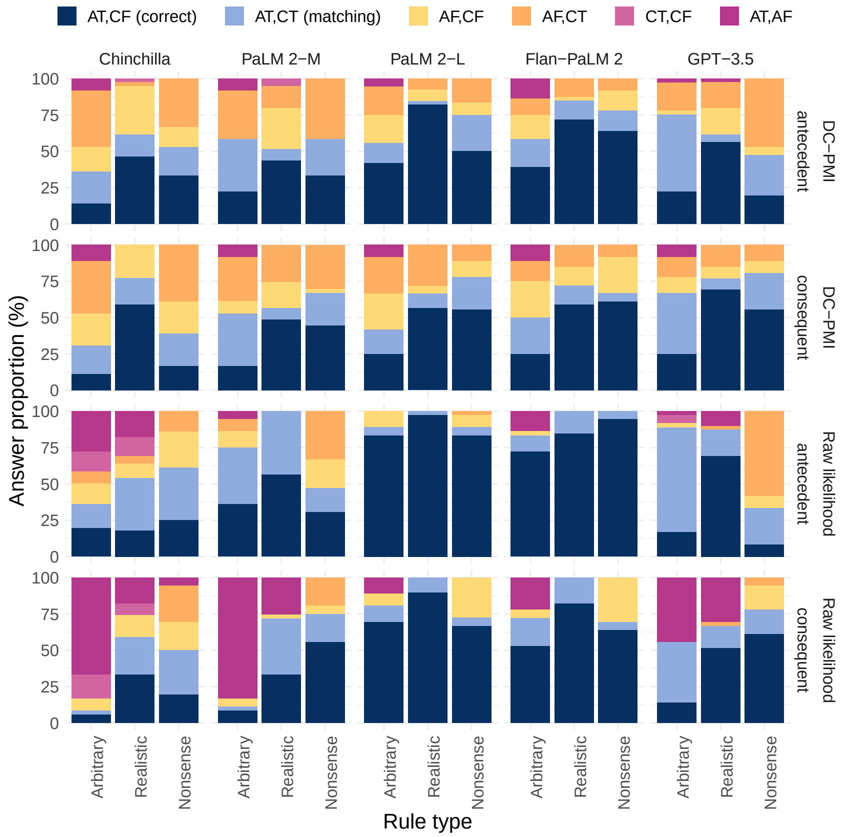

Figure S10: Effect of scoring method (DC-PMI in the top two rows vs. raw likelihoods in the bottom two) and ordering of the cards (antecedent cards first or consequent cards first; respectively in rows 1 and 3, and 2 and 4) on model choices. The DC-PMI prior correction does shift error patterns somewhat, and the models commit relatively more of the AT,AF answers with raw likelihood scoring, while with the DC-PMI scoring, the humans commit more of these errors than the models. The ordering of the cards does not have too substantial an effect, particularly with DC-PMI scoring. Generally, content effects — that is, the advantage of the Realistic rules over arbitrary ones — persists regardless of scoring method or order.

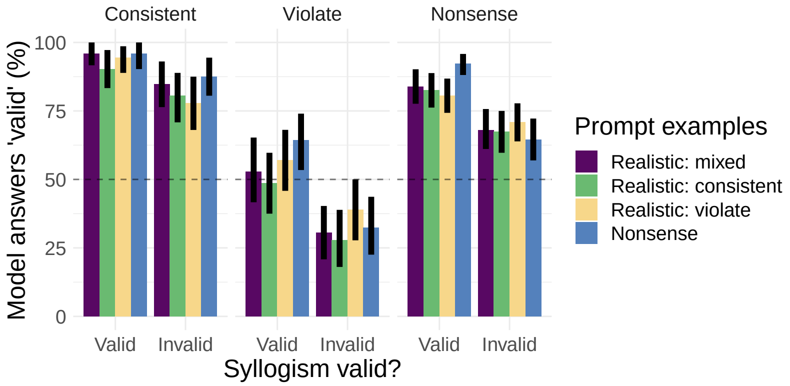

Figure S11: Chinchilla evaluated 5-shot on the syllogisms task, with different types of prompt examples. Content effects are very slightly reduced relative to the original experiments, but remain robust. The particular type of problems used in the prompt examples do not strongly affect performance. (The "Realistic: mixed" condition includes realistic examples from both the consistent and violate subsets.)

B.2.4 Few-shot prompting of Chinchilla

In all the main text experiments, we evaluated the models zero-shot, with only instructions. However, language model performance is generally improved by few-shot prompting (e.g. 7). We therefore evaluated whether few shot prompting with different kinds of prompt examples would alter the content effects we observed. (Note that, for computational reasons, we restrict these analyses to the Chinchilla model.) When we present a few-shot prompt of examples of the task to the model, the examples are presented with correct answers, and each example (as well as the final probe) is separated from the previous example by a single blank line.

In Fig. S11 we show 5-shot prompting results for Chinchilla on the Syllogisms tasks. Content effects are slightly weaker than without the examples, but remain robust.

In Fig. S12 we show 5-shot prompting results for Chinchilla on the Wason selection tasks. Content effects are exaggerated with the 5-shot prompts, because the model improves noticeably at Realistic rules, but improves less (if at all) on Arbitrary ones. We also see a noticeable effect of the type of examples used in the prompt, with Realistic examples offering optimal benefits.

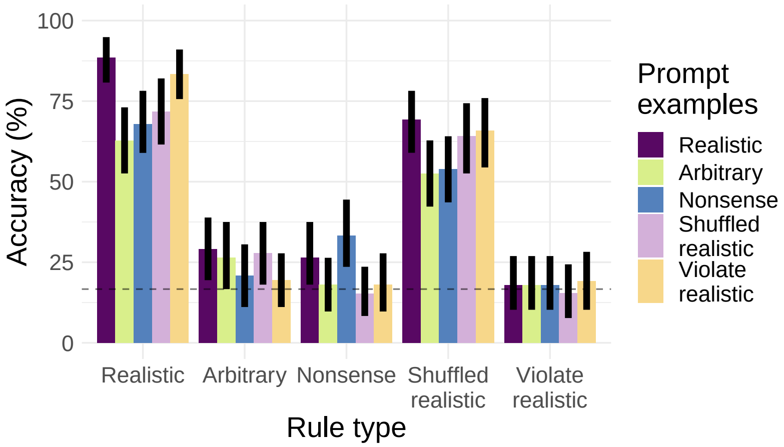

Figure S12: Chinchilla evaluated 5-shot on the Wason task, with different types of prompt examples. Again, content effects remain strong — or are even amplified — with few-shot prompts. Realistic prompt examples appear to be most beneficial overall, but especially for realistic and shuffled realistic probes, thus they actually enhance content effects. Other types of prompts are generally helpful in a more limited set of conditions; there may be an overall benefit to prompts matching probes.

B.3 Gemini & Gemma models and Chain-of-Thought prompting benefits

In this section, we evaluate the Gemini Pro & Ultra models (66), along with their smaller open-source counterpart Gemma 7B (67) on the Syllogisms and Wason tasks. This serves to validate that our results hold with these newer models, and whether the smaller open-sourced Gemma 7B model shows similar patterns. These models are also respectively accessible through public APIs, and open-source. Moreover, we explore several chain-of-thought (65) prompting styles, to see whether the models can use logic more reliably if they generate intermediate thinking steps before the answer.

We first evaluated the models with No CoT, as in all other experiments in this papaer. Both Gemini models show significant content effects on the Syllogisms and Wason tasks. Gemma 7B shows significant content effects on the Syllogisms task, but not on the Wason task, perhaps because it shows relatively little ability to solve more complex reasoning tasks overall—larger models may be necessary.

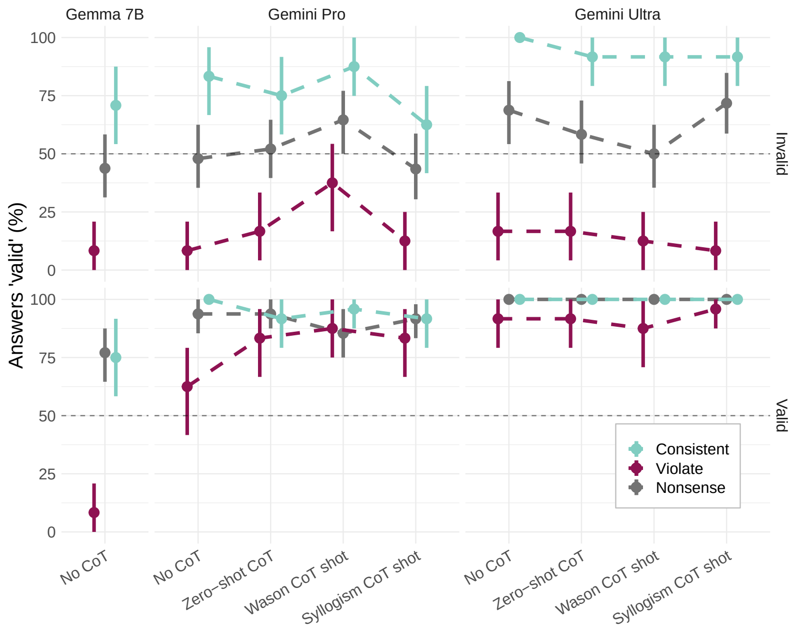

Figure S13: Detailed results of evaluating Gemini & Gemma models on the syllogism tasks, with chain-of-thought prompts for the larger models. Gemma 7B shows strong content biases, but little effect of logic. The effects of chain-of-thought prompting are relatively small.

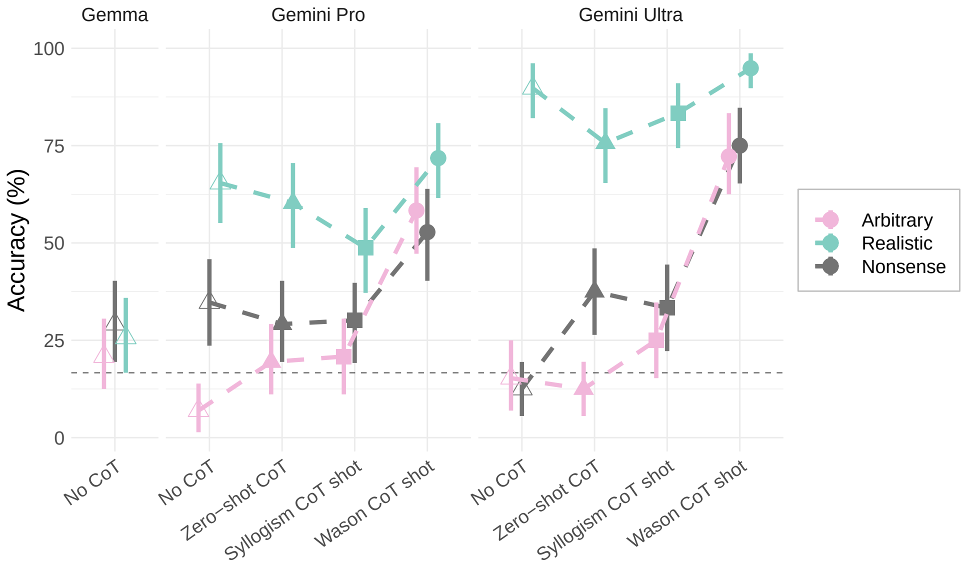

Figure S14: Detailed results of evaluating Gemini & Gemma models on the Wason tasks, with chain-of-thought prompts for the larger models. Gemma shows few content effects, and low performance overall. There are, however, noticeable effects of chain-of-thought prompting, particularly with examples and for the larger Gemini Ultra model.

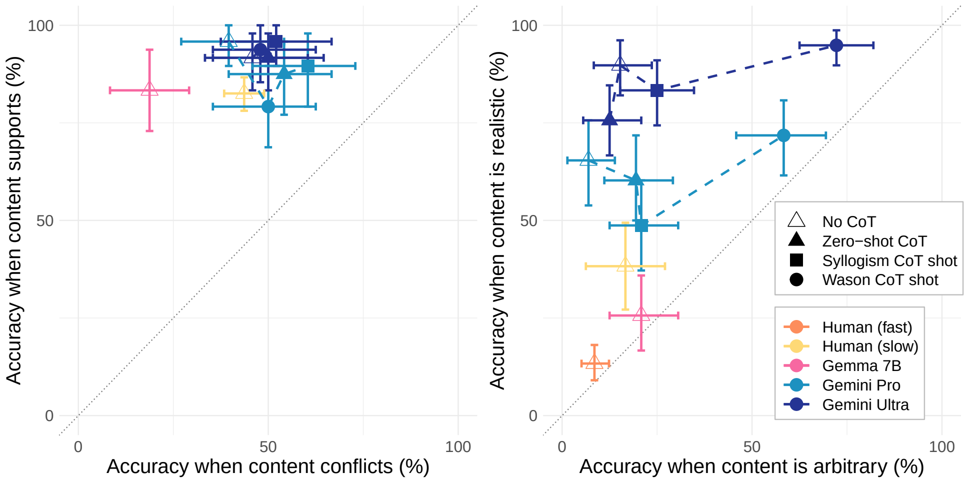

(a) Syllogisms

(b) Wason

Figure S15: Evaluating Gemini & Gemma models on the Wasons and syllogisms tasks, including chain-of-thought prompts for the larger models. The larger models show strong content effects, and perform comparably or better than humans. Gemma 7B shows strong content effects on the syllogism task, but performs fairly poorly overall. Chain-of-thought prompting can in some cases push the models to use logic more and content less, yielding higher accuracy on conflict/arbitrary trials, but sometimes slightly reducing accuracy on realistic tasks (i.e., shifting performance rightward and slightly downward on these plots). The clearest demonstration of this is on the Wason tasks, where models given an example chain-of-thought demonstrating correct reasoning on an arbitrary Wason task shows greatly improved performance on Arbitrary tasks, along with moderate improvements on the Realistic tasks. Gemini Ultra shows little effect of prompting on the syllogisms tasks, but Gemini Pro does show a more modest shift rightward and down.

We then explored chain of thought prompting. In these experiments, we give the human-matched instructions, then a chain-of-thought prompt, then present the problem, then allow the model to freely generate text until an end of response token. We append these generations to the prompt and question, and then prompt with "Answer:" and score the answers as in other experiments.

We explored several types of chain-of-thought prompts. The simplest is a zero-shot chain-of-thought prompt (cf. 10), where we simply add "explain your reasoning process before giving your answer" between the instructions and the question. We also consider two one-shot chain-of-thought prompts, either giving an example of the same task (with a handwritten chain of thought detailed below), or an example of the *other* task — i.e., prompting with an example of the Wason task for syllogisms, and vice versa. The idea with the latter is that it may help to just demonstrate correct logical reasoning, without needing to give away too much about the task-specific strategies.

We show the results of the chain-of-thought experiments in Figs. S13 and S14, along with a summary in Fig. S15. On the Wason selection task, chain-of-thought prompting with an example—and especially a Wason task examples—shifts the models noticeably towards more logic-driven responses, with correspondingly boosted performance on the Arbitrary and Nonsense conditions. We note that this is in contrast to some of the original human experiments of Wason (1) in which he found that teaching people how to reason about the task-specific strategy of falsification before giving them the Wason task did not substantially boost performance. On the syllogisms tasks, the effect of chain-of-thought is relatively small, though some of the prompts do have significant benefits on performance, particularly for the smaller Gemini Pro model. We quantify these results below using mixed-effects logistic regressions (Tables S3 & S4), and show some examples of generated reasoning traces in Table S2.

In summary, at least in some cases chain-of-thought prompting can push models to rely on

logic more, and increase accuracy in difficult conditions.

The exact chain-of-thought prompts we used are included below for reference.

```
cot_prompts = {
'Zero-shot-explain-reasoning': (
'In this task, you will have to answer a series of questions. You will have
    ↪  to choose the best answer to complete a sentence, paragraph, or
    ↪ question. Please answer them to the best of your ability, and explain
    ↪  the reasoning process before giving your answer.\n\nPlease answer
    ↪ the following question carefully:\n'),
'Syllogism-shot-explain-reasoning': (
'In this task, you will have to answer a series of questions. You will have
    ↪  to choose the best answer to complete a sentence, paragraph, or
    ↪ question. Please answer them to the best of your ability, and explain
    ↪  the reasoning process before giving your answer.\n\nQuestion #1:\
    ↪ nPlease assume that the first two sentences in the argument are true.
    ↪  Determine whether the argument is valid, that is, whether the
    ↪ conclusion follows from the first two sentences:\nArgument:\nAll
    ↪ grosp are crand.\nNo threck are grosp.\nConclusion: No threck are
    ↪ crand.\nReasoning: The fact that all grosp are crand does not
    ↪ necessarily imply that all crand are grosp.Thus, there could be some
    ↪ threck that are crand but not grosp. So the conclusion does not
    ↪ follow logically from the argument.\nAnswer: The argument is invalid
    ↪ .\n\nQuestion #2:\n'
),
'Wason-shot-explain-reasoning': (
'In this task, you will have to answer a series of questions. You will have
    ↪  to choose the best answer to complete a sentence, paragraph, or
    ↪ question. Please answer them to the best of your ability, and explain
    ↪  the reasoning process before giving your answer.\n\nQuestion #1:\
    ↪ nThe following cards each have a word on one side and a number on
    ↪ their other side. The rule is that if the cards have a French word
    ↪ then they must have a positive number. Which of the cards would you
    ↪ need to flip over to ensure that the rule is being followed?\ncard:
    ↪ -1\ncard: 4\ncard: sombrero\ncard: chapeau\nReasoning: This is an if-
    ↪ then rule, with two cards showing values relating to the antecedent (
    ↪ words in French or Spanish), and two cards relating to the consequent
    ↪  (a positive and a negative number). Each set has one card that
    ↪ matches the corresponding proposition, and one that does not. First,
    ↪ we need to check the card that matches the antecedent ("chapeau"),
    ↪ because if it has a negative number on the back it would violate the
    ↪ rule. We also need to check the card that does not match the
    ↪ consequent ("-1"), because if it had a french word onthe other side
    ↪ it would violate the rule. We do not need to flip over the other two
    ↪ cards, because regardless of what is on the other side, they will be
    ↪ compatible with the rule.\nAnswer: You need to flip over the ones
    ↪ showing ""chapeau"" and ""-1"" to ensure that the rule is being
    ↪ followed.\n\nQuestion #2:\n'),
}
```

```
Reasoning: To check if the rule is being followed, we need to check if
↪  passengers traveling outside the US have passports. Therefore, we need
↪  to flip over the cards with destinations outside the US and those with
↪  driver's licenses (which are not passports).

Answer: The cards with ""Buenos Aires"" and ""driver's license""
```

(a) Realistic, correct

```
Reasoning: To ensure the rule is being followed, we need to check if
↪  passengers traveling outside the US have a passport. So, we need to
↪  flip over the cards with destinations outside the US and check if they
↪  have a passport on the other side.
Answer: The cards with San Francisco and Buenos Aires destinations should
↪  be flipped over
```

(b) Realistic, incorrect

```
Reasoning: To ensure the rule is followed, we need to check if any cards
↪  with a beverage containing caffeine do not have a material that
↪  conducts electricity on the other side. Therefore, we need to flip over
↪  the ""coffee"" card (which contains caffeine) and the ""wood"" card
↪  (which does not conduct electricity).
Answer: The ""coffee"" and ""wood"" cards.
```

(c) Arbitrary, correct

```
Reasoning: The rule states that cards with plural words must have a
↪  positive emotion. Therefore, we need to flip over the card with the
↪  plural word ""crises"" to check if it has a positive emotion on the
↪  other side.
Answer: The card with ""crises"".
```

(d) Arbitrary, incorrect

```
Reasoning: Similar to the previous question, we need to check the card that
↪  matches the antecedent (""more pron"") and the card that does not match
↪  the consequent (""less stope"").
Answer: You need to flip over the ones showing ""more pron"" and ""less
↪  stope"" to ensure that the rule is being followed.
```

(e) Nonsense, correct

```
Reasoning: Similar to the previous question, we need to check the sheet
↪  that matches the antecedent (""less gluff""), because if it has less
↪  caft on the back it would violate the rule. We also need to check the
↪  sheet that does not match the consequent (""more caft""), because if it
↪  had more gluff on the other side it would violate the rule.
Answer: You need to flip over the ones showing ""less gluff"" and ""more
↪  caft"" to ensure that the rule is being followed.
```

(f) Nonsense, incorrect

Table S2: Example correct and incorrect/incomplete reasoning traces generated by Gemini Ultra on the Wason tasks. Note that the model almost always generates an answer in the reasoning trace, before being scored for afinal answer. Common errors include only considering the forward direction, or stating the principles correctly but getting the propositions wrong (f).

```
Generalized linear mixed model fit by maximum likelihood (Laplace
  Approximation) [glmerMod]
 Family: binomial  ( logit )
Formula: response_correct ~ logic_belief_consistent * (valid_readable_f +
    chain_of_thought_condition) + (1 | syllogism_name)
   Data: gemini_syllogism_model_df %>% filter(subject == "Gemini Pro")
Control: glmerControl(optimizer = "bobyqa", optCtrl = list(maxfun = 50000))

     AIC      BIC   logLik deviance df.resid
   672.4    723.4   -325.2    650.4      755

Scaled residuals:
    Min      1Q  Median      3Q     Max
-9.6520 -0.3947  0.2053  0.4234  3.4274

Random effects:
 Groups         Name        Variance Std.Dev.
 syllogism_name (Intercept) 1.338    1.157
Number of obs: 766, groups:  syllogism_name, 60

Fixed effects:
                                                                     Estimate  Std. Error  z value  Pr(>|z|)
(Intercept)                                                           1.56608     0.29849    5.247  1.55e-07 ***
logic_belief_consistent                                               2.32628     0.39829    5.841  5.20e-09 ***
valid_readable_f1                                                     2.57791     0.29158    8.841   < 2e-16 ***
chain_of_thought_conditionZero-shot CoT                              -0.17035     0.32000   -0.532   0.59449
chain_of_thought_conditionWason CoT shot                             -0.81261     0.31247   -2.601   0.00931 **
chain_of_thought_conditionSyllogism CoT shot                          0.09995     0.32589    0.307   0.75907
logic_belief_consistent:valid_readable_f1                            -0.65688     0.34896   -1.882   0.05978 .
logic_belief_consistent:chain_of_thought_conditionZero-shot CoT      -1.20966     0.49096   -2.464   0.01375 *
logic_belief_consistent:chain_of_thought_conditionWason CoT shot     -1.44160     0.47467   -3.037   0.00239 **
logic_belief_consistent:chain_of_thought_conditionSyllogism CoT shot -1.33061     0.49845   -2.669   0.00760 **
---
Signif. codes:  0 ‘***’ 0.001 ‘**’ 0.01 ‘*’ 0.05 ‘.’ 0.1 ‘ ’ 1
```

(a) Gemini Pro

```
Generalized linear mixed model fit by maximum likelihood (Laplace
  Approximation) [glmerMod]
 Family: binomial  ( logit )
Formula: response_correct ~ logic_belief_consistent * (valid_readable_f +
    chain_of_thought_condition) + (1 | syllogism_name)
   Data: gemini_syllogism_model_df %>% filter(subject == "Gemini Ultra")
Control: glmerControl(optimizer = "bobyqa", optCtrl = list(maxfun = 50000))

     AIC      BIC   logLik deviance df.resid
   314.5    365.5   -146.2    292.5      755

Scaled residuals:
    Min      1Q  Median      3Q     Max
-6.5914 -0.0892  0.0000  0.1057  7.8037

Random effects:
 Groups         Name        Variance Std.Dev.
 syllogism_name (Intercept) 10.04    3.169
Number of obs: 766, groups:  syllogism_name, 60

Fixed effects:
                                                                    Estimate  Std. Error  z value  Pr(>|z|)
(Intercept)                                                          10.4296     68.2699    0.153   0.87858
logic_belief_consistent                                              11.7653     68.2690    0.172   0.86317
valid_readable_f1                                                    24.0916    136.5340    0.176   0.85994
chain_of_thought_conditionZero-shot CoT                               0.8052      0.4904    1.642   0.10062
chain_of_thought_conditionWason CoT shot                              1.3307      0.5129    2.594   0.00948 **
chain_of_thought_conditionSyllogism CoT shot                          0.3528      0.4849    0.728   0.46686
logic_belief_consistent:valid_readable_f1                            15.4874    136.5409    0.113   0.90969
logic_belief_consistent:chain_of_thought_conditionZero-shot CoT      -0.4130      0.6780   -0.609   0.54246
logic_belief_consistent:chain_of_thought_conditionWason CoT shot      0.1981      0.7266    0.273   0.78510
logic_belief_consistent:chain_of_thought_conditionSyllogism CoT shot -0.2020      0.6599   -0.306   0.75953
---
Signif. codes:  0 ‘***’ 0.001 ‘**’ 0.01 ‘*’ 0.05 ‘.’ 0.1 ‘ ’ 1
```

(b) Gemini Ultra

Table S3: Chain-of-thought prompting effects on the Syllogism tasks.

```
Generalized linear mixed model fit by maximum likelihood (Laplace
  Approximation) [glmerMod]
 Family: binomial  ( logit )
Formula: response_correct ~ wason_condition * chain_of_thought_condition +
    (1 | wason_name)
   Data: gemini_cot_regression_df %>% filter(subject == "Gemini Pro")
 Offset: offset_wason_prior_log_odds
Control: glmerControl(optimizer = "bobyqa", optCtrl = list(maxfun = 5000))

     AIC      BIC   logLik deviance df.resid
   891.2    953.5   -432.6    865.2      876

Scaled residuals:
    Min      1Q  Median      3Q     Max
-3.0114 -0.4882 -0.1471  0.5387  5.7853

Random effects:
 Groups     Name        Variance Std.Dev.
 wason_name (Intercept) 2.63     1.622
Number of obs: 889, groups:  wason_name, 37

Fixed effects:
                                                                                          Estimate Std. Error  z value  Pr(>|z|)
(Intercept)                                                                                0.38115    0.35046    1.088   0.27679
wason_conditionrealistic_vs_arbitrary_nonsense                                             4.43213    1.06723    4.153  3.28e-05 ***
wason_conditionarbitrary_vs_nonsense                                                      -1.87560    0.77993   -2.405   0.01618 *
chain_of_thought_conditionZero-shot CoT                                                    0.22848    0.27740    0.824   0.41013
chain_of_thought_conditionSyllogism CoT shot                                               0.08235    0.27556    0.299   0.76507
chain_of_thought_conditionWason CoT shot                                                   1.81717    0.28392    6.400  1.55e-10 ***
wason_conditionrealistic_vs_arbitrary_nonsense:chain_of_thought_conditionZero-shot CoT    -2.31584    0.87977   -2.632   0.00848 **
wason_conditionarbitrary_vs_nonsense:chain_of_thought_conditionZero-shot CoT              -0.08993    0.58581   -0.154   0.87800
wason_conditionrealistic_vs_arbitrary_nonsense:chain_of_thought_conditionSyllogism CoT shot -2.82748  0.87494   -3.232   0.00123 **
wason_conditionarbitrary_vs_nonsense:chain_of_thought_conditionSyllogism CoT shot          0.63881    0.58097    1.100   0.27153
wason_conditionrealistic_vs_arbitrary_nonsense:chain_of_thought_conditionWason CoT shot   -4.06606    0.89740   -4.531  5.87e-06 ***
wason_conditionarbitrary_vs_nonsense:chain_of_thought_conditionWason CoT shot              0.76847    0.59222    1.298   0.19442
---
Signif. codes:  0 '***' 0.001 '**' 0.01 '*' 0.05 '.' 0.1 ' ' 1
```

(a) Gemini Pro

```
Generalized linear mixed model fit by maximum likelihood (Laplace
  Approximation) [glmerMod]
 Family: binomial  ( logit )
Formula: response_correct ~ wason_condition * chain_of_thought_condition +
    (1 | wason_name)
   Data: gemini_cot_regression_df %>% filter(subject == "Gemini Ultra")
 Offset: offset_wason_prior_log_odds
Control: glmerControl(optimizer = "bobyqa", optCtrl = list(maxfun = 5000))

     AIC      BIC   logLik deviance df.resid
   795.6    857.8   -384.8    769.6      875

Scaled residuals:
    Min      1Q  Median      3Q     Max
-6.3063 -0.4423  0.1485  0.4384  4.5047

Random effects:
 Groups     Name        Variance Std.Dev.
 wason_name (Intercept) 1.279    1.131
Number of obs: 888, groups:  wason_name, 37

Fixed effects:
                                                                                          Estimate Std. Error  z value  Pr(>|z|)
(Intercept)                                                                                1.03813    0.29194    3.556  0.000377 ***
wason_conditionrealistic_vs_arbitrary_nonsense                                             3.18763    0.82559    3.861  0.000113 ***
wason_conditionarbitrary_vs_nonsense                                                      -4.97592    0.73262   -6.792  1.11e-11 ***
chain_of_thought_conditionZero-shot CoT                                                    0.04652    0.28492    0.163  0.870315
chain_of_thought_conditionSyllogism CoT shot                                               0.51338    0.27927    1.838  0.066013 .
chain_of_thought_conditionWason CoT shot                                                   2.60611    0.32480    8.024  1.02e-15 ***
wason_conditionrealistic_vs_arbitrary_nonsense:chain_of_thought_conditionZero-shot CoT     0.64221    0.82950    0.774  0.438804
wason_conditionarbitrary_vs_nonsense:chain_of_thought_conditionZero-shot CoT               2.91659    0.67872    4.297  1.73e-05 ***
wason_conditionrealistic_vs_arbitrary_nonsense:chain_of_thought_conditionSyllogism CoT shot -0.48386  0.77861   -0.621  0.534315
wason_conditionarbitrary_vs_nonsense:chain_of_thought_conditionSyllogism CoT shot          2.11020    0.69390    3.041  0.002357 **
wason_conditionrealistic_vs_arbitrary_nonsense:chain_of_thought_conditionWason CoT shot   -1.60966    0.86292   -1.865  0.062130 .
wason_conditionarbitrary_vs_nonsense:chain_of_thought_conditionWason CoT shot              2.72734    0.82288    3.314  0.000918 ***
---
Signif. codes:  0 '***' 0.001 '**' 0.01 '*' 0.05 '.' 0.1 ' ' 1
```

(b) Gemini Ultra

Table S4: Chain-of-thought prompting effects on the Wason tasks.

B.4 The Wason rule propositions have similar difficulty across conditions

One possible confounding explanation for our Wason results would be that the base propositions that form the antecedents and consequents of the rules have different difficulty across conditions—this could potentially explain why the realistic rules and shuffled realistic rules are both easier than abstract or nonsesnse ones. To investigate this possibility, we tested the difficulty of identifying which of the options on the cards matched the corresponding proposition. Specifically, for the antecedent of the rule "if the workers work as a doctor then they must have received an MD" we prompted Chinchilla with a question like:

```
Which choice better matches "work as a doctor"?
choice: surgeon
choice: janitor
Answer:
```

And then gave a two-alternative forced choice between 'surgeon' and 'janitor'. To avoid order biases, we repeated this process for both possible answer choice orderings in the prompt, and then aggregated likelihoods across these and chose the highest-likelihood answer.

By this metric, we find that there are no substantial differences in difficulty across the rule types (Fig. S16)—in fact, arbitrary rule premises are numerically slightly easier, though the differences are not significant. Thus, the effects we observed are not likely to be explained by the base difficulty of verifying the component propositions.

B.5 Additional recombined realistic conditions for the Wason tasks

The Wason task rules can be realistic or unrealistic in multiple ways. For example, the component propositions can be realistic even if the relationship between them is not. We therefore generate two variations on realistic rules:

Shuffled realistic rules, which combine realistic components in nonsensical ways (e.g. "if the

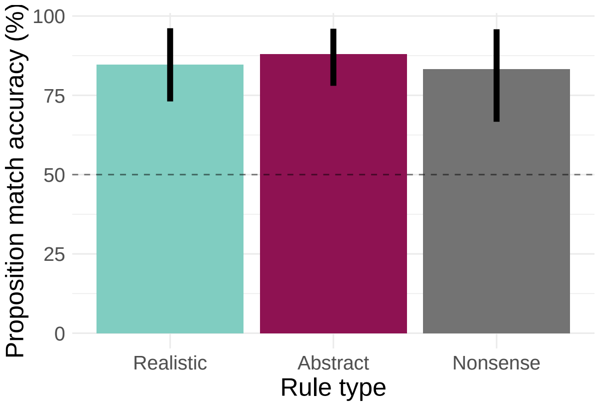

Figure S16: The component propositions (antecedents and consequents) of the Wason rules have similar difficulty across conditions. This plot shows Chinchilla's accuracy on forced choices of which instance matches a proposition, across conditions. (Note that the shuffled realistic rules use the same component propositions as the realistic rules.)

passengers are traveling outside the US, then they must have received an MD").

Violate realistic rules, which directly violate the expected relationship (e.g. "if the passengers are flying outside the US, then they must have shown a drivers license [not a passport]").

We also evaluated models and humans on these rules. For shuffled rules, results are well above chance. Surprisingly, one family of models (PaLM 2) even perform better at shuffled realistic than realistic rules. For violate rules, by contrast, performance is generally close to chance. It appears that the model reasons more accurately about rules formed from realistic propositions, particularly if the relationships between propositions in the rule are also realistic, but even to some degree if they are shuffled in nonsensical ways that do not directly violate expectations. However, if the rules strongly violate beliefs, performance is low. Humans generally perform poorly on either rule variant.

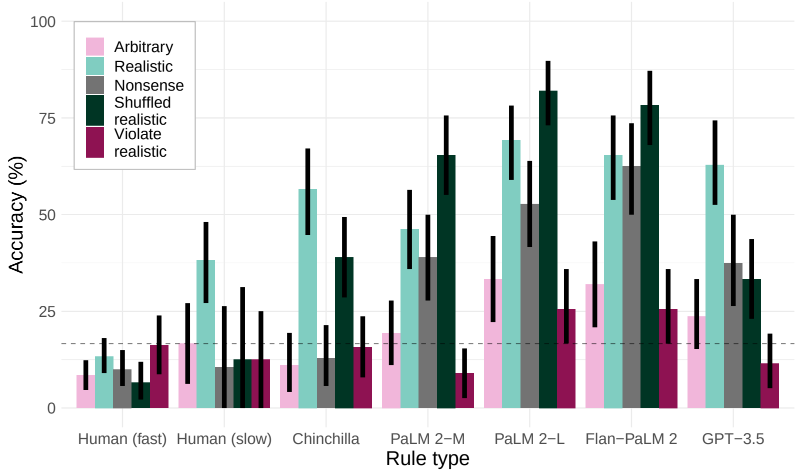

Figure S17: Evaluating models and humans on shuffled realistic and violate versions of the Wason rules. Humans

B.6 Human performance on the Wason tasks, in our original sample & replication

As mentioned in the main text, after collecting our original sample on the Wason task, we recruited an additional set of participants to whom we offered a performance bonus on this task, in an attempt to increase performance. We present the results broken down by sample in Fig. S18. We performed mixed-effects logistic regressions (Table S5) to test for an improvement in performance in the sample with a performance bonus; this effect was marginally significant. However, performance remains low overall, and we do not observe a significant difference in the content effect.

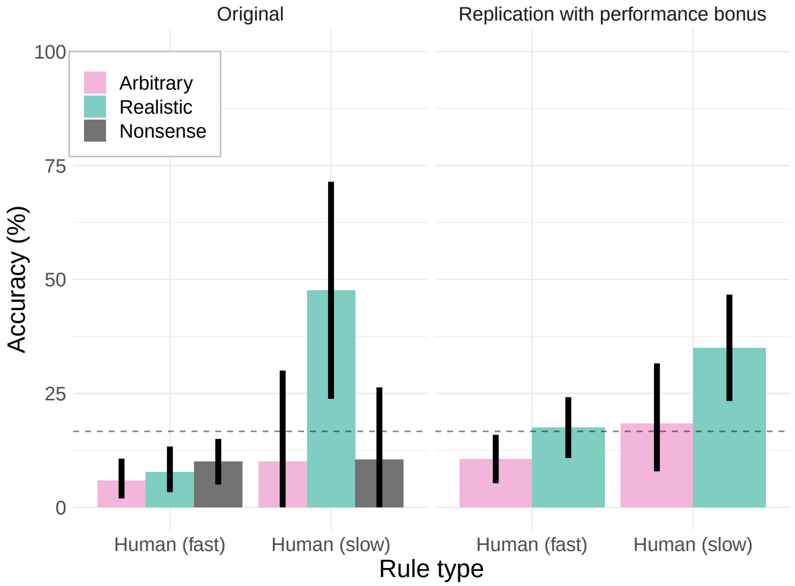

Figure S18: Breakdown of human results in our original experiment, and our replication (where we also added a performance bonus of 0.5 GPB for the Wason question). We observe a significant advantage for the slower humans in the Realistic condition in each case. The performance bonus does not seem to clearly improve performance.

```
Generalized linear mixed model fit by maximum likelihood (Laplace
  Approximation) [glmerMod]
 Family: binomial  ( logit )
Formula: response_correct ~ wason_condition + replication_experiment +
    (1 | wason_name)
   Data: wason_human_correct_df

     AIC      BIC   logLik deviance df.resid
   476.1    493.5   -234.0    468.1      570

Scaled residuals:
    Min      1Q  Median      3Q     Max
-0.7247 -0.4879 -0.3449 -0.2718  3.9974

Random effects:
 Groups     Name        Variance Std.Dev.
 wason_name (Intercept) 0.1704   0.4127
Number of obs: 574, groups:  wason_name, 25

Fixed effects:
                           Estimate Std. Error z value Pr(>|z|)
(Intercept)                 -2.6190     0.3029  -8.646  < 2e-16 ***
wason_conditionRealistic     0.8261     0.3066   2.694  0.00706 **
replication_experimentTRUE   0.5274     0.2695   1.957  0.05034 .
---
Signif. codes:  0 '***' 0.001 '**' 0.01 '*' 0.05 '.' 0.1 ' ' 1
```

(a) Additive model.

```
Generalized linear mixed model fit by maximum likelihood (Laplace
  Approximation) [glmerMod]
 Family: binomial  ( logit )
Formula: response_correct ~ wason_condition * replication_experiment +
    (1 | wason_name)
   Data: wason_human_correct_df

     AIC      BIC   logLik deviance df.resid
   477.7    499.5   -233.9    467.7      569

Scaled residuals:
    Min      1Q  Median      3Q     Max
-0.7198 -0.4788 -0.3544 -0.2523  4.3184

Random effects:
 Groups     Name        Variance Std.Dev.
 wason_name (Intercept) 0.1762   0.4197
Number of obs: 574, groups:  wason_name, 25

Fixed effects:
                                                    Estimate Std. Error z value
(Intercept)                                          -2.7723     0.4162  -6.662
wason_conditionRealistic                              1.0550     0.5078   2.078
replication_experimentTRUE                            0.7380     0.4606   1.602
wason_conditionRealistic:replication_experimentTRUE  -0.3262     0.5673  -0.575
                                                    Pr(>|z|)
(Intercept)                                         2.71e-11 ***
wason_conditionRealistic                              0.0378 *
replication_experimentTRUE                            0.1091
wason_conditionRealistic:replication_experimentTRUE   0.5653
---
Signif. codes:  0 '***' 0.001 '**' 0.01 '*' 0.05 '.' 0.1 ' ' 1
```

(b) Interaction model.

Table S5: Mixed-effects linear regressions for differences in human performance on the replication sample on the Wason task. We do observe a marginally-significant effect of the experiment in the additive model (top). However, we do not observe significant differences in the content effect in an interaction model (bottom).

B.7 Human response time distributions on the Wason tasks

In Fig. S19 we show the distribution of response times for humans in the Wason tasks. There is a mean difference in response times, with participants spending about 12 seconds longer on Realistic questions on average. This difference may be due to the time needed to read the extra sentences giving the realistic context, or to the participants engaging more deeply with the problems that seem more sensible. However, in SI C.3.1 we show that this difference alone does not explain the advantage of the Realistic conditions.

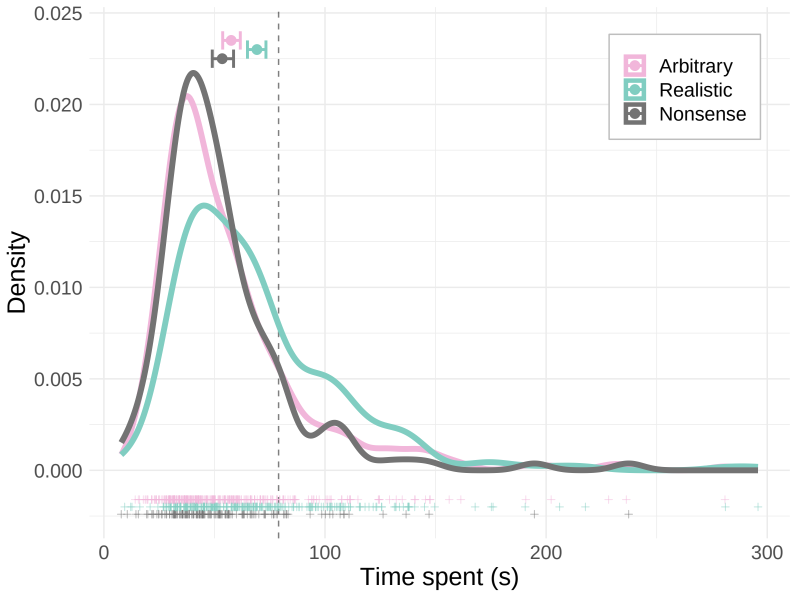

Figure S19: Human response time distributions on the Wason tasks. The Realistic condition results in significantly longer response times. The vertical dashed line indicates the cutoff for "slow" subject group; 85% of the subjects were faster than this in the original experiment.

B.7.1 Response time effects on NLI and syllogisms

Given the strong effect of response time on Wason task performance, we also analyzed the effects on the NLI and Syllogism tasks (Figs. S20 & S20; Table S6). In these tasks we do not see clear effects, though there are hints of an interesting potential interaction in the syllogisms task.

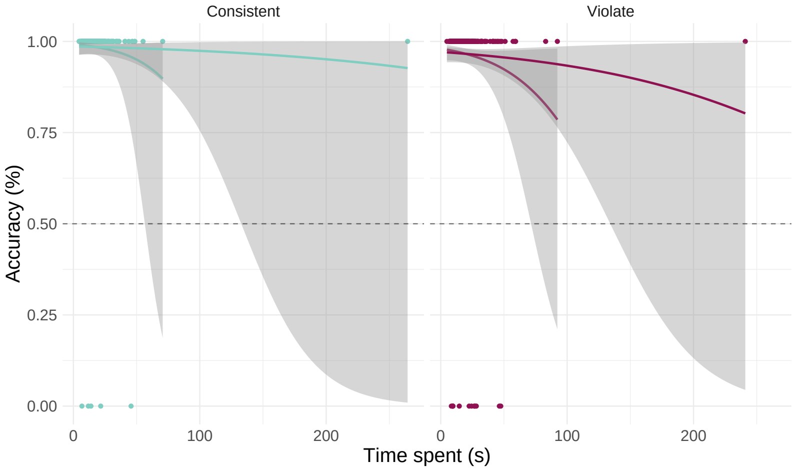

Figure S20: There is little effect of response time on accuracy in the NLI tasks.

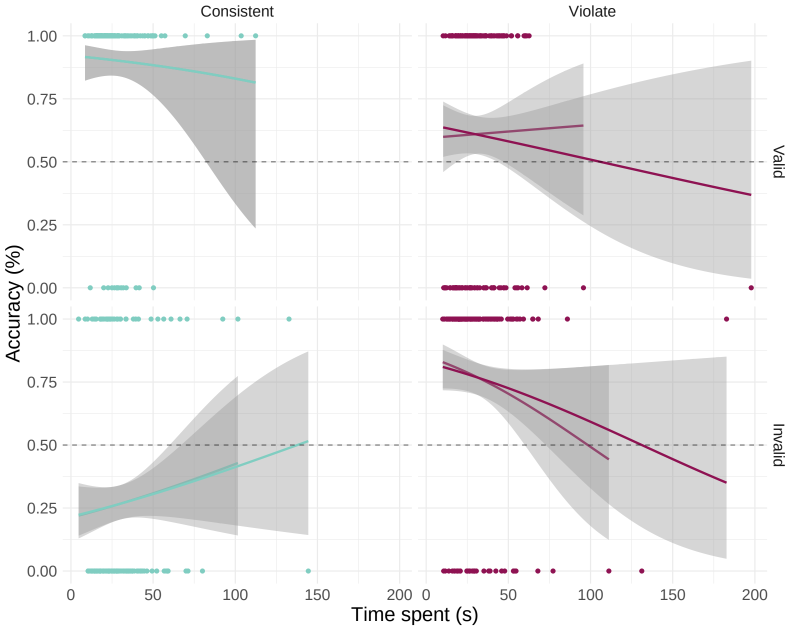

Figure S21: Effects of response time on accuracy on the syllogisms task.

B.8 Item-level effects

In this section, we perform item level analyses for each task.

```
Generalized linear mixed model fit by maximum likelihood (Laplace
  Approximation) [glmerMod]
 Family: binomial  ( logit )
Formula: response_correct ~ logic_belief_consistent * (scale(log(rt)) +
    consistent_plottable) + (1 | syllogism_name)
   Data: syllogism_model_df %>% filter(subject == "Human")

     AIC      BIC   logLik deviance df.resid
   693.4    724.6   -339.7    679.4      631

Scaled residuals:
    Min      1Q  Median      3Q     Max
-4.5854 -0.6137  0.3423  0.6457  2.1597

Random effects:
 Groups         Name        Variance Std.Dev.
 syllogism_name (Intercept) 0.08975  0.2996
Number of obs: 638, groups:  syllogism_name, 12

Fixed effects:
                                                     Estimate Std. Error z value Pr(>|z|)
(Intercept)                                           0.61895    0.19140   3.234  0.00122 **
logic_belief_consistent1                              3.16927    0.33673   9.412  < 2e-16 ***
scale(log(rt))                                       -0.08297    0.09858  -0.842  0.39998
consistent_plottableViolate                           0.20829    0.21271   0.979  0.32746
logic_belief_consistent1:scale(log(rt))              -0.35038    0.19413  -1.805  0.07109 .
logic_belief_consistent1:consistent_plottableViolate -2.40845    0.42012  -5.733 9.88e-09 ***
```

Table S6: Mixed-effects regression examining the continuous effect of RT on the Syllogism tasks. There is no main effect, but there is a marginally-significant interaction with the content effect, such that slower responses are more helpful on problems where content contradicts logic.

B.8.1 NLI

First, for the NLI task, we plot the item-level correlations in accuracy in Fig. S22. Surprisingly (given the close-to-ceiling performance), we find that Human success rates are significantly predictive of LM success rates, even when controlling for condition (Table S7).

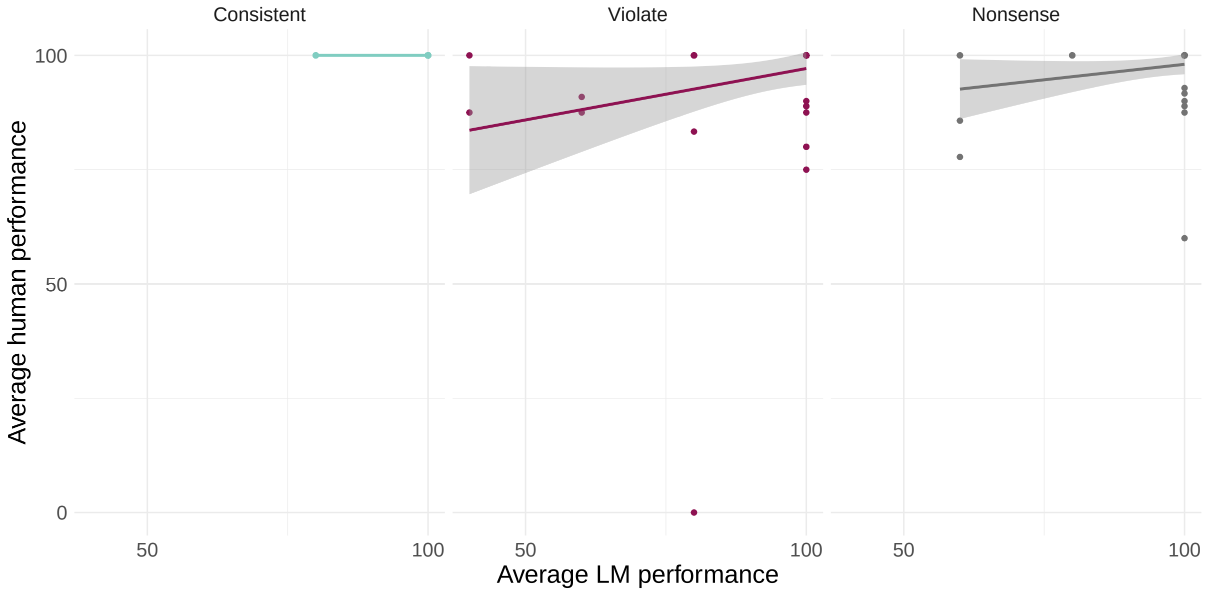

Figure S22: Association of human and average model accuracy on the NLI task.

```
Linear mixed model fit by REML ['lmerMod']
Formula: LM ~ Human + consistent_plottable + (1 | model)
   Data: nli_item_level_df

REML criterion at convergence: -404.8

Scaled residuals:
    Min      1Q  Median      3Q     Max
-5.2460  0.0661  0.1428  0.2724  1.6139

Random effects:
 Groups   Name        Variance  Std.Dev.
 model    (Intercept) 0.0008882 0.0298
 Residual             0.0350993 0.1873
Number of obs: 845, groups:  model, 5

Fixed effects:
                             Estimate Std. Error t value
(Intercept)                   0.73890    0.07288  10.139
Human                         0.24681    0.07077   3.488
consistent_plottableViolate  -0.02879    0.01560  -1.846
consistent_plottableNonsense -0.02429    0.01649  -1.473
```

Table S7: Mixed-effects linear regression for item-level association of human and model accuracy on the NLI task, controlling for consistency.

B.8.2 Syllogisms

For the Syllogisms task, we plot the item-level correlations in accuracy in Fig. S22. We again find a significant relationship between human success rates and language model success ($t = 4.98$, $p < 0.001$ when controlling for task variables; Table S8).

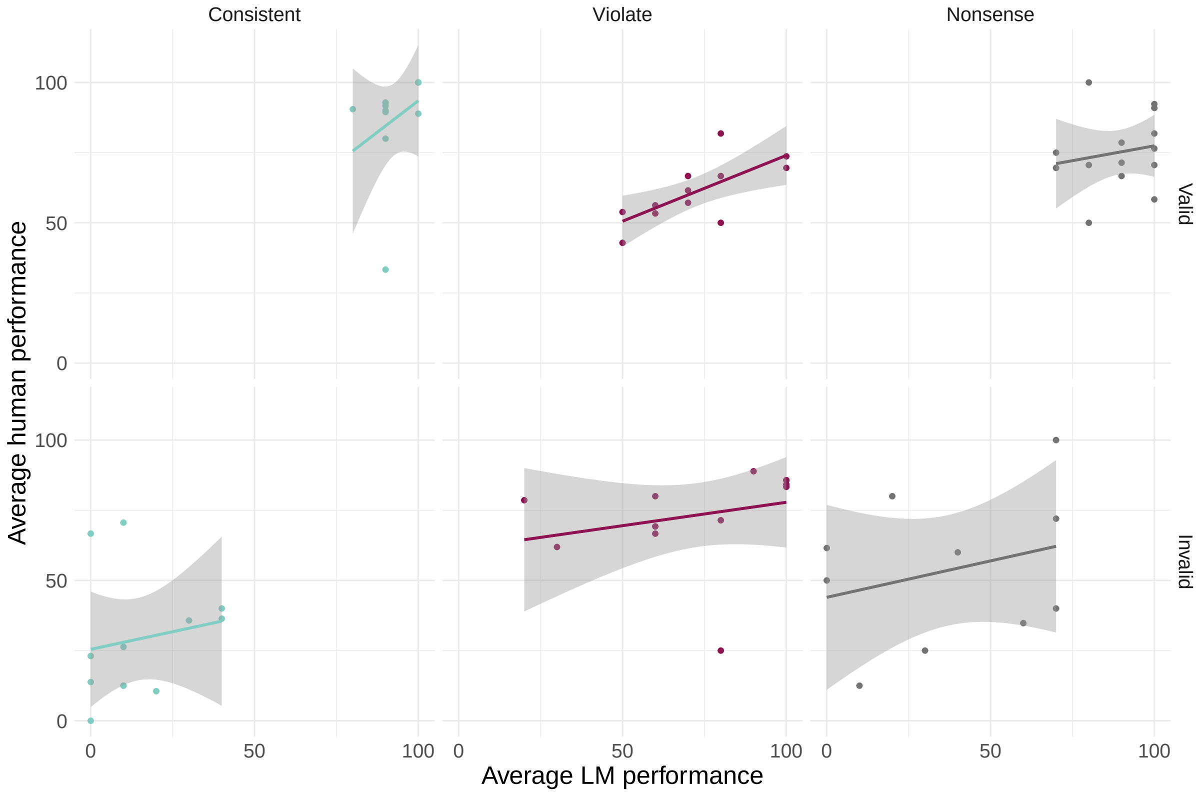

Figure S23: Association of human and average model accuracy on the Syllogisms task.

```
Linear mixed model fit by REML ['lmerMod']
Formula: LM ~ Human + logic_belief_consistent * consistent_plottable +
    (1 | model)
   Data: syl_item_level_df

REML criterion at convergence: 313.7

Scaled residuals:
    Min      1Q  Median      3Q     Max
-2.5622 -0.5048  0.2668  0.6984  2.8012

Random effects:
 Groups   Name        Variance Std.Dev.
 model    (Intercept) 0.004978 0.07056
 Residual             0.131391 0.36248
Number of obs: 355, groups:  model, 5

Fixed effects:
                                                     Estimate Std. Error t value
(Intercept)                                           0.23822    0.07538   3.160
Human                                                 0.51089    0.10262   4.978
logic_belief_consistent                               0.24473    0.04505   5.432
consistent_plottableViolate                           0.14737    0.04827   3.053
consistent_plottableNonsense                          0.09873    0.04806   2.054
logic_belief_consistent:consistent_plottableViolate -0.27191    0.05281  -5.148
```

Table S8: Mixed-effects linear regression for item-level association of human and model accuracy on the Syllogisms task, controlling for content and logic.

B.8.3 Wason

For the Wason task, we plot the item-level correlations in accuracy in Fig. S22. Perhaps because human performance is low overall, we do not observe a significant relationship between human success rates and language model success (Table S9).

Due to the item-level effects observed in some of the main regressions, we also plot performance of each model or human group on each of the Wason rules in Fig. S25. Overall, the variability seems mostly as expected. However, there are some interesting patterns, including one arbitrary rule that most subject perform well on. That particular rule is:

```
The rule is that if the cards have a French word then they must have a positive number.
chapeau / sombrero / 4 / -1
```

It is not particularly apparent to us why this rule might be easier.

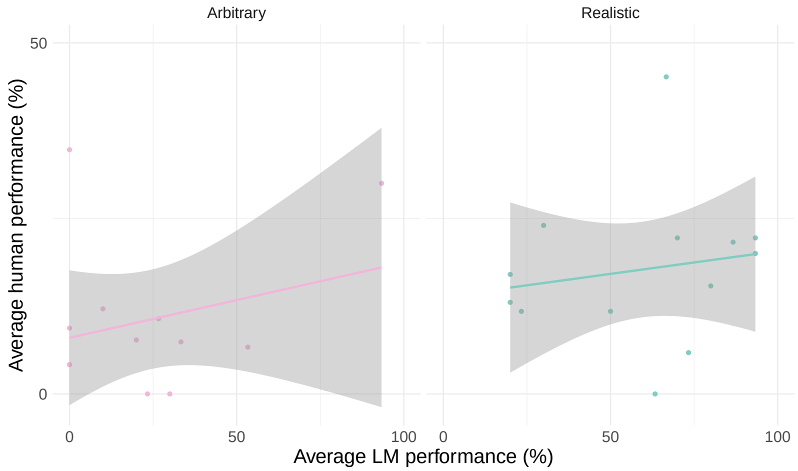

Figure S24: Association of human and average model accuracy on the Wason task. Note the vertical axis scale—human performance is low overall.

B.9 Visualizing the correlation of human response times with model log-probs

In Fig. S26 we show the relationship between human response times and model log-probabilities for each of the models we considered.

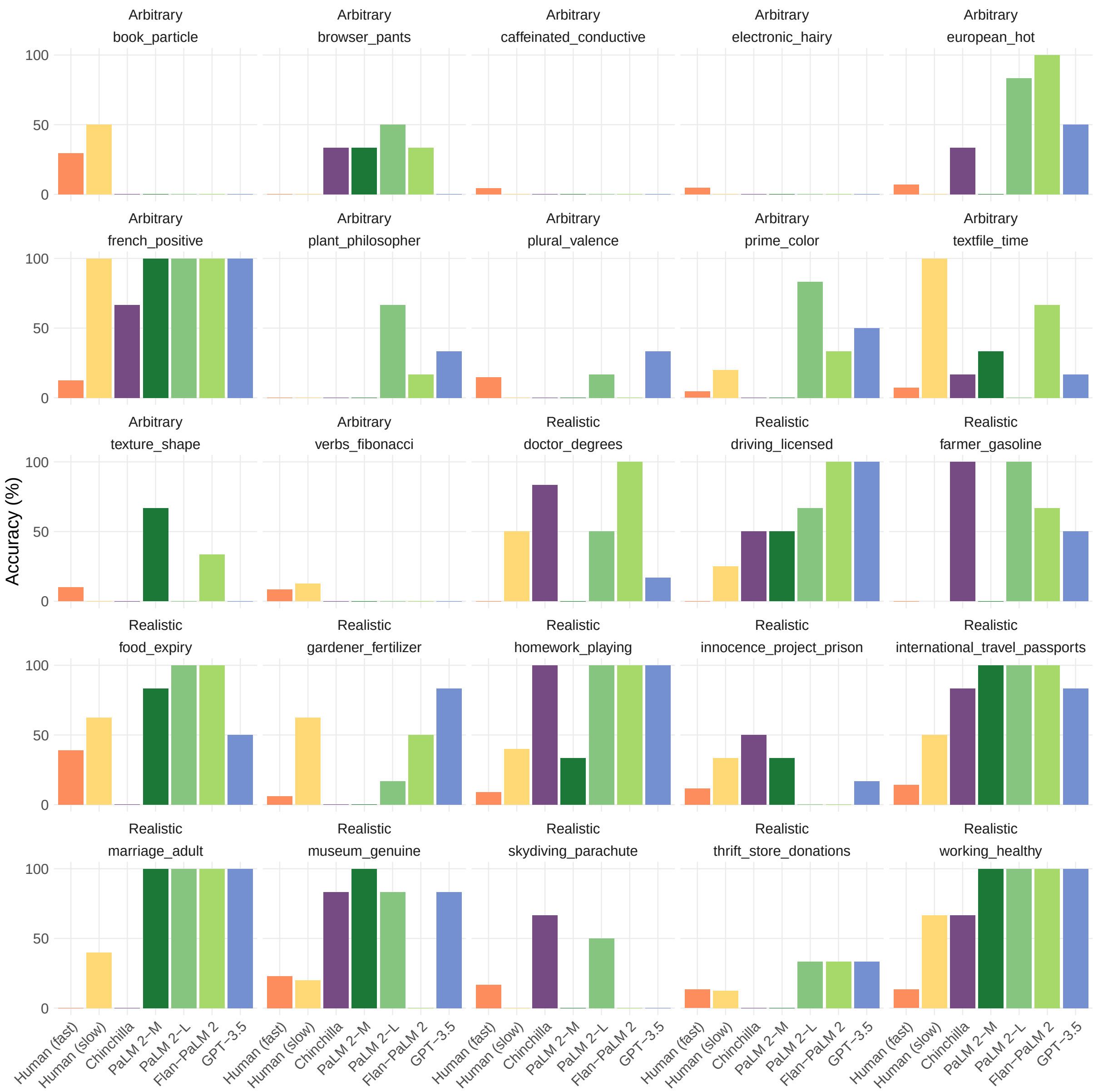

Figure S25: Accuracy of humans and each model on each rule for the Wason tasks. Note that due to sampling variability, the number of human participants who experienced each rule varies, particularly for the slow subjects. There are various suggestive patterns, including an arbitrary rule (`french_positive`) that models and slower humans perform quite well on, and realistic rules (like `skydiving_parachute`) that all perform surprisingly poorly on. (Note that the variation within a model comes from testing on multiple variations of each problem, with different card orders and card names; see SI A.1.)

```
Linear mixed model fit by REML ['lmerMod']
Formula: LM ~ Human + wason_condition + (1 | model)
   Data: wason_item_level_df

REML criterion at convergence: 181.3

Scaled residuals:
    Min      1Q  Median      3Q     Max
-1.7748 -0.7994 -0.1560  0.8760  1.9239

Random effects:
 Groups   Name        Variance Std.Dev.
 model    (Intercept) 0.006414 0.08009
 Residual             0.145352 0.38125
Number of obs: 185, groups:  model, 5

Fixed effects:
                         Estimate Std. Error t value
(Intercept)               0.20251    0.06860   2.952
Human                     0.36974    0.29860   1.238
wason_conditionRealistic  0.32436    0.07148   4.538
wason_conditionNonsense   0.19533    0.06967   2.804
```

Table S9: Mixed-effects linear regression for item-level association of human and model accuracy on the Syllogisms task, controlling for content and logic.

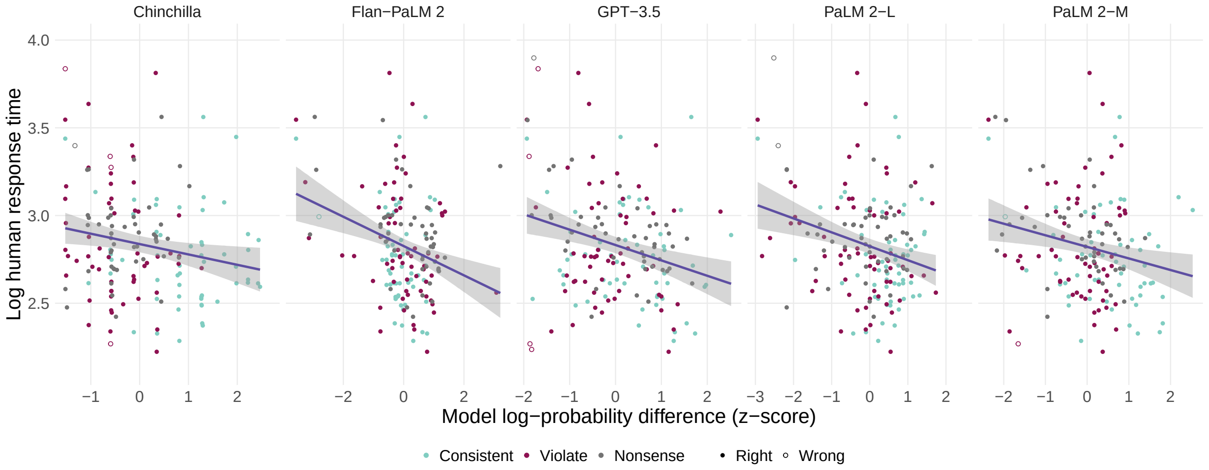

(a) Natural language inference raw results.

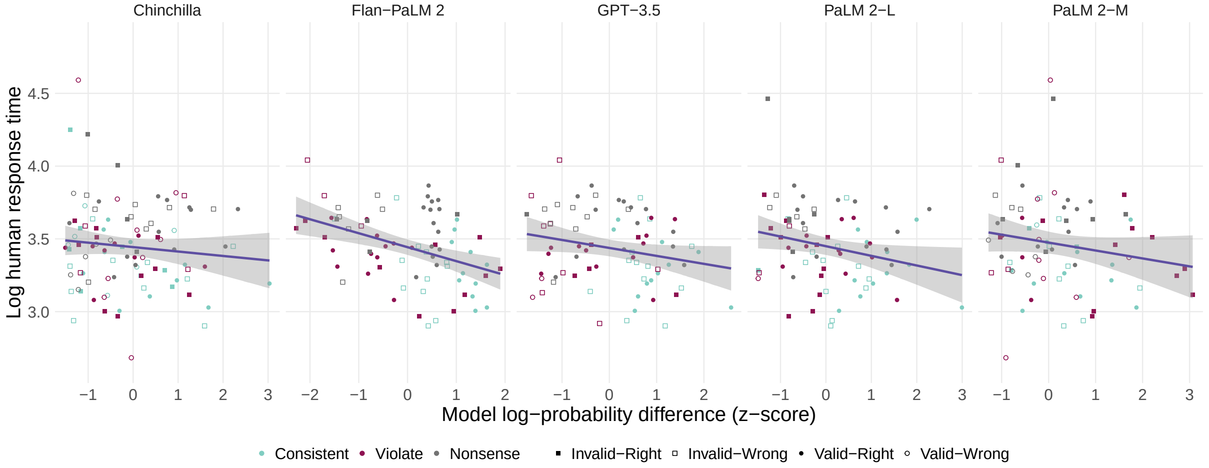

(b) Syllogisms raw results.

Figure S26: Human response times are generally negatively related to model confidence (measured as the difference in log-probabilities between the correct answer and the incorrect answer). That is, on problems for which the model displays greater confidence, humans tend to respond more quickly. This relationship holds on both (a) the NLI tasks, and (b) the syllogism tasks. (Points show average response times for individual problems, broken down by whether the humans or models answered correctly or not; see SI C.4 for details.)

B.10 Model answer log-probability distributions

In this section we plot the log-probability distributions of the models on the different tasks (Figs. S27, S28, S29). There are a variety of interesting effects of task variables, and some striking differences among the models.

For example, the instruction-tuned models (Flan-PaLM 2 and GPT-3.5) have numerically much greater magnitude log-probabilities to the answers, especially GPT-3.5. This may be an artifact of the tuning process. Furthermore, the larger models tend to show clearer separation between the chosen answer and the others (e.g., comparing PaLM 2-L to -M).

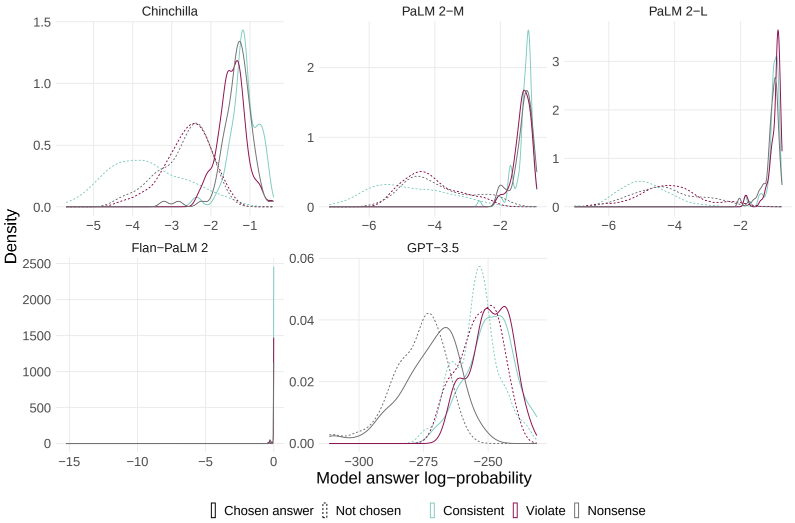

(a) Raw log-probabilities.

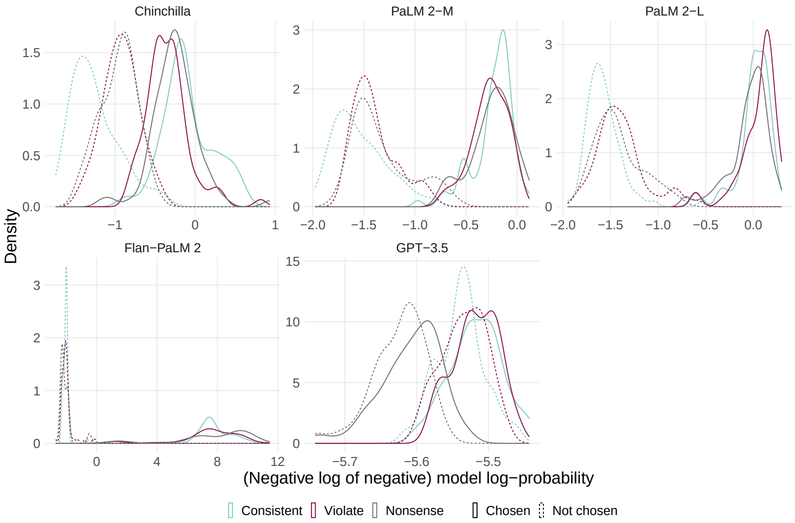

(b) Log transformed.

Figure S27: Model log-probability distributions for the answer choices on the Natural Language Inference (NLI) task. We visualize these in two ways: (a) the raw log-probabilities, and (a) the negative log of the negative log-probabilities — this transform makes the distribution for Flan-PaLM 2 clearer. Across both plots, there is fairly clear separation between the distributions of chosen and unchosen answers for most models. There are various interesting effects of content on the log-probabilities, e.g. changes in the mean and variance of the distributions. There are also striking differences among the models, possibly hinting at the effects of different training processes.

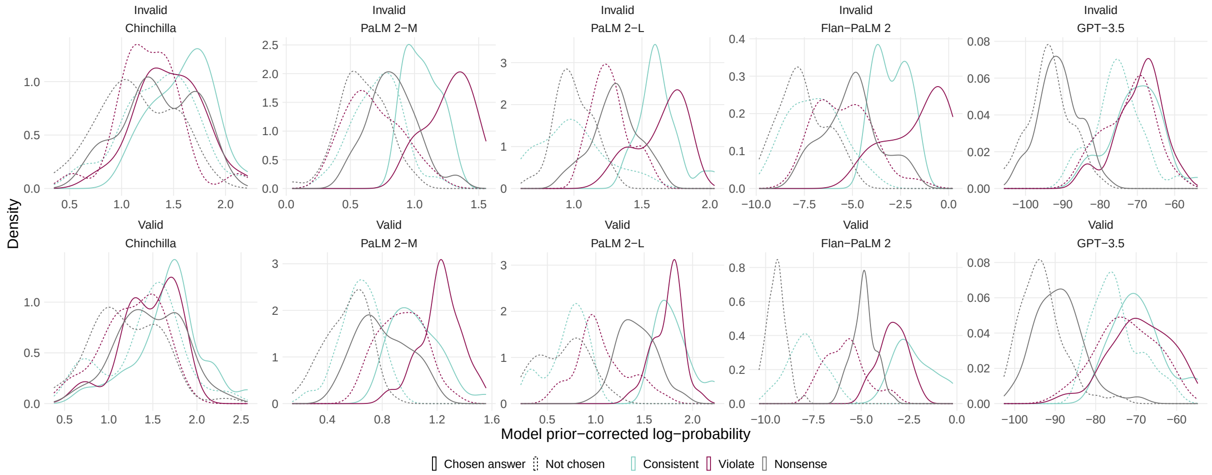

Figure S28: Model prior-corrected log-probability distributions for the answer choices on the syllogisms task. The degree of separation between the distributions depends on the model, validity, and content. Again, there are differences among the models. For example, larger models show more cleanly separated distributions (PaLM 2-L vs. -M), and the instruction tuned models (Flan-PaLM 2 and GPT-3.5) show much larger magnitude prior corrected log probabilities.

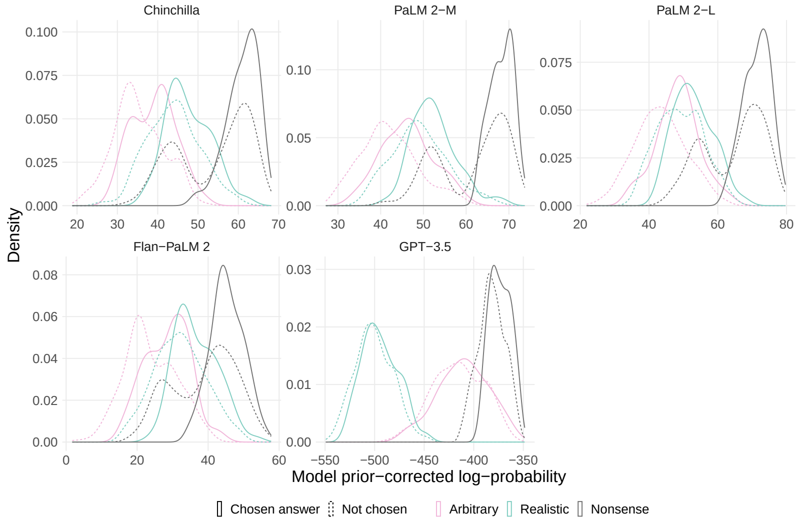

Figure S29: Model prior-corrected log-probability distributions for the answer choices on the Wason selection task. The degree of separation between the chosen and not-chosen answer distributions is generally lower than in other tasks, possibly reflecting the greater difficulty of the Wason task, or the greater problem-to-problem variability. By contrast, the separation by content is striking for some models, e.g. GPT-3.5.

B.11 Chinchilla can identify the valid conclusion of a syllogism from among all possible conclusions with high accuracy

In Fig. S30 we show the accuracy of Chinchilla when choosing from among all possible predicates containing one of the quantifiers used and two of the entities appearing in the premises of the syllogism. The model exhibits high accuracy across conditions, and relatively little bias (though bias increases few shot). This observation is reminiscent of the finding of Trippas et al. (54) that humans exhibit less bias when making a forced choice among two possible arguments (one valid and one invalid) rather than deciding if a single syllogism is valid or invalid.

Note that in this case scoring with the Domain-Conditional PMI (120)—which we used for the main Syllogisms and Wason results—produces much *lower* accuracy than the raw likelihoods, and minor differences in bias. The patterns are qualitatively similar with or without the correction, but accuracy is lower without (around 35-40%) regardless of belief consistency.

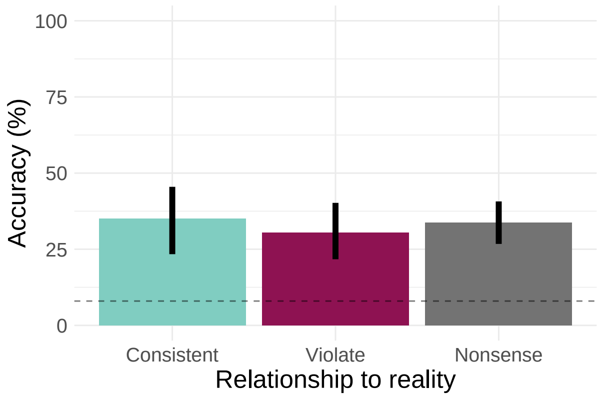

(a) Domain-conditional PMI scoring

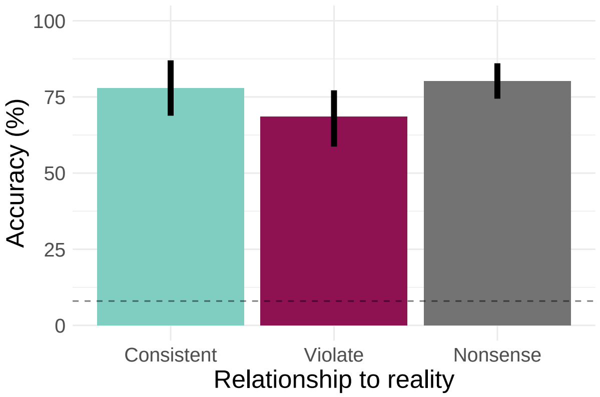

(b) Raw log-likelihood scoring

Figure S30: Chinchilla's zero-shot accuracy at identifying the correct conclusion to a syllogism among all possible conclusions. The model exhibits far above chance performance (especially when scoring with raw log-likelihoods), and relatively weaker bias with this task design.

C Statistical analyses

In this section, we provide the full results for all statistical analyses reported in the main text. We generally report results from mixed-effects logistic regressions, controlling for the random effects of the different stimuli used.[3]

C.1 NLI

We report statistical analyses of content effects on the NLI tasks for humans and all models in Tables S10-S15. We generally fit mixed effects logistic regressions, but the regressions for PaLM 2-L and Flan-PaLM 2 failed to converge due to ceiling effects. We therefore also report χ^2 tests of the difference in correct responses across conditions. In all cases, we do not find a significant content effect on this simple task.

```
Generalized linear mixed model fit by maximum likelihood (Laplace
  Approximation) [glmerMod]
 Family: binomial  ( logit )
Formula: response_correct ~ consistent_plottable + (1 | name)
   Data:
nli_joint_df %>% filter(subject == "Human", consistent_plottable !=
    "Nonsense")

     AIC      BIC   logLik deviance df.resid
   133.2    146.8    -63.6    127.2      677

Scaled residuals:
    Min      1Q  Median      3Q     Max
-3.5119  0.0105  0.0106  0.0282  0.4931

Random effects:
 Groups Name        Variance Std.Dev.
 name   (Intercept) 25.82    5.081
Number of obs: 680, groups:  name, 122

Fixed effects:
                            Estimate Std. Error z value Pr(>|z|)
(Intercept)                    9.082      2.072   4.384 1.17e-05 ***
consistent_plottableViolate   -2.051      1.608  -1.276    0.202
---
Signif. codes:  0 '***' 0.001 '**' 0.01 '*' 0.05 '.' 0.1 ' ' 1
```

(a) Mixed-effects logistic regression.

```
Chi-squared test for given probabilities
X-squared = 0.33937, df = 1, p-value = 0.5602
```

(b) χ^2 test.

Table S10: Statistical analyses of human performance on the NLI tasks, using (a) a mixed-effects logistic regression or (b) a χ^2 test. There are no significant content effects.

[3]Unless otherwise noted, we conservatively approximate the degrees of freedom for all t-tests by treating all random effects as though they were fixed effects (i.e. by subtracting the number of levels of each random variable from the residual degrees of freedom), rather than using a variance-based approximation.

```
Generalized linear mixed model fit by maximum likelihood (Laplace
  Approximation) [glmerMod]
 Family: binomial  ( logit )
Formula: response_correct ~ consistent_plottable + (1 | name)
   Data:
nli_joint_df %>% filter(subject == "Chinchilla", consistent_plottable !=
    "Nonsense")

     AIC      BIC   logLik deviance df.resid
    46.6     55.8    -20.3     40.6      153

Scaled residuals:
      Min        1Q    Median        3Q       Max
-0.084635  0.000969  0.000969  0.001903  0.001903

Random effects:
 Groups Name        Variance Std.Dev.
 name   (Intercept) 2646     51.43
Number of obs: 156, groups:  name, 153

Fixed effects:
                            Estimate Std. Error z value Pr(>|z|)
(Intercept)                   13.877      3.373   4.114  3.9e-05 ***
consistent_plottableViolate   -1.357      3.760  -0.361    0.718
---
Signif. codes:  0 '***' 0.001 '**' 0.01 '*' 0.05 '.' 0.1 ' ' 1
```

(a) Mixed-effects logistic regression.

```
Chi-squared test for given probabilities
X-squared = 0.34266, df = 1, p-value = 0.5583
```

(b) χ^2 test.

Table S11: Statistical analyses of Chinchilla's performance on the NLI tasks, using (a) a mixed-effects logistic regression or (b) a χ^2 test. There are no significant content effects.

```
Generalized linear mixed model fit by maximum likelihood (Laplace
  Approximation) [glmerMod]
 Family: binomial  ( logit )
Formula: response_correct ~ consistent_plottable + (1 | name)
   Data:
nli_joint_df %>% filter(subject == "PaLM 2-M", consistent_plottable !=
    "Nonsense")

     AIC      BIC   logLik deviance df.resid
    22.0     31.1     -8.0     16.0      153

Scaled residuals:
      Min        1Q    Median        3Q       Max
-0.074976  0.000648  0.000648  0.000804  0.000804

Random effects:
 Groups Name        Variance Std.Dev.
 name   (Intercept) 3553     59.61
Number of obs: 156, groups:  name, 153

Fixed effects:
                            Estimate Std. Error z value Pr(>|z|)
(Intercept)                  14.2497     3.5260   4.041 5.31e-05 ***
consistent_plottableViolate   0.4323     5.5651   0.078    0.938
---
Signif. codes:  0 '***' 0.001 '**' 0.01 '*' 0.05 '.' 0.1 ' ' 1

Correlation of Fixed Effects:
           (Intr)
cnsstnt_plV -0.629
```

(a) Mixed-effects logistic regression.

```
Chi-squared test for given probabilities
X-squared = 0.0066225, df = 1, p-value = 0.9351
```

(b) χ^2 test.

Table S12: Statistical analyses of PaLM 2-M's performance on the NLI tasks, using (a) a mixed-effects logistic regression or (b) a χ^2 test. There are no significant content effects.

```
Chi-squared test for given probabilities
X-squared = 0, df = 1, p-value = 1
```

Table S13: Statistical analysis of PaLM 2-L's performance on the NLI tasks, using a χ^2 test, as the logistic regression failed to converge. There are no significant content effects.

```
Chi-squared test for given probabilities
X-squared = 0.0064516, df = 1, p-value = 0.936
```

Table S14: Statistical analysis of Flan-PaLM 2's performance on the NLI tasks, using a χ^2 test, as the logistic regression failed to converge. There are no significant content effects.

```
Generalized linear mixed model fit by maximum likelihood (Laplace
  Approximation) [glmerMod]
 Family: binomial  ( logit )
Formula: response_correct ~ consistent_plottable + (1 | name)
   Data:
nli_joint_df %>% filter(subject == "GPT-3.5", consistent_plottable !=
    "Nonsense")

     AIC      BIC   logLik deviance df.resid
    31.3     40.5    -12.7     25.3      153

Scaled residuals:
      Min        1Q    Median        3Q       Max
-0.078288  0.000867  0.000867  0.001145  0.001145

Random effects:
 Groups Name        Variance Std.Dev.
 name   (Intercept) 3151     56.13
Number of obs: 156, groups:  name, 153

Fixed effects:
                            Estimate Std. Error z value Pr(>|z|)
(Intercept)                  14.0988     3.3443   4.216 2.49e-05 ***
consistent_plottableViolate  -0.5577     4.1579  -0.134    0.893
---
Signif. codes:  0 '***' 0.001 '**' 0.01 '*' 0.05 '.' 0.1 ' ' 1
```

(a) Mixed-effects logistic regression.

```
Chi-squared test for given probabilities
X-squared = 0.027027, df = 1, p-value = 0.8694
```

(b) χ^2 test.

Table S15: Statistical analyses of GPT-3.5-turbo-instruct's performance on the NLI tasks, using (a) a mixed-effects logistic regression or (b) a χ^2 test. There are no significant content effects.

C.2 Syllogisms

We report mixed effects logistic regressions for humans and all models in Tables S16-S21. We analyze these results using a variable which corresponds to the main content effect (`logic_belief_consistent`), which is 1 when the logical answer matches the believability of the conclusion — i.e. when the argument is valid and the conclusion is believable, or the argument is invalid and the conclusion is unbelievable — and 0 when there is a mismatch. This measure corresponds to the difference score reported in Fig. 2b. We ran three nested models for humans and each language model — one regression only incorporating the content effect predictor (whether the logic matches the consistency), another adding consistency condition, and a third adding the interaction of the two.

```
response_correct ~ logic_belief_consistent + (1 | syllogism_name)
response_correct ~ logic_belief_consistent + consistent_plottable_f + (1 | syllogism_name)
response_correct ~ logic_belief_consistent * consistent_plottable_f + (1 | syllogism_name)
```

For humans and each language model, we report the best-fitting regression, measured by the BIC (and omitting models which failed to converge). However, since the interaction effect is theoretically interesting (e.g. 53), and several of the interaction models fail to converge, we also report two-way χ^2 tests of the interactions for each model. For PaLM 2-L all regressions failed to converge due to ceiling effects; thus we also report a χ^2 test of the content effect for this model only. All models show a significant content effect; all except Chinchilla and PaLM 2-M show a significant interaction.

```
Generalized linear mixed model fit by maximum likelihood (Laplace
  Approximation) [glmerMod]
 Family: binomial  ( logit )
Formula: response_correct ~ logic_belief_consistent * consistent_plottable_f +
    (1 | syllogism_name)
   Data: syllogism_model_df %>% filter(subject == this_subject)

     AIC      BIC   logLik deviance df.resid
   692.8    715.1   -341.4    682.8      633

Scaled residuals:
    Min      1Q  Median      3Q     Max
-3.7130 -0.6097  0.3328  0.6018  1.9316

Random effects:
 Groups         Name        Variance Std.Dev.
 syllogism_name (Intercept) 0.08992  0.2999
Number of obs: 638, groups:  syllogism_name, 12

Fixed effects:
                                                 Estimate Std. Error z value Pr(>|z|)
(Intercept)                                        0.7182     0.1359   5.286 1.25e-07 ***
logic_belief_consistent1                           1.9502     0.2081   9.372  < 2e-16 ***
consistent_plottable_f1                            0.1863     0.2114   0.881    0.378
logic_belief_consistent1:consistent_plottable_f1  -2.4800     0.4172  -5.945 2.76e-09 ***
---
Signif. codes:  0 '***' 0.001 '**' 0.01 '*' 0.05 '.' 0.1 ' ' 1
```

(a) Mixed-effects logistic regression.

```
Pearson's Chi-squared test with Yates' continuity correction
X-squared = 11.402, df = 1, p-value = 0.0007338
```

(b) χ^2 test of interaction.

Table S16: Statistical analyses of human performance on the Syllogism tasks, using (a) a mixed-effects logistic regression or (b) a χ^2 test of the interaction effect. There is a significant content effect and a significant interaction effect, that is, different sensitivity to logic in the Consistent compared to Violate conditions.

```
Generalized linear mixed model fit by maximum likelihood (Laplace
  Approximation) [glmerMod]
 Family: binomial  ( logit )
Formula: response_correct ~ logic_belief_consistent + (1 | syllogism_name)
   Data: syllogism_model_df %>% filter(subject == this_subject)

     AIC      BIC   logLik deviance df.resid
   125.8    133.5    -59.9    119.8       93

Scaled residuals:
    Min      1Q  Median      3Q     Max
-1.7321 -0.8819  0.5774  0.5774  1.1339

Random effects:
 Groups         Name        Variance Std.Dev.
 syllogism_name (Intercept) 0        0
Number of obs: 96, groups:  syllogism_name, 12

Fixed effects:
                         Estimate Std. Error z value Pr(>|z|)
(Intercept)                0.4236     0.2212   1.915  0.05549 .
logic_belief_consistent1   1.3499     0.4425   3.051  0.00228 **
---
Signif. codes:  0 '***' 0.001 '**' 0.01 '*' 0.05 '.' 0.1 ' ' 1
```

(a) Mixed-effects logistic regression.

```
Pearson's Chi-squared test with Yates' continuity correction
X-squared = 6.0122e-31, df = 1, p-value = 1
```

(b) χ^2 test of interaction.

Table S17: Statistical analyses of Chinchilla's performance on the Syllogism tasks, using (a) a mixed-effects logistic regression or (b) a χ^2 test of the interaction effect. Chinchilla shows significant content effects, but no interaction with consistency.

```
Generalized linear mixed model fit by maximum likelihood (Laplace
  Approximation) [glmerMod]
 Family: binomial  ( logit )
Formula: response_correct ~ logic_belief_consistent + (1 | syllogism_name)
   Data: syllogism_model_df %>% filter(subject == this_subject)

     AIC      BIC   logLik deviance df.resid
   102.0    109.6    -48.0     96.0       93

Scaled residuals:
    Min      1Q  Median      3Q     Max
-2.4202 -0.6095  0.4132  0.4132  1.6408

Random effects:
 Groups         Name        Variance Std.Dev.
 syllogism_name (Intercept) 0        0
Number of obs: 96, groups:  syllogism_name, 12

Fixed effects:
                         Estimate Std. Error z value Pr(>|z|)
(Intercept)                0.3886     0.2611   1.488    0.137
logic_belief_consistent1   2.7581     0.5222   5.281 1.28e-07 ***
---
Signif. codes:  0 '***' 0.001 '**' 0.01 '*' 0.05 '.' 0.1 ' ' 1
```

(a) Mixed-effects logistic regression.

```
Pearson's Chi-squared test with Yates' continuity correction
X-squared = 0, df = 1, p-value = 1
```

(b) χ^2 test of interaction.

Table S18: Statistical analyses of PaLM 2-M's performance on the Syllogism tasks, using (a) a mixed-effects logistic regression or (b) a χ^2 test. PaLM 2-M shows significant content effects, but no interaction with consistency.

```
Chi-squared test for given probabilities
X-squared = 6.3913, df = 1, p-value = 0.01147
```

(a) χ^2 test of content effect.

```
Pearson's Chi-squared test with Yates' continuity correction
X-squared = 14.318, df = 1, p-value = 0.0001544
```

(b) χ^2 test of interaction.

Table S19: Statistical analyses of PaLM 2-L's performance on the Syllogism tasks, using (a) a χ^2 test of the content effect as none of the regressions converged, and (b) a χ^2 test of the interaction. PaLM 2-L shows both significant content effects, and a significant interaction with consistency (as measured by the χ^2 test, as the regression with an interaction failed to converge).

```
Generalized linear mixed model fit by maximum likelihood (Laplace
  Approximation) [glmerMod]
 Family: binomial  ( logit )
Formula: response_correct ~ logic_belief_consistent + (1 | syllogism_name)
   Data: syllogism_model_df %>% filter(subject == this_subject)

     AIC      BIC   logLik deviance df.resid
   100.1    107.8    -47.0     94.1       93

Scaled residuals:
    Min      1Q  Median      3Q     Max
-3.3166 -1.0000  0.3015  0.4761  1.0000

Random effects:
 Groups         Name        Variance Std.Dev.
 syllogism_name (Intercept) 0        0
Number of obs: 96, groups:  syllogism_name, 12

Fixed effects:
                         Estimate Std. Error z value Pr(>|z|)
(Intercept)                1.1989     0.2984   4.019 5.86e-05 ***
logic_belief_consistent1   2.3979     0.5967   4.019 5.86e-05 ***
---
Signif. codes:  0 '***' 0.001 '**' 0.01 '*' 0.05 '.' 0.1 ' ' 1
```

(a) Mixed-effects logistic regression.

```
Pearson's Chi-squared test with Yates' continuity correction
X-squared = 17.913, df = 1, p-value = 2.312e-05
```

(b) χ^2 test of interaction.

Table S20: Statistical analyses of Flan-PaLM 2's performance on the Syllogism tasks, using (a) a mixed-effects logistic regression or (b) a χ^2 test. Flan-PaLM 2 shows both significant content effects, and a significant interaction with consistency (as measured by the χ^2 test, as the regression with an interaction failed to converge).

```
Generalized linear mixed model fit by maximum likelihood (Laplace
  Approximation) [glmerMod]
 Family: binomial  ( logit )
Formula: response_correct ~ logic_belief_consistent + (1 | syllogism_name)
   Data: syllogism_model_df %>% filter(subject == this_subject)

     AIC      BIC   logLik deviance df.resid
   131.8    139.5    -62.9    125.8       93

Scaled residuals:
    Min      1Q  Median      3Q     Max
-1.4832 -0.9199  0.6742  0.6742  1.0871

Random effects:
 Groups         Name        Variance Std.Dev.
 syllogism_name (Intercept) 0        0
Number of obs: 96, groups:  syllogism_name, 12

Fixed effects:
                         Estimate Std. Error z value Pr(>|z|)
(Intercept)                0.3107     0.2127   1.461   0.1440
logic_belief_consistent1   0.9555     0.4253   2.247   0.0247 *
---
Signif. codes:  0 '***' 0.001 '**' 0.01 '*' 0.05 '.' 0.1 ' ' 1
```

(a) Mixed-effects logistic regression.

```
Pearson's Chi-squared test with Yates' continuity correction
X-squared = 25.507, df = 1, p-value = 4.408e-07
```

(b) χ^2 test.

Table S21: Statistical analyses of GPT-3.5-turbo-instruct's performance on the syl tasks, using (a) a mixed-effects logistic regression or (b) a χ^2 test. GPT-3.5 shows both significant content effects, and a significant interaction with consistency (as measured by the χ^2 test, as the regression with an interaction failed to converge).

C.3 Wason

We report mixed-effects logistic regressions for humans (both all humans, and the fast and slow groups individually) and all models in Tables S22-S29. We observe a significant effect of content in most cases. However, the fast humans alone do not show a significant content effect. Furthermore, the content effects in PaLM 2-M and Flan-PaLM 2 are only marginally significant, due to high item level variance.

In SI C.3.1 we further analyze the human data whil incorporating response time in the regression.

```
Generalized linear mixed model fit by maximum likelihood (Laplace
  Approximation) [glmerMod]
 Family: binomial  ( logit )
Formula: response_correct ~ wason_condition + (1 | wason_name)
   Data: wason_joint_df %>% filter(subject_no_rt == "Human", wason_condition %in%
    c("Arbitrary", "Realistic"))

     AIC      BIC   logLik deviance df.resid
     478      491     -236      472      571

Scaled residuals:
    Min      1Q  Median      3Q     Max
-0.7162 -0.4629 -0.3279 -0.2881  3.4711

Random effects:
 Groups     Name        Variance Std.Dev.
 wason_name (Intercept) 0.2309   0.4805
Number of obs: 574, groups:  wason_name, 25

Fixed effects:
                         Estimate Std. Error z value Pr(>|z|)
(Intercept)               -2.2951     0.2554  -8.988   <2e-16 ***
wason_conditionRealistic   0.8219     0.3235   2.541   0.0111 *
---
Signif. codes:  0 '***' 0.001 '**' 0.01 '*' 0.05 '.' 0.1 ' ' 1
```

Table S22: Statistical analysis of human performance (collapsing across fast and slow subjects) on the Wason tasks, using a logistic regression. There is a significant content effect.

```
Generalized linear mixed model fit by maximum likelihood (Laplace
  Approximation) [glmerMod]
 Family: binomial  ( logit )
Formula: response_correct ~ wason_condition + (1 | wason_name)
   Data:
wason_joint_df %>% filter(subject == this_subject, wason_condition %in%
    c("Arbitrary", "Realistic"))

     AIC      BIC   logLik deviance df.resid
   305.1    317.4   -149.6    299.1      442

Scaled residuals:
    Min      1Q  Median      3Q     Max
-0.5732 -0.3620 -0.3034 -0.2709  3.7157

Random effects:
 Groups     Name        Variance Std.Dev.
 wason_name (Intercept) 0.273    0.5225
Number of obs: 445, groups:  wason_name, 25

Fixed effects:
                         Estimate Std. Error z value Pr(>|z|)
(Intercept)               -2.4740     0.2948  -8.393   <2e-16 ***
wason_conditionRealistic   0.4570     0.3877   1.179    0.239
---
Signif. codes:  0 '***' 0.001 '**' 0.01 '*' 0.05 '.' 0.1 ' ' 1
```

Table S23: Statistical analysis of human (fast subjects only) performance on the Wason tasks, using a logistic regression. We do not observe a significant content effect

```
Generalized linear mixed model fit by maximum likelihood (Laplace
  Approximation) [glmerMod]
 Family: binomial  ( logit )
Formula: response_correct ~ wason_condition + (1 | wason_name)
   Data:
wason_joint_df %>% filter(subject == this_subject, wason_condition %in%
    c("Arbitrary", "Realistic"))

     AIC      BIC   logLik deviance df.resid
   156.8    165.4    -75.4    150.8      126

Scaled residuals:
    Min      1Q  Median      3Q     Max
-0.8934 -0.7468 -0.4277  1.1193  2.3380

Random effects:
 Groups     Name        Variance Std.Dev.
 wason_name (Intercept) 0.1906   0.4365
Number of obs: 129, groups:  wason_name, 24

Fixed effects:
                         Estimate Std. Error z value Pr(>|z|)
(Intercept)               -1.6534     0.4303  -3.842 0.000122 ***
wason_conditionRealistic   1.1518     0.5009   2.299 0.021495 *
---
Signif. codes:  0 '***' 0.001 '**' 0.01 '*' 0.05 '.' 0.1 ' ' 1
```

Table S24: Statistical analysis of human (slow) performance on the Wason tasks, using a logistic regression. There is a significant content effect.

```
Generalized linear mixed model fit by maximum likelihood (Laplace
  Approximation) [glmerMod]
 Family: binomial  ( logit )
Formula: response_correct ~ wason_condition + (1 | wason_name)
   Data:
wason_joint_df %>% filter(subject == this_subject, wason_condition %in%
    c("Arbitrary", "Realistic"))

     AIC      BIC   logLik deviance df.resid
   136.9    146.0    -65.5    130.9      147

Scaled residuals:
    Min      1Q  Median      3Q     Max
-1.9739 -0.2756 -0.1256  0.4490  2.6782

Random effects:
 Groups     Name        Variance Std.Dev.
 wason_name (Intercept) 6.068    2.463
Number of obs: 150, groups:  wason_name, 25

Fixed effects:
                         Estimate Std. Error z value Pr(>|z|)
(Intercept)                -3.584      1.154  -3.106  0.00190 **
wason_conditionRealistic    3.576      1.354   2.640  0.00828 **
---
Signif. codes:  0 '***' 0.001 '**' 0.01 '*' 0.05 '.' 0.1 ' ' 1
```

Table S25: Statistical analysis of Chinchilla's performance on the Wason tasks, using a logistic regression. There is a significant content effect.

```
Generalized linear mixed model fit by maximum likelihood (Laplace
  Approximation) [glmerMod]
 Family: binomial  ( logit )
Formula: response_correct ~ wason_condition + (1 | wason_name)
   Data:
wason_joint_df %>% filter(subject == this_subject, wason_condition %in%
    c("Arbitrary", "Realistic"))

     AIC      BIC   logLik deviance df.resid
   115.5    124.6    -54.8    109.5      147

Scaled residuals:
     Min       1Q   Median       3Q      Max
-2.14109 -0.13581 -0.04492  0.15813  1.50757

Random effects:
 Groups     Name        Variance Std.Dev.
 wason_name (Intercept) 30.12    5.488
Number of obs: 150, groups:  wason_name, 25

Fixed effects:
                         Estimate Std. Error z value Pr(>|z|)
(Intercept)                -5.842      2.296  -2.544   0.0110 *
wason_conditionRealistic    5.122      2.777   1.844   0.0651 .
---
Signif. codes:  0 '***' 0.001 '**' 0.01 '*' 0.05 '.' 0.1 ' ' 1
```

Table S26: Statistical analysis of PaLM 2-M's performance on the Wason tasks, using a logistic regression. There is a marginally-significant content effect, due to high item-level variance.

```
Generalized linear mixed model fit by maximum likelihood (Laplace
  Approximation) [glmerMod]
 Family: binomial  ( logit )
Formula: response_correct ~ wason_condition + (1 | wason_name)
   Data:
wason_joint_df %>% filter(subject == this_subject, wason_condition %in%
    c("Arbitrary", "Realistic"))

     AIC      BIC   logLik deviance df.resid
   142.6    151.6    -68.3    136.6      147

Scaled residuals:
    Min      1Q  Median      3Q     Max
-2.2846 -0.1708  0.1686  0.2894  2.2759

Random effects:
 Groups     Name        Variance Std.Dev.
 wason_name (Intercept) 9.488    3.08
Number of obs: 150, groups:  wason_name, 25

Fixed effects:
                         Estimate Std. Error z value Pr(>|z|)
(Intercept)                -1.921      1.182  -1.625   0.1043
wason_conditionRealistic    3.908      1.759   2.222   0.0263 *
---
Signif. codes:  0 '***' 0.001 '**' 0.01 '*' 0.05 '.' 0.1 ' ' 1
```

Table S27: Statistical analysis of PaLM 2-L's performance on the Wason tasks, using a logistic regression. There is a significant content effect.

```
Generalized linear mixed model fit by maximum likelihood (Laplace
  Approximation) [glmerMod]
 Family: binomial  ( logit )
Formula: response_correct ~ wason_condition + (1 | wason_name)
   Data:
wason_joint_df %>% filter(subject == this_subject, wason_condition %in%
    c("Arbitrary", "Realistic"))

     AIC      BIC   logLik deviance df.resid
   129.1    138.1    -61.6    123.1      147

Scaled residuals:
    Min      1Q  Median      3Q     Max
-1.4634 -0.2275 -0.1330  0.1271  2.2738

Random effects:
 Groups     Name        Variance Std.Dev.
 wason_name (Intercept) 18.14    4.259
Number of obs: 150, groups:  wason_name, 25

Fixed effects:
                         Estimate Std. Error z value Pr(>|z|)
(Intercept)                -2.143      1.591  -1.347   0.1778
wason_conditionRealistic    4.539      2.551   1.779   0.0752 .
---
Signif. codes:  0 '***' 0.001 '**' 0.01 '*' 0.05 '.' 0.1 ' ' 1
```

Table S28: Statistical analysis of Flan-PaLM 2's performance on the Wason tasks, using a logistic regression. There is a marginally-significant content effect, due to high item-level variance.

```
Generalized linear mixed model fit by maximum likelihood (Laplace
  Approximation) [glmerMod]
 Family: binomial  ( logit )
Formula: response_correct ~ wason_condition + (1 | wason_name)
   Data:
wason_joint_df %>% filter(subject == this_subject, wason_condition %in%
    c("Arbitrary", "Realistic"))

     AIC      BIC   logLik deviance df.resid
   154.3    163.3    -74.1    148.3      147

Scaled residuals:
    Min      1Q  Median      3Q     Max
-2.1094 -0.4257 -0.1980  0.3909  2.3489

Random effects:
 Groups     Name        Variance Std.Dev.
 wason_name (Intercept) 5.008    2.238
Number of obs: 150, groups:  wason_name, 25

Fixed effects:
                         Estimate Std. Error z value Pr(>|z|)
(Intercept)                -2.105      0.867  -2.428  0.01517 *
wason_conditionRealistic    3.092      1.188   2.603  0.00923 **
---
Signif. codes:  0 '***' 0.001 '**' 0.01 '*' 0.05 '.' 0.1 ' ' 1
```

Table S29: Statistical analysis of GPT-3.5-turbo-instruct's performance on the Wason tasks, using a logistic regression. There is a significant content effect.

C.3.1 Human analyses incorporating response time

Here we present two regression analyses of the human results that incorporate the response time. In Table S30 we show a mixed-effects logistic regression controlling for log response time; the content effect remains significant. Thus, the content effects are not solely driven by the differences in response time noted above (SI S19).

However, it is also possible to conceive of the shift in response time as *a part of* the content effect. We can analyze the data this way by z-scoring response time within each condition; thus, the effect of the mean difference in response time will be included in the condition predictor. We present these results in Table S31. Both content and z-scored response time remain significant predictors of success.

```
Generalized linear mixed model fit by maximum likelihood (Laplace
  Approximation) [glmerMod]
 Family: binomial  ( logit )
Formula: response_correct ~ wason_condition + scale(log(rt)) + (1 | wason_name)
   Data: wason_human_correct_df

     AIC      BIC   logLik deviance df.resid
   459.5    476.9   -225.8    451.5      570

Scaled residuals:
    Min      1Q  Median      3Q     Max
-1.0996 -0.4417 -0.3265 -0.2246  4.5293

Random effects:
 Groups     Name        Variance Std.Dev.
 wason_name (Intercept) 0.2539   0.5039
Number of obs: 574, groups:  wason_name, 25

Fixed effects:
                         Estimate Std. Error z value Pr(>|z|)
(Intercept)               -2.3248     0.2655  -8.755  < 2e-16 ***
wason_conditionRealistic   0.6659     0.3350   1.988   0.0468 *
scale(log(rt))             0.5637     0.1269   4.442 8.89e-06 ***
---
Signif. codes:  0 '***' 0.001 '**' 0.01 '*' 0.05 '.' 0.1 ' ' 1
```

Table S30: Statistical analysis of human (both fast and slow) performance on the Wason tasks, using a logistic regression and also controlling for (log) response time. The content effect remains significant.

```
Generalized linear mixed model fit by maximum likelihood (Laplace
  Approximation) [glmerMod]
 Family: binomial  ( logit )
Formula: response_correct ~ wason_condition + zscored_rt_by_condition +
    (1 | wason_name)
   Data: wason_human_correct_df

     AIC      BIC   logLik deviance df.resid
   459.5    476.9   -225.8    451.5      570

Scaled residuals:
    Min      1Q  Median      3Q     Max
-1.1002 -0.4417 -0.3265 -0.2247  4.5269

Random effects:
 Groups     Name        Variance Std.Dev.
 wason_name (Intercept) 0.254    0.504
Number of obs: 574, groups:  wason_name, 25

Fixed effects:
                         Estimate Std. Error z value Pr(>|z|)
(Intercept)               -2.4329     0.2700  -9.011  < 2e-16 ***
wason_conditionRealistic   0.8793     0.3349   2.626  0.00865 **
zscored_rt_by_condition    0.5540     0.1247   4.443 8.87e-06 ***
---
Signif. codes:  0 '***' 0.001 '**' 0.01 '*' 0.05 '.' 0.1 ' ' 1
```

Table S31: Statistical analysis of human (both fast and slow) performance on the Wason tasks, using a logistic regression and also controlling for (log) response time, but z-scored *within* condition. Again the content effect is significant.

C.3.2 Multinomial regression of the response patterns on the Wason tasks

In Table S32 we present the results of a multinomial logistic regression predicting which of the six possible subsets of answers the humans and language models chose on the Wason task. This regression quantitatively supports the claim that the behavior is nonrandom, and more generally quantifies the qualitative observations of response patterns made in the main text.

A matrix: 5 × 9 of type dbl

	(Intercept)	Realist	Nonsense	humanslow	chinchi	palm2_m	palm2_l	flanpalm2	gpt3.5
AT,CF (correct)	-2.64	1.43	0.75	1.20	2.31	2.31	3.17	3.19	2.34
AT,AF	-1.24	-1.35	-2.16	0.32	0.08	-0.21	-0.06	0.53	-0.59
AF,CT	-2.65	0.06	0.32	0.02	2.75	2.81	2.72	2.29	2.29
AF,CF	-2.45	0.64	0.21	0.22	2.31	1.38	2.05	2.27	1.13
CT,CF	-2.47	0.30	-1.07	0.97	-1.16	-0.60	-12.95	-12.33	-13.45

(a) Coefficients.

A matrix: 5 × 9 of type dbl

	(Intercept)	Realist	Nonsense	humanslow	chinchi	palm2_m	palm2_l	flanpalm2	gpt3.5
AT,CF (correct)	0.18	0.17	0.17	0.25	0.24	0.23	0.25	0.25	0.22
AT,AF	0.16	0.29	0.44	0.35	0.44	0.46	0.51	0.41	0.49
AF,CT	0.22	0.20	0.19	0.50	0.28	0.27	0.30	0.33	0.28
AF,CF	0.20	0.20	0.21	0.38	0.27	0.30	0.31	0.29	0.31
CT,CF	0.26	0.33	0.56	0.35	1.03	0.75	0.00	306.59	0.00

(b) Standard errors.

Table S32: Results of a multinomial logistic regression predicting the answer choices (reference level is AT,CT — the matching bias) from participants and language models based on condition (reference level is the Arbitrary condition), and participant group (reference level is fast humans). The regression was performed with dummy coding, so coefficients represent the difference in log odds relative to the reference level in each case. We present both the (a) coefficients estimated by the regression and (a) their standard errors. There are a variety of noticeable effects, including the overall matching bias in the fast humans (the fact that the intercept coefficients are all negative), the basic content effect that Realitic problems are more likely to yield correct answers, and the finding that language models and slow humans tend to give correct answers more often than fast humans. Additionally, many qualitative patterns reported in Fig. 8 are statistically borne out by this analysis. Note that due to some models rarely giving some responses, certain coefficient estimates are unstable, particularly in the CT,CF row.

C.4 Response time and model log-probability differences

In this section we present the mixed-effects linear regressions comparing human response times and model log-probabilities on the NLI and syllogisms tasks, in Tables S33 and S34, respectively. In order to make these comparisons, we breakdown each problem into cases where both humans and models got it correct, and cases where both got it wrong, and only compare log-

probabilities and response times within these cases. This breakdown is necessary to control for accuracy in these models, as it is a significantly related to both response times and log-probabilities. Note, however, that this means that problems where a model answered correctly but humans never answered correctly, or vice versa, are omitted.

In both tasks, we see significant effects of the content on the model log-probability differences; even controlling for these we see significant relationships to the human response times, such that on items on which the humans respond more slowly, the models show smaller differences in log-probabilities.

```
Linear mixed model fit by REML ['lmerMod']
Formula:
zscored_logprob_diff ~ log(Human) + consistent_plottable + response_correct +
    (1 | model) + (1 | name)
   Data: nli_logprob_rt_corr_df

REML criterion at convergence: 2074.8

Scaled residuals:
    Min      1Q  Median      3Q     Max
-2.7422 -0.6124  0.0099  0.6353  3.8258

Random effects:
 Groups   Name        Variance Std.Dev.
 name     (Intercept) 0.2772   0.5265
 model    (Intercept) 0.0000   0.0000
 Residual             0.5468   0.7394
Number of obs: 831, groups:  name, 171; model, 5

Fixed effects:
                               Estimate Std. Error t value
(Intercept)                      0.7544     0.5353   1.409
log(Human)                      -0.5392     0.1590  -3.392
consistent_plottableConsistent   0.6136     0.1345   4.563
consistent_plottableNonsense    -0.1841     0.1427  -1.291
response_correctTRUE             0.7889     0.2383   3.311
```

Table S33: Statistical analysis of the relationship between human response times and language model log-probability differences on the NLI tasks, using a mixed-effects regression controlling for the task variables and answer correctness, as well as random effects of the item and LM. Note that the model log-probabilities are significantly affected by the content, even though the model accuracy is not.

```
Linear mixed model fit by REML ['lmerMod']
Formula: zscored_logprob_diff ~ log(Human) + logic_belief_consistent +
    consistent_plottable + response_correct + (1 | model) + (1 |
    syllogism_name)
   Data: syllogism_logprob_rt_corr_df_2

REML criterion at convergence: 1077

Scaled residuals:
     Min       1Q   Median       3Q      Max
-2.33241 -0.73009 -0.02953  0.61543  2.99660

Random effects:
 Groups         Name        Variance Std.Dev.
 syllogism_name (Intercept) 0.055391 0.23535
 model          (Intercept) 0.005615 0.07493
 Residual                   0.829587 0.91082
Number of obs: 394, groups:  syllogism_name, 36; model, 5

Fixed effects:
                             Estimate Std. Error t value
(Intercept)                   1.18573    0.70847   1.674
log(Human)                   -0.41458    0.20342  -2.038
logic_belief_consistent       0.08477    0.05899   1.437
consistent_plottableviolate  -0.27369    0.07090  -3.860
consistent_plottablenonsense -0.10094    0.09245  -1.092
response_correctTRUE          0.49560    0.10421   4.756
```

Table S34: Statistical analysis of the relationship between human response times and language model log-probability differences on the Syllogisms tasks, using a mixed-effects regression controlling for the task variables and answer correctness, as well as random effects of the item and LM.

References

[1] Peter Cathcart Wason. Reasoning about a Rule. *Quarterly Journal of Experimental Psychology*, 20:273 – 281, 1968.

[2] Allen Newell. Physical symbol systems. *Cognitive science*, 4(2):135–183, 1980.

[3] Jerry A Fodor and Zenon W Pylyshyn. Connectionism and cognitive architecture: A critical analysis. *Cognition*, 28(1-2):3–71, 1988.

[4] Gary Marcus. The next decade in ai: four steps towards robust artificial intelligence. *arXiv preprint 2002.06177*, 2020.

[5] Melanie Mitchell. Abstraction and analogy-making in artificial intelligence. *Annals of the New York Academy of Sciences*, 1505(1):79–101, 2021.

[6] Jacob Russin, Randall C O'Reilly, and Yoshua Bengio. Deep learning needs a prefrontal cortex. *Work Bridging AI Cogn Sci*, 107:603–616, 2020.

[7] Tom Brown, Benjamin Mann, Nick Ryder, Melanie Subbiah, Jared D Kaplan, Prafulla Dhariwal, Arvind Neelakantan, Pranav Shyam, Girish Sastry, Amanda Askell, et al. Language models are few-shot learners. *Advances in neural information processing systems*, 33:1877–1901, 2020.

[8] Deep Ganguli, Danny Hernandez, Liane Lovitt, Amanda Askell, Yuntao Bai, Anna Chen, Tom Conerly, Nova Dassarma, Dawn Drain, Nelson Elhage, Sheer El Showk, Stanislav Fort, Zac Hatfield-Dodds, Tom Henighan, Scott Johnston, Andy Jones, Nicholas Joseph, Jackson Kernian, Shauna Kravec, Ben Mann, Neel Nanda, Kamal Ndousse, Catherine Olsson, Daniela Amodei, Tom Brown, Jared Kaplan, Sam McCandlish, Christopher Olah, Dario Amodei, and Jack Clark. Predictability and surprise in large generative

models. In *Proceedings of FACCT 2022*, page 1747–1764, 2022. ISBN 9781450393522. doi: 10.1145/3531146.3533229. URL https://doi.org/10.1145/3531146.3533229.

[9] Maxwell Nye, Anders Johan Andreassen, Guy Gur-Ari, Henryk Michalewski, Jacob Austin, David Bieber, David Dohan, Aitor Lewkowycz, Maarten Bosma, David Luan, Charles Sutton, and Augustus Odena. Show Your Work: Scratchpads for Intermediate Computation with Language Models. In *Deep Learning for Code Workshop*, 2022. URL https://openreview.net/forum?id=HBlx2idbkbq.

[10] Takeshi Kojima, Shixiang Shane Gu, Machel Reid, Yutaka Matsuo, and Yusuke Iwasawa. Large Language Models are Zero-Shot Reasoners. In *Advances in Neural Information Processing Systems*, 2022. URL https://openreview.net/forum?id=e2TBb5y0yFf.

[11] Jason Wei, Yi Tay, Rishi Bommasani, Colin Raffel, Barret Zoph, Sebastian Borgeaud, Dani Yogatama, Maarten Bosma, Denny Zhou, Donald Metzler, Ed H. Chi, Tatsunori Hashimoto, Oriol Vinyals, Percy Liang, Jeff Dean, and William Fedus. Emergent Abilities of Large Language Models. *Transactions on Machine Learning Research*, 2022. ISSN 2835-8856. URL https://openreview.net/forum?id=yzkSU5zdwD.

[12] Rylan Schaeffer, Brando Miranda, and Sanmi Koyejo. Are Emergent Abilities of Large Language Models a Mirage? In *Thirty-seventh Conference on Neural Information Processing Systems*, 2023. URL https://openreview.net/forum?id=ITw9edRDlD.

[13] Jack W Rae, Sebastian Borgeaud, Trevor Cai, Katie Millican, Jordan Hoffmann, Francis Song, John Aslanides, Sarah Henderson, Roman Ring, Susannah Young, et al. Scaling

language models: Methods, analysis & insights from training gopher. *arXiv preprint 2112.11446*, 2021.

[14] Yasaman Razeghi, Robert L Logan IV, Matt Gardner, and Sameer Singh. Impact of pretraining term frequencies on few-shot numerical reasoning. In Yoav Goldberg, Zornitsa Kozareva, and Yue Zhang, editors, *Findings of the Association for Computational Linguistics: EMNLP 2022*, pages 840–854, Abu Dhabi, United Arab Emirates, December 2022. Association for Computational Linguistics. doi: 10.18653/v1/2022.findings-emnlp.59. URL `https://aclanthology.org/2022.findings-emnlp.59`.

[15] Arkil Patel, Satwik Bhattamishra, and Navin Goyal. Are NLP models really able to solve simple math word problems? In *Proceedings of the 2021 Conference of the North American Chapter of the Association for Computational Linguistics: Human Language Technologies*, pages 2080–2094, 2021.

[16] Karthik Valmeekam, Alberto Olmo, Sarath Sreedharan, and Subbarao Kambhampati. Large language models still can't plan. *arXiv preprint 2206.10498*, 2022. doi: 10.48550/ARXIV.2206.10498.

[17] Gerd Gigerenzer and Wolfgang Gaissmaier. Heuristic decision making. *Annual review of psychology*, 62(1):451–482, 2011.

[18] Daniel Kahneman, Stewart Paul Slovic, Paul Slovic, and Amos Tversky. *Judgment under uncertainty: Heuristics and biases*. Cambridge university press, 1982.

[19] Gary Marcus. *Kluge: The haphazard evolution of the human mind*. Houghton Mifflin Harcourt, 2009.

[20] JSBT Evans, Julie L Barston, and Paul Pollard. On the conflict between logic and belief in syllogistic reasoning. *Memory & cognition*, 11(3):295–306, 1983.

[21] Jonathan St BT Evans and Tania S Perry. Belief bias in children's reasoning. *Cahiers de Psychologie Cognitive/Current Psychology of Cognition*, 1995.

[22] Karl Christoph Klauer, Jochen Musch, and Birgit Naumer. On belief bias in syllogistic reasoning. *Psychological review*, 107(4):852, 2000.

[23] Philip N Johnson-Laird. Deductive reasoning. *Annual review of psychology*, 50(1):109–135, 1999.

[24] Patricia W Cheng and Keith J Holyoak. Pragmatic reasoning schemas. *Cognitive psychology*, 17(4):391–416, 1985.

[25] Leda Cosmides. The logic of social exchange: Has natural selection shaped how humans reason? Studies with the Wason selection task. *Cognition*, 31(3):187–276, 1989.

[26] Leda Cosmides and John Tooby. Cognitive adaptations for social exchange. *The adapted mind: Evolutionary psychology and the generation of culture*, 163:163–228, 1992.

[27] Andrew L Cohen, Sara Sidlowski, and Adrian Staub. Beliefs and Bayesian reasoning. *Psychonomic Bulletin & Review*, 24(3):972–978, 2017.

[28] Ishita Dasgupta, Eric Schulz, Joshua B Tenenbaum, and Samuel J Gershman. A theory of learning to infer. *Psychological review*, 127(3):412, 2020.

[29] Daniel J Benjamin. Errors in probabilistic reasoning and judgment biases. Technical report, National Bureau of Economic Research, 2018.

[30] Cameron R Peterson and ZJ Ulehla. Uncertainty, inference difficulty, and probability learning. *Journal of Experimental Psychology*, 67:523–530, 1964.

[31] Philip N Johnson-Laird, Paolo Legrenzi, and Maria Sonino Legrenzi. Reasoning and a sense of reality. *British journal of Psychology*, 63(3):395–400, 1972.

[32] Peter Cathcart Wason and Philip Nicholas Johnson-Laird. *Psychology of reasoning: Structure and content*, volume 86. Harvard University Press, 1972.

[33] Jonathan St BT Evans. *Bias in human reasoning: Causes and consequences.* Lawrence Erlbaum Associates, Inc, 1989.

[34] Gary F Marcus. *The algebraic mind: Integrating connectionism and cognitive science*. MIT press, 2003.

[35] James L McClelland, Matthew M Botvinick, David C Noelle, David C Plaut, Timothy T Rogers, Mark S Seidenberg, and Linda B Smith. Letting structure emerge: connectionist and dynamical systems approaches to cognition. *Trends in cognitive sciences*, 14(8): 348–356, 2010.

[36] Adam Santoro, Andrew Lampinen, Kory Mathewson, Timothy Lillicrap, and David Raposo. Symbolic behaviour in artificial intelligence. *arXiv preprint 2102.03406*, 2021.

[37] Rishi Bommasani, Drew A Hudson, Ehsan Adeli, Russ Altman, Simran Arora, Sydney von Arx, Michael S Bernstein, Jeannette Bohg, Antoine Bosselut, Emma Brunskill, et al. On the opportunities and risks of foundation models. *arXiv preprint 2108.07258*, 2021.

[38] Sudeep Bhatia, Russell Richie, and Wanling Zou. Distributed semantic representations for modeling human judgment. *Current Opinion in Behavioral Sciences*, 29:31–36, 2019.

[39] Kanishka Misra, Allyson Ettinger, and Julia Rayz. Do language models learn typicality judgments from text? In *Proceedings of the Annual Meeting of the Cognitive Science Society*, volume 43, 2021.

[40] Trieu H Trinh and Quoc V Le. A simple method for commonsense reasoning. *arXiv preprint 1806.02847*, 2018.

[41] Fabio Petroni, Tim Rocktäschel, Sebastian Riedel, Patrick Lewis, Anton Bakhtin, Yuxiang Wu, and Alexander Miller. Language Models as Knowledge Bases? In *Proceedings of EMNLP 2019 and the 9th International Joint Conference on Natural Language Processing (EMNLP-IJCNLP)*, pages 2463–2473, 2019.

[42] Leo Z Liu, Yizhong Wang, Jungo Kasai, Hannaneh Hajishirzi, and Noah A Smith. Probing across time: What does RoBERTa know and when? *arXiv preprint 2104.07885*, 2021.

[43] Zhengbao Jiang, Jun Araki, Haibo Ding, and Graham Neubig. How can we know when language models know? on the calibration of language models for question answering. *Transactions of the Association for Computational Linguistics*, 9:962–977, 2021.

[44] Anders Søgaard. Locke's holiday: Belief bias in machine reading. In *Proceedings of EMNLP 2021*, pages 8240–8245, 2021.

[45] Jordan Hoffmann, Sebastian Borgeaud, Arthur Mensch, Elena Buchatskaya, Trevor Cai, Eliza Rutherford, Diego de Las Casas, Lisa Anne Hendricks, Johannes Welbl, Aidan Clark, Thomas Hennigan, Eric Noland, Katherine Millican, George van den Driessche, Bogdan Damoc, Aurelia Guy, Simon Osindero, Karén Simonyan, Erich Elsen, Oriol Vinyals, Jack Rae, and Laurent Sifre. An empirical analysis of compute-optimal large language model training. In S. Koyejo, S. Mohamed, A. Agarwal,

D. Belgrave, K. Cho, and A. Oh, editors, *Advances in Neural Information Processing Systems*, volume 35, pages 30016–30030. Curran Associates, Inc., 2022. URL https://proceedings.neurips.cc/paper_files/paper/2022/file/c1e2faff6f588870935f114ebe04a3e5-Paper-Conference.pdf.

[46] OpenAI. GPT-3.5. https://platform.openai.com/docs/models/gpt-3-5, 2023. Retrieved September 19th, 2023.

[47] Rohan Anil, Andrew M Dai, Orhan Firat, Melvin Johnson, Dmitry Lepikhin, Alexandre Passos, Siamak Shakeri, Emanuel Taropa, Paige Bailey, Zhifeng Chen, et al. Palm 2 technical report. *arXiv preprint 2305.10403*, 2023.

[48] Bill MacCartney and Christopher D Manning. Natural logic for textual inference. In *Proceedings of the ACL-PASCAL Workshop on Textual Entailment and Paraphrasing*, pages 193–200, 2007.

[49] Samuel Bowman, Gabor Angeli, Christopher Potts, and Christopher D Manning. A large annotated corpus for learning natural language inference. In *Proceedings of the 2015 Conference on Empirical Methods in Natural Language Processing*, pages 632–642, 2015.

[50] Ishita Dasgupta, Demi Guo, Andreas Stuhlmüller, Samuel J Gershman, and Noah D Goodman. Evaluating compositionality in sentence embeddings. *arXiv preprint 1802.04302*, 2018.

[51] Robin Smith. Aristotle's Logic. In Edward N. Zalta, editor, *The Stanford Encyclopedia of Philosophy*. Metaphysics Research Lab, Stanford University, Fall 2020 edition, 2020.

[52] Michael Henry Tessler, Joshua B Tenenbaum, and Noah D Goodman. Logic, Probability, and Pragmatics in Syllogistic Reasoning. *Topics in Cognitive Science*, 2022.

[53] Chad Dube, Caren M Rotello, and Evan Heit. Assessing the belief bias effect with rocs: it's a response bias effect. *Psychological review*, 117(3):831, 2010.

[54] Dries Trippas, Michael F Verde, and Simon J Handley. Using forced choice to test belief bias in syllogistic reasoning. *Cognition*, 133(3):586–600, 2014.

[55] Michael Henry Tessler. Understanding Belief bias by measuring prior beliefs for a Bayesian model of syllogistic reasoning. *Proceedings of ESSLLI*, pages 225–237, 2015.

[56] Peter C Wason and Diana Shapiro. Natural and contrived experience in a reasoning problem. *Quarterly journal of experimental psychology*, 23(1):63–71, 1971.

[57] James R Cox and Richard A Griggs. The effects of experience on performance in wason's selection task. *Memory & Cognition*, 10:496–502, 1982.

[58] Stephen A Yachanin and Ryan D Tweney. The effect of thematic content on cognitive strategies in the four-card selection task. *Bulletin of the Psychonomic Society*, 19(2): 87–90, 1982.

[59] Matthew Inglis and Adrian Simpson. Mathematicians and the selection task. *International Group for the Psychology of Mathematics Education*, 2004.

[60] Clio Cresswell and Craig P. Speelman. Does mathematics training lead to better logical thinking and reasoning? a cross-sectional assessment from students to professors. *PLOS ONE*, 15(7):1–21, 07 2020. doi: 10.1371/journal.pone.0236153. URL https://doi.org/10.1371/journal.pone.0236153.

[61] Jonathan St BT Evans and Jodie Curtis-Holmes. Rapid responding increases belief bias: Evidence for the dual-process theory of reasoning. *Thinking & Reasoning*, 11(4):382–389, 2005.

[62] J St BT Evans, SE Newstead, JL Allen, and P Pollard. Debiasing by instruction: The case of belief bias. *European Journal of Cognitive Psychology*, 6(3):263–285, 1994.

[63] Wayne A Wickelgren. Speed-accuracy tradeoff and information processing dynamics. *Acta psychologica*, 41(1):67–85, 1977.

[64] Steven L Wise and Xiaojing Kong. Response time effort: A new measure of examinee motivation in computer-based tests. *Applied Measurement in Education*, 18(2):163–183, 2005.

[65] Jason Wei, Xuezhi Wang, Dale Schuurmans, Maarten Bosma, Fei Xia, Ed Chi, Quoc V Le, Denny Zhou, et al. Chain-of-thought prompting elicits reasoning in large language models. *Advances in Neural Information Processing Systems*, 35:24824–24837, 2022.

[66] Gemini Team, Rohan Anil, Sebastian Borgeaud, Yonghui Wu, Jean-Baptiste Alayrac, Jiahui Yu, Radu Soricut, Johan Schalkwyk, Andrew M Dai, Anja Hauth, et al. Gemini: a family of highly capable multimodal models. *arXiv preprint 2312.11805*, 2023.

[67] Gemma Team, Thomas Mesnard, Cassidy Hardin, Robert Dadashi, Surya Bhupatiraju, Shreya Pathak, Laurent Sifre, Morgane Rivière, Mihir Sanjay Kale, Juliette Love, et al. Gemma: Open Models Based on Gemini Research and Technology. *arXiv preprint 2403.08295*, 2024.

[68] Saurav Kadavath, Tom Conerly, Amanda Askell, Tom Henighan, Dawn Drain, and Ethan et al. Perez. Language models (mostly) know what they know, 2022. URL `https://arxiv.org/abs/2207.05221`.

[69] Adam Goodkind and Klinton Bicknell. Predictive power of word surprisal for reading times is a linear function of language model quality. In *Proceedings of the 8th workshop on cognitive modeling and computational linguistics (CMCL 2018)*, pages 10–18, 2018.

[70] Richard Futrell, Ethan Wilcox, Takashi Morita, Peng Qian, Miguel Ballesteros, and Roger Levy. Neural language models as psycholinguistic subjects: Representations of syntactic state. In *Proceedings of the 2019 Conference of the North American Chapter of the Association for Computational Linguistics: Human Language Technologies, Volume 1 (Long and Short Papers)*, pages 32–42, Minneapolis, Minnesota, June 2019. Association for Computational Linguistics. doi: 10.18653/v1/N19-1004. URL https://aclanthology.org/N19-1004.

[71] Marcel Binz and Eric Schulz. Using cognitive psychology to understand gpt-3. *Proceedings of the National Academy of Sciences*, 120(6):e2218523120, 2023. doi: 10.1073/pnas.2218523120. URL https://www.pnas.org/doi/abs/10.1073/pnas.2218523120.

[72] Daniel Khashabi, Chitta Baral, Yejin Choi, and Hannaneh Hajishirzi. Reframing instructional prompts to gptk's language. In *Findings of the Association for Computational Linguistics: ACL 2022*, pages 589–612, 2022.

[73] Andrew Lampinen, Ishita Dasgupta, Stephanie Chan, Kory Mathewson, Mh Tessler, Antonia Creswell, James McClelland, Jane Wang, and Felix Hill. Can language models learn from explanations in context? In *Findings of the Association for Computational Linguistics: EMNLP 2022*, pages 537–563, 2022.

[74] Xuezhi Wang, Jason Wei, Dale Schuurmans, Quoc Le, Ed Chi, and Denny Zhou. Self-consistency improves chain of thought reasoning in language models. *arXiv preprint 2203.11171*, 2022.

[75] Hugo Mercier and Dan Sperber. *The Enigma of Reason*. Harvard University Press, 2017.

[76] Jonathan St BT Evans. Heuristic and analytic processes in reasoning. *British Journal of Psychology*, 75(4):451–468, 1984.

[77] Jonathan St BT Evans. In two minds: dual-process accounts of reasoning. *Trends in cognitive sciences*, 7(10):454–459, 2003.

[78] Mike Oaksford and Nick Chater. Optimal data selection: Revision, review, and reevaluation. *Psychonomic Bulletin & Review*, 10(2):289–318, 2003.

[79] Daniel Kahneman. *Thinking, fast and slow*. Macmillan, 2011.

[80] Jonathon St BT Evans and David E Over. *Rationality and reasoning*. Psychology Press, 2013.

[81] Yoshua Bengio. The consciousness prior. *arXiv preprint 1709.08568*, 2017.

[82] Maxwell Nye, Michael Tessler, Josh Tenenbaum, and Brenden M Lake. Improving coherence and consistency in neural sequence models with dual-system, neuro-symbolic reasoning. *Advances in Neural Information Processing Systems*, 34:25192–25204, 2021.

[83] Matthew M Botvinick and Jonathan D Cohen. The computational and neural basis of cognitive control: charted territory and new frontiers. *Cognitive science*, 38(6):1249–1285, 2014.

[84] John Duncan, Moataz Assem, and Sneha Shashidhara. Integrated intelligence from distributed brain activity. *Trends in Cognitive Sciences*, 24(10):838–852, 2020.

[85] Daniel LK Yamins, Ha Hong, Charles F Cadieu, Ethan A Solomon, Darren Seibert, and James J DiCarlo. Performance-optimized hierarchical models predict neural responses in higher visual cortex. *Proceedings of the national academy of sciences*, 111(23):8619–8624, 2014.

[86] Daniel LK Yamins and James J DiCarlo. Using goal-driven deep learning models to understand sensory cortex. *Nature neuroscience*, 19(3):356–365, 2016.

[87] Rosa Cao and Daniel Yamins. Explanatory models in neuroscience: Part 1–taking mechanistic abstraction seriously. *arXiv preprint 2104.01490*, 2021.

[88] Rosa Cao and Daniel Yamins. Explanatory models in neuroscience: Part 2–constraint-based intelligibility. *arXiv preprint 2104.01489*, 2021.

[89] Martin Schrimpf, Idan Asher Blank, Greta Tuckute, Carina Kauf, Eghbal A Hosseini, Nancy Kanwisher, Joshua B Tenenbaum, and Evelina Fedorenko. The neural architecture of language: Integrative modeling converges on predictive processing. *Proceedings of the National Academy of Sciences*, 118(45), 2021.

[90] Sreejan Kumar, Theodore R Sumers, Takateru Yamakoshi, Ariel Goldstein, Uri Hasson, Kenneth A Norman, Thomas L Griffiths, Robert D Hawkins, and Samuel A Nastase. Reconstructing the cascade of language processing in the brain using the internal computations of a transformer-based language model. *bioRxiv*, 2022.

[91] Ariel Goldstein, Zaid Zada, Eliav Buchnik, Mariano Schain, Amy Price, Bobbi Aubrey, Samuel A Nastase, Amir Feder, Dotan Emanuel, Alon Cohen, et al. Shared computational principles for language processing in humans and deep language models. *Nature neuroscience*, 25(3):369–380, 2022.

[92] Ethan Gotlieb Wilcox, Jon Gauthier, Jennifer Hu, Peng Qian, and Roger Levy. On the predictive power of neural language models for human real-time comprehension behavior. *arXiv preprint 2006.01912*, 2020.

[93] Falk Lieder and Thomas L Griffiths. Resource-rational analysis: Understanding human

cognition as the optimal use of limited computational resources. *Behavioral and brain sciences*, 43, 2020.

[94] Samuel J Gershman, Eric J Horvitz, and Joshua B Tenenbaum. Computational rationality: A converging paradigm for intelligence in brains, minds, and machines. *Science*, 349 (6245):273–278, 2015.

[95] Herbert A Simon. Bounded rationality. In *Utility and probability*, pages 15–18. Springer, 1990.

[96] Tal Linzen. How can we accelerate progress towards human-like linguistic generalization? In Dan Jurafsky, Joyce Chai, Natalie Schluter, and Joel Tetreault, editors, *Proceedings of the 58th Annual Meeting of the Association for Computational Linguistics*, pages 5210–5217, Online, July 2020. Association for Computational Linguistics. doi: 10.18653/v1/2020.acl-main.465. URL `https://aclanthology.org/2020.acl-main.465`.

[97] Albert Webson and Ellie Pavlick. Do Prompt-Based Models Really Understand the Meaning of Their Prompts? In *Proceedings of the 2022 Conference of the North American Chapter of the Association for Computational Linguistics: Human Language Technologies*, pages 2300–2344, 2022.

[98] Swaroop Mishra, Daniel Khashabi, Chitta Baral, and Hannaneh Hajishirzi. Cross-task generalization via natural language crowdsourcing instructions. *arXiv preprint 2104.08773*, 2021.

[99] James L McClelland, Felix Hill, Maja Rudolph, Jason Baldridge, and Hinrich Schütze. Placing language in an integrated understanding system: Next steps toward human-level

performance in neural language models. *Proceedings of the National Academy of Sciences*, 117(42):25966–25974, 2020.

[100] Martha W Alibali, Rebecca Boncoddo, and Autumn B Hostetter. Gesture in reasoning: An embodied perspective. *Routledge handbook of embodied cognition*, page 150, 2014.

[101] Mitchell J Nathan, Kelsey E Schenck, Rebecca Vinsonhaler, Joseph E Michaelis, Michael I Swart, and Candace Walkington. Embodied geometric reasoning: Dynamic gestures during intuition, insight, and proof. *Journal of Educational Psychology*, 2020.

[102] Herbert H Clark. *Using language*. Cambridge university press, 1996.

[103] David Schlangen. Norm Participation Grounds Language. In *Proceedings of the 2022 CLASP Conference on (Dis) embodiment*, pages 62–69, 2022.

[104] Alexander K Luria. Towards the Problem of the Historical Nature of Psychological Processes. *International Journal of Psychology*, 6(4):259–272, 1971.

[105] Darrin R Lehman and Richard E Nisbett. A longitudinal study of the effects of undergraduate training on reasoning. *Developmental Psychology*, 26(6):952, 1990.

[106] Nina Attridge, Andrew Aberdein, and Matthew Inglis. Does studying logic improve logical reasoning? 2016.

[107] Andrew Joohun Nam and James L McClelland. What underlies rapid learning and systematic generalization in humans. *arXiv preprint 2107.06994*, 2021.

[108] Peter Clark, Oyvind Tafjord, and Kyle Richardson. Transformers as soft reasoners over language. *arXiv preprint 2002.05867*, 2020.

[109] Yuhuai Wu, Markus N Rabe, Wenda Li, Jimmy Ba, Roger B Grosse, and Christian Szegedy. LIME: Learning inductive bias for primitives of mathematical reasoning. In *International Conference on Machine Learning*, pages 11251–11262. PMLR, 2021.

[110] Timo Schick, Sahana Udupa, and Hinrich Schütze. Self-Diagnosis and Self-Debiasing: A Proposal for Reducing Corpus-Based Bias in NLP. *Transactions of the Association for Computational Linguistics*, 9:1408–1424, 12 2021. ISSN 2307-387X. doi: 10.1162/tacl_a_00434. URL `https://doi.org/10.1162/tacl\_a\_00434`.

[111] Karl Cobbe, Vineet Kosaraju, Mohammad Bavarian, Jacob Hilton, Reiichiro Nakano, Christopher Hesse, and John Schulman. Training verifiers to solve math word problems. *arXiv preprint 2110.14168*, 2021.

[112] William Saunders, Catherine Yeh, Jeff Wu, Steven Bills, Long Ouyang, Jonathan Ward, and Jan Leike. Self-critiquing models for assisting human evaluators. *arXiv preprint 2206.05802*, 2022.

[113] Eric Zelikman, Yuhuai Wu, Jesse Mu, and Noah Goodman. Star: Bootstrapping reasoning with reasoning. *Advances in Neural Information Processing Systems*, 35:15476–15488, 2022.

[114] Jane X Wang. Meta-learning in natural and artificial intelligence. *Current Opinion in Behavioral Sciences*, 38:90–95, 2021.

[115] Stephanie Chan, Adam Santoro, Andrew Lampinen, Jane Wang, Aaditya Singh, Pierre Richemond, James McClelland, and Felix Hill. Data distributional properties drive emergent in-context learning in transformers. *Advances in Neural Information Processing Systems*, 35:18878–18891, 2022.

[116] Ben Prystawski, Michael Li, and Noah Goodman. Why think step by step? Reasoning emerges from the locality of experience. *Advances in Neural Information Processing Systems*, 36, 2024.

[117] Ali Emami, Kaheer Suleman, Adam Trischler, and Jackie Chi Kit Cheung. An Analysis of Dataset Overlap on Winograd-Style Tasks. In *Proceedings of the 28th International Conference on Computational Linguistics*, pages 5855–5865, 2020.

[118] Jesse Dodge, Maarten Sap, Ana Marasović, William Agnew, Gabriel Ilharco, Dirk Groeneveld, Margaret Mitchell, and Matt Gardner. Documenting large webtext corpora: A case study on the colossal clean crawled corpus. In *Proceedings of EMNLP 2021*, pages 1286–1305, Online and Punta Cana, Dominican Republic, November 2021. Association for Computational Linguistics. doi: 10.18653/v1/2021.emnlp-main.98. URL https://aclanthology.org/2021.emnlp-main.98.

[119] Yi Tay, Mostafa Dehghani, Vinh Q Tran, Xavier Garcia, Jason Wei, Xuezhi Wang, Hyung Won Chung, Dara Bahri, Tal Schuster, Steven Zheng, et al. Ul2: Unifying language learning paradigms. In *The Eleventh International Conference on Learning Representations*, 2022.

[120] Ari Holtzman, Peter West, Vered Shwartz, Yejin Choi, and Luke Zettlemoyer. Surface form competition: Why the highest probability answer isn't always right. In *Proceedings of the 2021 Conference on Empirical Methods in Natural Language Processing*, pages 7038–7051, 2021.

[121] Ari Holtzman, Jan Buys, Li Du, Maxwell Forbes, and Yejin Choi. The Curious Case of Neural Text Degeneration. In *International Conference on Learning Representations*, 2019.

[122] Arian Hosseini, Siva Reddy, Dzmitry Bahdanau, R Devon Hjelm, Alessandro Sordoni, and Aaron Courville. Understanding by understanding not: Modeling negation in language models. In *Proceedings of the 2021 Conference of the North American Chapter of the Association for Computational Linguistics: Human Language Technologies*, pages 1301–1312, Online, June 2021. Association for Computational Linguistics. doi: 10.18653/v1/2021.naacl-main.102. URL `https://aclanthology.org/2021.naacl-main.102`.

[123] Jonathan St BT Evans, John Clibbens, and Benjamin Rood. The role of implicit and explicit negation in conditional reasoning bias. *Journal of Memory and Language*, 35 (3):392–409, 1996.

[124] Hyung Won Chung, Le Hou, Shayne Longpre, Barret Zoph, Yi Tay, William Fedus, Yunxuan Li, Xuezhi Wang, Mostafa Dehghani, Siddhartha Brahma, et al. Scaling instruction-finetuned language models. *arXiv preprint 2210.11416*, 2022.
